# HealthProcessAI: a technical framework and proof-of-concept for LLM-enhanced healthcare process mining

**DOI:** 10.3389/frai.2026.1716819

**Published:** 2026-01-30

**Authors:** Eduardo Illueca-Fernandez, Kaile Chen, Fernando Seoane, Farhad Abtahi

**Affiliations:** 1 Department of Clinical Science, Intervention and Technology, Karolinska Institutet, Stockholm, Sweden; 2 Department of Biomedical Engineering and Health System, KTH Royal Institute of Technology, Huddinge, Sweden; 3 Department of Clinical Physiology, Karolinska University Hospital, Stockholm, Sweden; 4 Department of Textile Technology, University of Borås, Borås, Sweden; 5 Department of Medical Technologies, Karolinska University Hospital, Huddinge, Sweden

**Keywords:** epidemiology, generative AI, healthcare analytics, large language models, process mining

## Abstract

**Background:**

Process mining has emerged as a powerful analytical technique for understanding complex healthcare workflows. However, its application faces significant barriers, including technical complexity, a lack of standardized approaches, and limited access to practical training resources. To address unfamiliarity and improve accessibility, we proposed a new framework for translating technical analyses into text outputs that users can understand.

**Objective:**

We introduce HealthProcessAI, a GenAI framework designed to simplify process mining applications in healthcare and epidemiology by providing a comprehensive wrapper around existing Python (PM4PY) and R (bupaR) libraries. To address unfamiliarity and improve accessibility, the framework integrates multiple Large Language Models (LLMs) for automated process map interpretation and report generation, helping translate technical analyses into outputs that diverse users can readily understand.

**Methods:**

HealthProcessAI implements modular architecture with the following components: (1) data loading and preparation, (2) process mining analysis, (3) integration of LLM for interpretation, (4) advanced analytics, (5) multimodal report orchestration, and (6) the validation framework. We validated the framework using sepsis progression data as a proof-of-concept example and compared the outputs of five state-of-the-art LLM models through the OpenRouter platform. This study presents a technical validation using automated LLM evaluation, and clinical validation by healthcare professionals is planned as future work.

**Results:**

To test its functionality, the framework successfully processed sepsis data across four proof-of-concept cases. A total of 32 reports were generated, demonstrating robust technical performance and its capability to generate reports through automated LLM analysis. In concrete terms, there are eight reports per case and four reports per LLM model. LLM evaluation using seven independent LLMs as automated evaluators revealed distinct model strengths: Claude Sonnet-4 and Gemini 2.5-Pro achieved the highest consistency scores (3.72/4.0 and 3.49/4.0) when evaluated by automated LLM assessors. It is important to note that outputs were not clinically validated by healthcare professionals.

**Conclusion:**

HealthProcessAI provides a standardized framework that reduces technical and training barriers in healthcare process mining while maintaining scientific objectivity. By integrating multiple LLMs for automated interpretation and report generation, the framework addresses widespread unfamiliarity with process mining outputs, demonstrating technical feasibility for making them more accessible to clinicians, data scientists and researchers pending clinical validation. This structured analytics and AI-driven interpretation combination represents a novel methodological advance in translating complex process mining results into potentially actionable insights for healthcare applications. However, future work should involve systematic evaluation by clinicians.

## 1 Introduction

The ongoing digitalization of healthcare systems worldwide generates substantial volumes of unstructured data through electronic health records (EHRs), clinical information systems, laboratory results, and patient monitoring devices (Modi et al., 2024). These data encapsulate complex patient journeys and clinical workflows, offering significant potential to improve healthcare quality and outcomes. Despite global healthcare expenditures averaging approximately 10% of GDP and increasing accessibility of electronic data, clinicians continue to face limited access to practical tools for interpreting these complex datasets (Wibawa et al., 2024). Process mining is a discipline at the intersection of data mining and business process management (Van der Aalst, 2016), which has shown potential as a powerful method for extracting insights from event logs in healthcare (Rojas et al., 2016; Muñoz-Gama et al., 2022).

The application of process mining in healthcare has shown substantial promise in various domains, including emergency department workflows (Samara and Harry, 2025), surgical procedures (Kurniati et al., 2019), and chronic disease progression (Chen et al., 2024a). Process mining has evolved from business process management to healthcare applications since the early 2000s, enabling the discovery, conformance checking, and enhancement of clinical pathways across over 270 healthcare studies analyzed in recent systematic reviews (Ghasemi and Amyot, 2016).

Nevertheless, several critical barriers hinder its broader implementation in clinical practice. First, the technical complexity of existing tools demands expertise that many healthcare professionals and data scientists do not possess (Erdogan and Tarhan, 2018). Second, interpreting process mining outputs often requires a deep understanding of algorithmic principles and clinical contexts, presenting a knowledge gap that limits usability. Third, a lack of standardization and comprehensive educational frameworks contributes to methodological heterogeneity, hindering reproducibility and cross-study comparisons.

Recent advancements in large language models (LLMs) offer novel opportunities to bridge these gaps (Brown et al., 2020; Lee et al., 2023). LLMs have demonstrated remarkable capabilities in comprehending complex medical language and contextualizing heterogeneous healthcare data (Singhal et al., 2023). However, their integration with process mining methodologies remains largely unexplored, particularly in clinical decision support and educational applications.

Despite the maturity of process mining in analyzing patient flows, such as in oncology, mental health services, and emergency care, the interpretability of outputs remains limited (Mans et al., 2015). Emerging platforms like OpenRouter (OpenRouter, Inc., CA, United States) have democratized access to multiple LLM providers, enabling multimodal experimentation and cost-effective deployment, thus creating new possibilities for the synergistic use of LLMs and process mining. This convergence opens a unique opportunity: to develop frameworks that integrate the analytical rigor of process mining with the semantic and interpretive capabilities of modern AI systems. Such integration directly addresses current limitations in clinical interpretability, which remains a persistent challenge in healthcare analytics.

Current process mining tools such as PM4PY (Berti et al., 2019) and bupaR (Janssenswillen et al., 2019) generate sophisticated analytical outputs but require substantial programming knowledge, impeding adoption among healthcare practitioners. Moreover, the outputs often lack direct clinical relevance and are rarely translated into actionable insights. To address these limitations, this study proposes a novel approach leveraging LLMs to transform process mining outputs into clinically interpretable reports enriched with structured outputs. This AI-enhanced framework offers an interpretable layer on top of complex data models by maintaining the relationships between clinical processes and entities. Such systems have shown potential in integrating and analyzing fragmented healthcare data, facilitating more informed and timely decision-making.

We hypothesize that it is possible to transform process mining results into semantically rich, clinically interpretable reports using LLMs. This transformation requires the definition of a structured educational framework tailored for healthcare professionals and researchers. Through LLM-based reasoning, process mining datasets can be linked to broader healthcare knowledge bases, allowing clinical pathways to be associated with outcomes or care quality metrics via evidence-based mechanisms. We present the first comprehensive framework for LLM-enhanced healthcare process mining to evaluate this hypothesis. Our contributions are threefold:

1. Multi-LLM Interpretation Methodology: We introduce a multi-model approach for the automated interpretation of process mining results, with potential generalizability beyond healthcare applications.
2. Structured Framework for Accessibility and Reproducibility: We design an integrated framework that addresses the technical and educational barriers limiting adoption, promoting accessibility and methodological reproducibility.
3. Empirical Demonstration in Proof-of-Concept Cases: We demonstrate our framework’s functionality through a proof-of-concept analysis of sepsis progression and kidney disease, a complex, high-risk clinical pathway used here to test the system’s capabilities.

The remainder of this paper is structured as follows. The Methodology section outlines the analytical approach for process mining, detailing how process maps are transformed into clinically interpretable reports. It also describes the architecture of the proposed framework and the evaluation methods applied. Privacy-preserving deployment strategies are discussed in Section 2.5. The Results section presents a comparative analysis of process mining tools implemented in R and Python. It evaluates the quality and interpretability of the generated reports within demonstrator case studies. The Discussion section contextualizes these findings by comparing them with current literature, highlighting key insights, implications, and potential limitations. Finally, the Conclusions section summarizes the core contributions of the study and identifies future challenges and directions for the continued development of LLM-integrated process mining in healthcare.

## 2 Methods

### 2.1 Framework design and architecture

**Figure 1:**
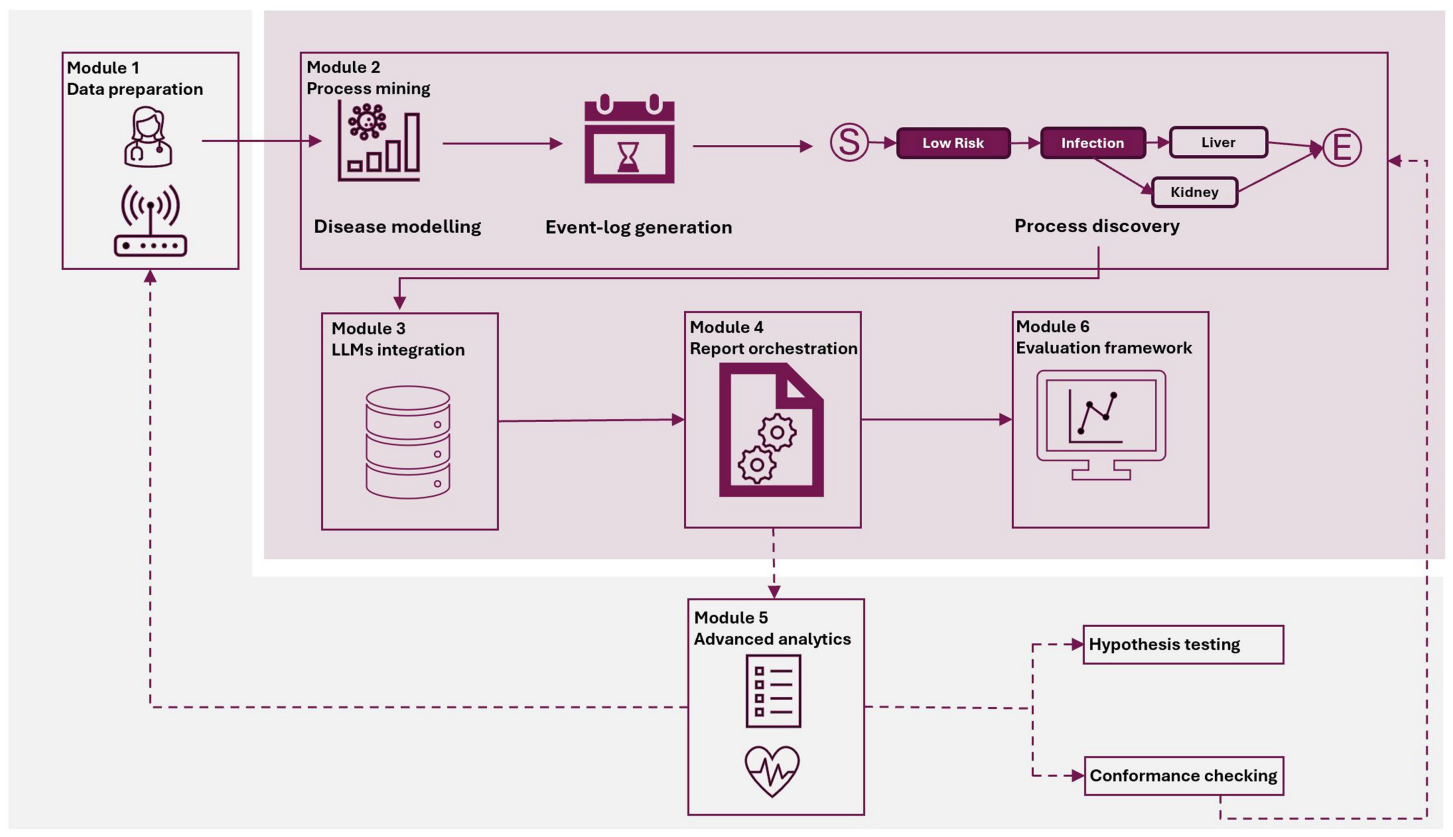
Modular architecture of the HealthProcessAI pipeline. The six-module architecture supports end-to-end healthcare process mining, from data preparation and event log generation to process discovery and LLM-driven interpretation. It includes modules for report customization, system integration, and evaluation. Only the process matrix obtained after process discovery was sent to the API. The architecture is compatible with both Python and R and incorporates educational components to facilitate adoption among healthcare professionals and researchers. Solid lines indicate components implemented in this study, while dashed lines represent ongoing research and development.

HealthProcessAI is a framework built upon established process mining libraries, specifically designed for healthcare and epidemiological applications. Its architecture follows recognized software design patterns used in healthcare informatics systems (Shortliffe and Cimino, 2014) while introducing novel approaches to AI integration. The development of the framework was guided by four core principles, emphasizing comprehensive educational support and clinical applicability. The first principle, accessibility, ensures that all components are accompanied by detailed documentation, defined learning objectives, and step-by-step tutorials aligned with medical education standards. The framework maintains technology-agnostic flexibility by supporting both Python and R implementations, accommodating diverse user preferences and institutional infrastructures. Finally, AI-enhanced interpretation is realized by integrating multiple LLMs, enabling automated clinical report generation and contextual analysis of complex healthcare processes. HealthProcessAI adopts a modular pipeline architecture grounded in established process mining methodologies, as depicted in Figure 1 (Van der Aalst et al., 2011).

In concrete, HealthProcessAI is based on the interactive process mining framework for epidemiology (Fernandez-Llatas, 2020; Chen et al., 2024a), which is also extension of the question driven methodology (Rojas et al., 2017). Furthermore, our approach is linked also with the PM^2^ framework, ensuring alignment with established best practices in processing mining (Van Eck et al., 2015). Module 1 (Data Loading and Preparation) implements PM^2^ Stages 1–2 by handling project planning considerations and data extraction from healthcare systems. Module 2 (Process Mining Analysis) addresses PM2 Stage 3 through comprehensive process discovery and conformance checking. Modules 3–4 (LLM Integration and Report Orchestration) enhance PM^2^ Stage 4 by providing automated interpretation and evaluation of discovered processes. Module 5 (Advanced Analytics) supports PM^2^ Stages 5–6 by enabling process improvement and operational support through actionable insights.

This paper focuses on technical validation of the framework, proving its feasibility and demonstrating that HealthProcessAI can generate reports from real process maps. The different modules implemented are highlighted with a purple background in Figure 1. Regarding the evaluation framework, it is a component that has been developed for the demonstrators implemented in this paper, and it is not necessary for future implementations. However, out of scope is the clinical validation of our framework and its application in other process mining domains. In concrete terms, advanced analytics and their application to hypothesis testing or conformance checking are not involved in our evaluation, and they will be addressed in future work. We demonstrate functionality by implementing four proof-of-concept cases from two different sources: (1) publicly available PhysioNet Challenge data for technical testing, and (2) previously published process maps from the SCREAM database for comparison. These proof-of-concept cases establish technical feasibility before planned clinical validation studies.

#### 2.1.1 Module 1: data loading and preparation

The data loading module implements healthcare-specific data preparation techniques based on clinical informatics standards, incorporating several key features that enhance its utility for medical data processing (Benson and Grieve, 2016). The module handles event logs stored in CSV format, ensuring compatibility with diverse clinical information systems. It implements comprehensive data quality checks specifically designed to meet clinical data validation rules, helping maintain the integrity and reliability of healthcare datasets. Additionally, the module provides standardized column naming conventions that follow international healthcare standards, promoting consistency and interoperability across different clinical contexts. The system also includes healthcare-specific filtering methods that facilitate clinical cohort identification, enabling researchers and clinicians to isolate relevant patient populations for analysis and study efficiently.

For the sepsis use case, the data quality module was configured with a targeted set of validation rules designed to ensure both structural integrity and clinical plausibility before process discovery. Structural validation primarily focused on temporal consistency, enforcing strict chronological ordering of events within a trace and verifying that discharge timestamps logically succeeded admission timestamps. Additionally, we enforced identifier consistency and record completeness, rejecting any entries that lacked mandatory process mining attributes (specifically Case ID, Activity Label, or Timestamp) or that represented duplicate event signatures. To ensure clinical validity, the pipeline implemented value range constraints based on established physiological bounds, filtering out measurement artifacts such as body temperatures exceeding 45 °C. Finally, healthcare-specific cohort filters were applied to refine the dataset, selecting only those patient trajectories that possessed the complete set of longitudinal biomarker measurements required for the study’s specific sepsis definition.

For SCREAM database, we used published process maps, so data loading and preparation is described in previous articles from our research group (Chen et al., 2024a,b).

#### 2.1.2 Module 2: process mining analysis

The process mining module is a comprehensive wrapper around *PM4PY* (Berti et al., 2019) and *bupaR* (Janssenswillen et al., 2019), providing healthcare-optimized algorithms encompassing many process discovery and analysis techniques. The module supports several key algorithms, including Directly-Follows Graph (DFG) discovery (Van der Aalst et al., 2004), Heuristics Miner for noise-tolerant healthcare processes (Weijters et al., 2006), Alpha Algorithm for structured clinical protocols, Inductive Miner for sound process models (Leemans et al., 2013), and performance analysis with standard quality metrics In concrete, we have evaluated the process mining algorithms using the F1-Score, as detailed in Section 2.2. These enhancements are designed to be technically robust, relevant, and applicable to real-world healthcare scenarios.

#### 2.1.3 Module 3: LLM integration

The LLM integration module provides standardized interfaces to multiple language models, implementing best practices for AI in healthcare (Topol, 2019), and encompasses a comprehensive suite of advanced language models, each optimized for specific clinical applications. The module supports Anthropic Claude (Sonnet-4), which is optimized for clinical reasoning, OpenAI GPT-4.1 with its broad medical knowledge base, Google Gemini 2.5 Pro featuring a large context window for comprehensive analysis, DeepSeek R1 for technical precision and quantitative analysis, and X-AI Grok-4 for creative insights and alternative perspectives. The framework incorporates prompts adapted for healthcare context. This comprehensive approach was designed in the sense that language model integration not only leverages the unique strengths of each AI system but also aligns with medical communication structures.

Prompt templates were engineered following a robust five-component structure to ensure clinical relevance and analytical depth. First, role definition established a specialized role as a process mining analyst applied to epidemiology equipped with the skills to communicate complex data to clinical stakeholders. Second, task specification clearly defined the analytical objective, requiring the synthesis of provided process matrices and visual maps into a comprehensive report. Third, context provision embedded essential domain knowledge, including the target audience definition and specific disease state logic (e.g., detailing reversible transitions between “Infection” and “Sepsis” states). Fourth, output format requirements mandated a strict Markdown structure comprising six standardized sections: Executive Summary, Introduction, Process Map Analysis, Data Summary Tables, Hypothesis Generation, and Conclusion. Finally, quality criteria enforced professional, collaborative tone and specific formatting guidelines (e.g., use of bullet points and bold text) to maximize readability and actionability. Complete prompt templates for all four case studies are provided in Supplementary Tables S2–S5.

#### 2.1.4 Module 4: report orchestration

The orchestration module implements multi-model consensus techniques adapted from ensemble learning principles, providing sophisticated mechanisms for integrating and synthesizing insights from multiple analytical sources. Rather than relying on a single Large Language Model (LLM) output, which may be prone to stochastic variations, the orchestration engine synthesizes findings from multiple independent analytical sources. The module synthesizes consensus findings using voting mechanisms, preserves unique insights from each model through diversity preservation techniques, identifies areas of agreement and disagreement using inter-rater reliability measures, and creates comprehensive multi-model reports with uncertainty quantification. This approach ensures that the final analytical output captures the collective wisdom of multiple models and maintains transparency regarding the level of consensus and uncertainty in the findings, thereby providing healthcare professionals with a nuanced understanding of the analytical results and the level of consensus supporting each insight.

To mitigate the risk of *semantic hallucination*, the module utilizes a *deterministic constraint injection* strategy within the system prompts. As demonstrated in the Sepsis Progression use case, the prompt strictly defines the model’s role as an *expert process mining analyst* and restricts the generation space to the provided process matrix and map data. Explicit domain constraints are injected into the context window to prevent the fabrication of non-existent clinical states; for instance, the prompt rigidly defines the valid state transitions as (i) *low temperature*, (ii) *normal temperature*, (iii) *high temperature*, (iv) *infection*, and (v) *sepsis*, noting that transitions are reversible.

Furthermore, the orchestration module enforces structural consistency to facilitate programmatic comparison of outputs. The system prompt mandates a strict *Markdown* schema, requiring specific sections such as *Case Summary*, *Activity Summary*, and *Trace Summary*. By constraining the output format and enforcing a collaborative tone targeted at clinical and epidemiological stakeholders, the framework ensures that the multi-model synthesis captures the collective wisdom of the ensemble while maintaining a high degree of interpretability. Divergences between models are not discarded but are preserved as uncertainty indicators, providing healthcare professionals with a nuanced understanding of where the data supports definitive conclusions versus where interpretation varies.

#### 2.1.5 Module 5: advanced analytics

The advanced analytics module showcases some of the research-grade methodologies from recent healthcare process mining literature, incorporating a comprehensive range of analytical capabilities designed to enhance clinical decision-making and operational efficiency from modules 1, 2, 3, and 5 outputs. The module would provide conformance checking with clinical guidelines (Muñoz-Gama and Carmona, 2010), patient stratification analysis using machine learning techniques, bottleneck identification for healthcare optimization, predictive process monitoring for early warning systems, and clinical performance indicators aligned with established quality measures. These capabilities collectively enable healthcare organizations to systematically analyze their processes, identify areas for improvement, predict potential issues before they occur, and maintain compliance with clinical standards while optimizing patient care delivery and operational workflows. For this proof-of-concept demonstration, we primarily implemented process discovery, performance analysis, and basic pathway optimization capabilities linked to *Module 2*. Advanced features such as predictive process monitoring and comprehensive conformance checking represent framework capabilities that will be demonstrated in future work with additional case studies in a pre-operational environment.

### 2.2 Comparing process mining algorithms with F-score

The F1 score in process mining serves as a balanced quality metric that combines two fundamental dimensions of process model evaluation: fitness and precision. Fitness, also known as recall in the context of conformance checking, measures the ability of a discovered process model to reproduce the behavior observed in the event log, quantifying how well the model can replay all traces without blocking. Precision, conversely, assesses the degree to which the model avoids allowing behavior that was not observed in the log, preventing overgeneralization and ensuring that the model does not permit excessive additional execution paths (Muñoz-Gama and Carmona, 2010). The token-based replay technique is commonly employed to calculate fitness by counting missing and remaining tokens during trace replay on the Petri net model, while precision is often evaluated through behavioral comparison techniques such as escaping arcs or footprint-based methods.

The F1 score is computed as the harmonic mean of fitness and precision, formally defined as F1 = 2 × (Precision × Fitness)/(Precision + Fitness), providing a single aggregate measure that balances both quality dimensions (Buijs et al., 2012). This metric is particularly valuable when comparing different process discovery algorithms, as it penalizes models that excel in only one dimension while performing poorly in the other. For instance, a flower model that allows all possible behavior would achieve perfect fitness but very low precision, resulting in a poor F1 score. The use of the harmonic mean, rather than the arithmetic mean, ensures that both metrics must be reasonably high to achieve a good F1 score, making it a robust indicator of overall model quality in automated process discovery evaluations.

### 2.3 LLM model integration via OpenRouter platform

The framework integrates eight state-of-the-art language models through the OpenRouter platform, implementing a novel approach to multi-model healthcare AI systems. OpenRouter serves as a unified gateway providing a single endpoint following RESTful API design principles, cost optimization through competitive pricing via platform economics, and access to the latest models with automated updates. The platform also offers intelligent rate limit management through load balancing, queue management systems, and comprehensive usage analytics tracking that aligns with healthcare AI governance requirements, creating a robust infrastructure for multi-model AI orchestration in clinical applications. Table 1 shows the main characteristics of the five models included in this study.

**Table 1:** Specifications and integration characteristics of selected large language models (LLMs) used within the HealthProcessAI framework.

Model	Provider	Context window	Cost tokens (*)	Primary strengths	Clinical applications
CLAUDE SONNET-4	Anthropic	200 K tokens	$3.00/$15.00	Clinical reasoning, guideline interpretation	Complex diagnostic pathways
GPT-4.1	OpenAI	128 K tokens	$10.00/$30.00	Broad medical knowledge, consistency	General clinical analysis
GEMINI 2.5 PRO	Google	1 M tokens	$1.25/$5.00	Large context, comprehensive analysis	Long clinical narratives
DEEPSEEK R1	DeepSeek	64 K tokens	$0.55/$2.19	Technical precision, quantitative analysis	Statistical interpretation
GROK-4	X-AI	128 K tokens	$5.00/$15.00	Creative insights, patient perspectives	Alternative viewpoints

*Token pricing is reported per 1 million input/output tokens as of August 2025, with the first price referring to input and the second to output tokens. The models vary in context window size, token pricing, and domain strengths. Integration is tailored to clinical use cases such as diagnostic reasoning, long-form medical narratives, statistical interpretation, and patient-centered analysis.

### 2.4 Evaluation framework

We developed a comprehensive evaluation rubric based on healthcare informatics evaluation frameworks and clinical reporting standards (Friedman and Wyatt, 2006), establishing six key criteria for assessing LLM report quality with specific weightings and validation methods (Table 2) defined in previous studies regarding evaluation in AI (Rammal, 2024). The evaluation framework assigns Clinical Accuracy (25%) to determine the correctness of medical interpretations and terminology usage through expert clinical review, Process Mining Understanding (20%) to evaluate accurate interpretation of analytical results via technical validation, Actionable Insights (20%) to measure the quality and feasibility of clinical recommendations through implementation assessment, Statistical Interpretation (15%) to verify correct analysis of quantitative findings using statistical validation, Report Structure and Clarity (10%) to examine organization and readability through communication analysis, and Evidence-Based Reasoning (10%) to evaluate the use of clinical evidence and literature via evidence synthesis evaluation. It is critical to note that this evaluation framework represents technical validation of the system’s functionality and consistency, not clinical validation of output accuracy or utility. No clinician-based review of the generated reports was conducted in this proof-of-concept study.

**Table 2:** Evaluation criteria for assessing LLM-generated clinical reports within the HealthProcessAI framework.

Criterion	Weight	Description	Validation method
Clinical accuracy	25%	Correctness of medical interpretations and terminology usage	Expert clinical review
Process mining understanding	20%	Accurate interpretation of analytical results	Technical validation
Actionable insights	20%	Quality and feasibility of clinical recommendations	Implementation assessment
Statistical interpretation	15%	Correct analysis of quantitative findings	Statistical validation
Report structure & clarity	10%	Organization and readability	Communication analysis
Evidence-based reasoning	10%	Use of clinical evidence and literature	Evidence synthesis evaluation

*Current validation uses automated LLM evaluation rather than clinical validation. Each criterion is assigned a relative weight based on its importance to clinical utility and interpretability. Validation methods involve domain-specific assessments, including clinical expert review, technical accuracy checks, and implementation feasibility testing.

The evaluation criteria weightings were established through iterative refinement with the research team and align with healthcare informatics evaluation frameworks (Friedman and Wyatt, 2006). Clinical accuracy received the highest weight (25%) given the paramount importance of medical correctness in healthcare applications. Process mining understanding and actionable insights were equally weighted (20% each) as core framework objectives. These weights represent our assessment of relative importance for this proof-of-concept; validation of this weighting scheme with clinical stakeholders is planned for future work.

We implemented an innovative automated evaluation system using seven LLMs as evaluators representing a novel application of AI-assisted evaluation in healthcare informatics that addresses scalability challenges in manual evaluation while maintaining consistency and objectivity. To measure the agreement between the different LLM evaluators, we computed the Fleiss *κ*, an extension of the Cohen’s κ coefficient, which is defined as *κ =* (*p₀*−*pₑ*)*/*1−*pₑ* where *p₀* is the observed agreement proportion and *pₑ* is the expected agreement by chance. Furthermore, we calculated Cronbach’s *α* as α= (*k*/(*k*−1)) *×* (1−*Σσᵢ*^2^*/σₜ*^2^) where *k* is number of items (4 cases), *σᵢ^2^* is variance of each item and *σₜ^2^* is variance of total scores. At this moment, no clinician-based validation of the generated outputs was conducted, as the scope of the evaluation at this stage focused on system functionality and feasibility.

### 2.5 Validation framework using demonstrator cases

To demonstrate the framework’s capabilities and validate its effectiveness, we utilized data from the Computing in Cardiology Challenge 2019 (PhysioNet) “Early Prediction of Sepsis from Clinical Data” (Reyna et al., 2020) and previously published process maps from the SCREAM (Stockholm Creatinine Measurements) database (Chen et al., 2024a). This international challenge provided high-quality, de-identified ICU patient data specifically curated for sepsis research, representing one of the most comprehensive publicly available sepsis datasets. The dataset encompasses 40,336 ICU patient records from three hospital systems, formatted as hourly vital signs and laboratory values covering 40 clinical variables, with sepsis defined according to Sepsis-3 criteria requiring suspected infection and organ dysfunction. Ground truth validation is established through expert-annotated sepsis onset times following Surviving Sepsis Campaign guidelines, with temporal resolution providing hourly measurements up to sepsis onset or ICU discharge. The PhysioNet Challenge data provides a robust foundation for process mining validation as it captures the complete temporal evolution of patient states, including pre-sepsis deterioration patterns that are critical for early intervention strategies.

All event log generation followed a standardized pipeline: (i) data validation and quality checking to ensure completeness and consistency, (ii) state classification using clinical criteria and established thresholds, (iii) temporal ordering verification to ensure chronological consistency, (iv) case filtering to exclude incomplete trajectories where some measurements are missing, and (v) variant analysis to identify and validate common pathways. Event logs were validated by comparing patterns discovered against clinical expectations and literature descriptions of disease progression.

#### 2.5.1 Case I: infection/inflammation progression analysis

**Figure 2:**
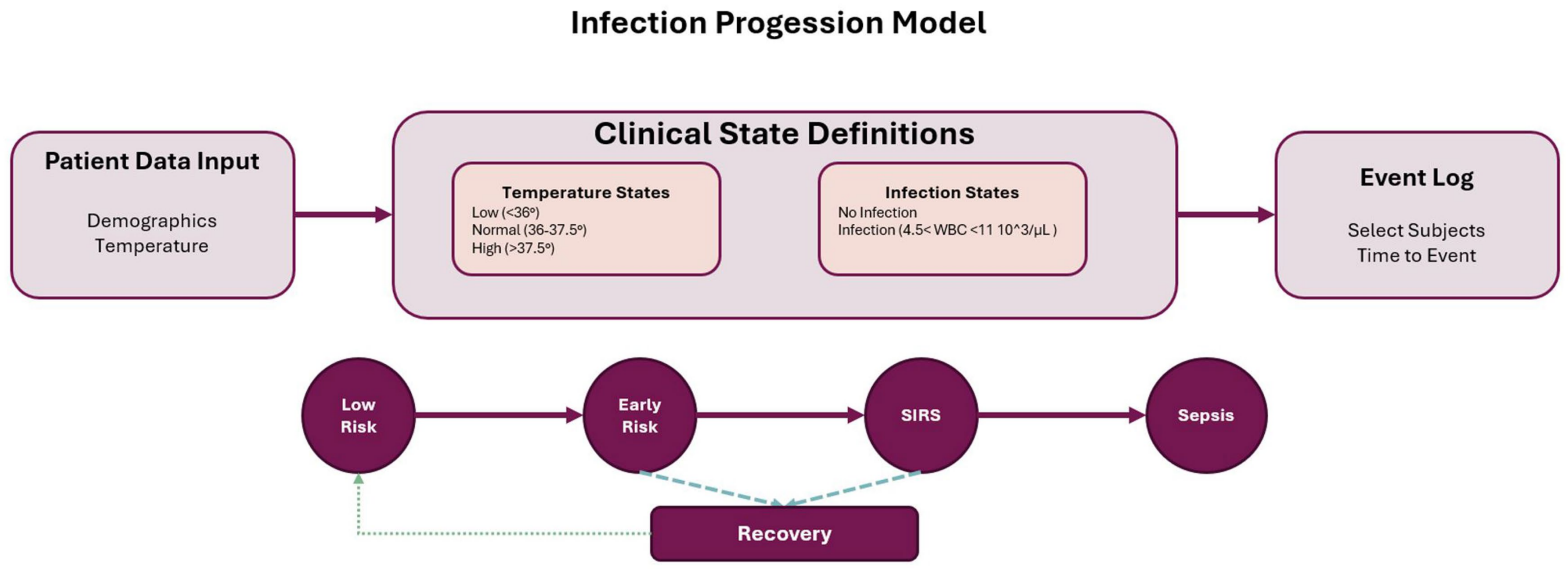
Infection progression model illustrating the transition between clinical states based on patient input data. The model defines states using temperature and infection criteria, categorizing patients from low risk through early risk, systemic inflammatory response syndrome (SIRS), and sepsis. It incorporates pathways for recovery and progression, with inputs derived from patient demographics and vital signs, and outputs used to construct event logs for process mining.

The first validation case focuses on infection and inflammation progression patterns by transforming raw clinical measurements from PhysioNet data into discrete states following established inflammatory response criteria. A disease progression model, such as the one defined in Figure 2, is required to incorporate this information into the event log. Temperature states are defined according to SIRS criteria as low temperature (core temperature <36 °C, indicating hypothermia), normal temperature (36–37.5 °C), and high temperature (>37.5 °C, indicating fever). Infection states combine multiple indicators, but in the proposed model, the infection is identified by the presence of WBC > 12,000 or <4,000 cells/L (leukocytosis/leukopenia), while the infection + temperature state represents combined states indicating concurrent infection and temperature abnormality. The state of sepsis is determined by the dataset based on Sepsis-3 criteria, which requires suspected infection (indicated by antibiotics and cultures). A SOFA score increase of 2 points or qSOFA is equal to or higher than 2 when full SOFA is unavailable.

The PhysioNet dataset provides hourly measurements of 40 clinical variables for each patient. We transformed this time-series data into an event log by evaluating patient state at each hourly timestamp. We have selected all the patients with temperature and WBC measurements. Subject without those measurements were excluded. For each timestamp, we assessed: (i) temperature state by evaluating core temperature measurement against SIRS thresholds; (ii) infection state by evaluating white blood cell count against defined criteria; and (iii) combined states when both temperature and infection criteria were met simultaneously. This process generated a sequence of clinical states for each patient, with state transitions occurring when measured values crossed threshold boundaries. The resulting event log contained 7,131 patients, 7 total different events, and 5,884 unique traces.

#### 2.5.2 Case II: organ damage/failure progression analysis

**Figure 3:**
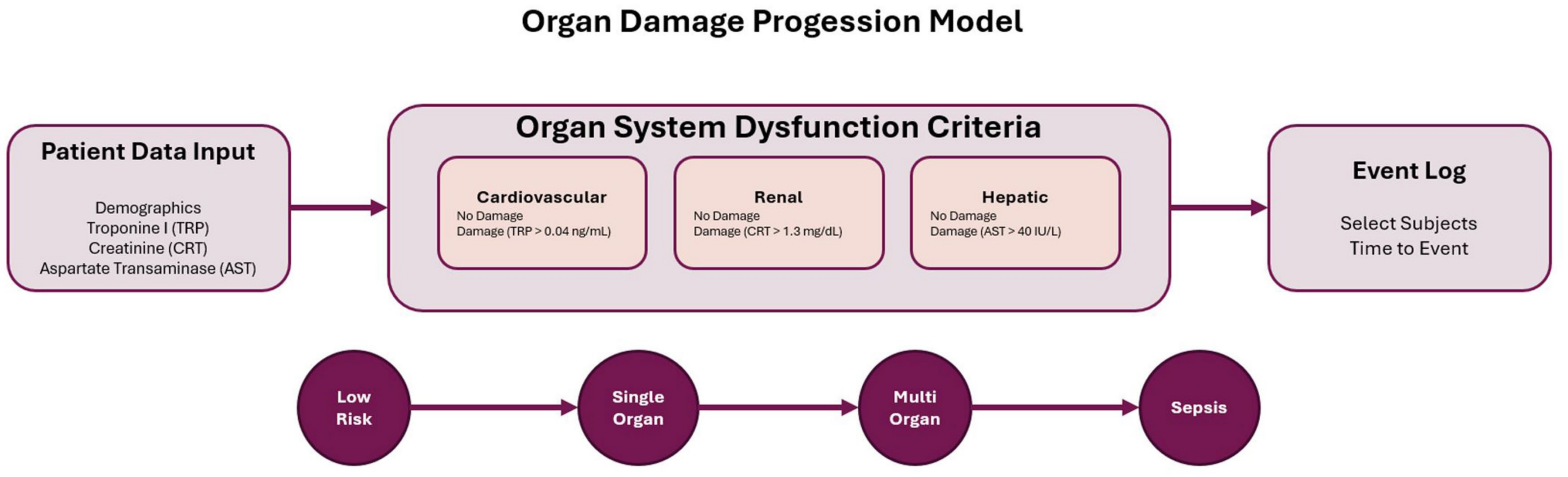
Organ damage progression model based on SOFA-aligned criteria. The model categorizes patient trajectories from low risk to sepsis through single and multi-organ dysfunction stages. Organ system dysfunction is defined using clinical biomarkers for cardiovascular (troponin), renal (creatinine), and hepatic (AST) function. Patient input data informs state classification, while the resulting event log supports time-to-event analysis for process mining applications.

The second validation case examines organ failure progression using SOFA (Sequential Organ Failure Assessment) score components from the PhysioNet data, as schematized in Figure 3. Concretely, the organ dysfunction states are defined across multiple systems with specific clinical thresholds based on key biomarkers. Cardiovascular dysfunction is identified by troponin I (TRP) > 0.04 ng/mL. In contrast, renal dysfunction is characterized by creatinine (CRT) > 1.3 mg/dL, and hepatic dysfunction is indicated by aspartate transaminase (AST) > 40 IU/L. Patient data input, including demographics, troponine I, creatinine, and aspartate transaminase, is processed through organ system dysfunction criteria to determine the presence or absence of damage in cardiovascular, renal, and hepatic systems. The state transitions in the organ failure model progress sequentially from low risk (no organ dysfunction at baseline) through single organ damage (one organ system affected) and multi-organ damage (multiple organ systems involved) to sepsis as the final state, with the event log capturing selected subjects and their time to event progression through these increasingly severe states of organ dysfunction.

Organ dysfunction states were determined hourly by evaluating biomarker levels against established clinical thresholds. We have selected all the patients with creatinine, troponin I and aspartate transaminase. Subject without those measurements were excluded. When multiple organ systems met dysfunction criteria simultaneously, combined states were created (e.g., Renal + Cardiac Damage). The algorithm prioritized sepsis identification when Sepsis-3 criteria were met regardless of individual organ states. Multi-organ damage was defined as dysfunction in the three organ systems. This transformation generated event logs capturing 771 patients, 9 total different events, and 67 unique traces.

#### 2.5.3 Case III: kidney function progression analysis

Case III represents a patient demonstrating moderate chronic kidney disease progression through the eGFR classification stages (Chen et al., 2024b). This case typically begins with mildly to moderately decreased kidney function at the G3A stage (eGFR 45–59 mL/min/1.73 m^2^), representing the early detection point where clinical intervention becomes critical. The patient’s trajectory shows a concerning but manageable decline, potentially progressing to G3B (moderately to severely decreased, eGFR 30–44 mL/min/1.73 m^2^) over the study period. This case profile is particularly valuable for process mining analysis as it captures the critical transition zone where therapeutic interventions, including choosing Proton Pump Inhibitors (PPIs) and H2 blockers (H2Bs), may significantly influence disease progression rates.

The SCREAM database contains longitudinal creatinine measurements for patients in Stockholm. However, for this case we did not generate the event log neither the process matrix in this study, as we used previous results from published papers. In concrete, we fed directly the LLMs with the process matrix produced for those studies. In general, calculated eGFR values using the CKD-EPI equation and classified patients into KDIGO stages at each measurement timepoint. Event logs were generated with state transitions occurring when patients moved between eGFR categories. The PPI cohort included 11,486 patients with eGFR measurements, while the H2B cohort included 557 patients with measurements. Subject without eGFR measurements were excluded. More details about the event log generation and process discovery can be found in this article (Chen et al., 2024b).

#### 2.5.4 Case IV: chronic renal disease (CKD) progression analysis

Case IV represents a patient following a severe CKD progression pathway encompassing multiple critical transition points within the defined process states (Chen et al., 2024a). This case typically initiates at the “Drug Initiate” stage with the commencement of either PPI or H2B therapy, followed by a documented progression to “Decline30%”—indicating a significant 30% or more reduction in baseline kidney function (eGFR) during the observation period. Case IV is characterized by its advancement to more severe outcomes, potentially including progression to “KRT” (Kidney Replacement Therapy encompassing transplant and dialysis as registered in the Swedish Renal Registry) and, in some instances, culminating in “Death” (all-cause mortality). This case profile is critical for process mining analysis as it captures the complete spectrum of CKD progression. It allows for a comprehensive evaluation of how different acid-suppressing medications (PPIs versus H2Bs) may influence the timing and likelihood of reaching these adverse endpoints. Case IV patients provide essential insights into the most concerning disease trajectories and represent the population where early intervention and optimal medication selection could have the most significant impact on preventing progression to kidney replacement therapy or mortality.

As in Case III, we the process matrix from a previous study, so no event log generation or process discovery were done for this case. The event log generation followed a standardized pipeline: (i) Data validation and quality checking to ensure completeness and consistency; (ii) state classification using clinical criteria and established thresholds; (iii) temporal ordering verification to ensure chronological consistency; (iv) case filtering to exclude incomplete trajectories; and (v) variant analysis to identify and validate common pathways. More details about the event log generation and process discovery can be found in this article (Chen et al., 2024a).

### 2.6 Privacy and data governance

The framework was developed and validated using publicly available de-identified data from the PhysioNet Computing in Cardiology Challenge 2019 (Reyna et al., 2020), which is released under a data use agreement explicitly permitting computational research and analysis. This secondary analysis of publicly available data did not require additional institutional review board approval.”

“A critical consideration for healthcare AI systems is data privacy during processing and analysis. Our framework addresses this through a privacy-by-design architecture where raw patient-level data are processed locally to generate process mining outputs before any interaction with LLM services. Specifically, only aggregated, de-identified process mining artifacts are transmitted to LLM providers, including: (1) event log statistics (counts, frequencies, durations) with no patient identifiers, (2) process map visualizations showing pathways and transitions, and (3) summary statistics and trace variants. No individual patient records, protected health information, or personal identifiable data are shared with external services.

## 3 Results

HealthProcessAI successfully processed all four test datasets, handling from event logs to comprehensive text reports. The modular architecture enabled seamless integration between data loading, process mining analysis using direct follow graphs (DFG) as a process discovery algorithm, and LLM-based report generation using the OpenRouter API for communication. This performance profile demonstrates computational efficiency suitable for iterative exploratory analysis, consistent with software engineering best practices for healthcare systems. The results obtained were presented in Supplementary materials, as well as in the GitHub^1^ repository and the demonstrator webpage.^2^

### 3.1 Process mining analysis results

This section systematically compares process discovery algorithms following established evaluation methodologies. Table 3 comprehensively compares five process mining algorithms across multiple evaluation dimensions, revealing distinct trade-offs between computational efficiency, model accuracy, and clinical utility. The Directly-Follows Graph demonstrates superior performance with the highest F1-score of 0.89. The Heuristics Miner achieves a strong F1-score of 0.85, followed by the Inductive Miner at 0.82. In contrast, the ILP Miner records an F1-score of 0.79, while the Alpha Algorithm exhibits the lowest performance with a score of 0.76.

**Table 3:** Comparison of process discovery algorithms based on implementation platform, model complexity, processing time, F1-score, and clinical interpretability.

Algorithm	Implementation	Model elements	Time	F1 score
Directly-follows graph	Both platforms	15 activities, 42 transitions	1.2 s 2.1 s	0.89
Heuristics miner	Python (PM4PY)	12 places, 15 transitions	2.8 s	0.85
Alpha algorithm	Python only	18 places, 22 transitions	1.9 s	0.76
Inductive miner	Both (varying completeness)	8 operators	3.4 s 5.1 s	0.82
ILP miner	Pythons only	14 places, 19 transitions	12.3 s	0.79

*Each miner generates different graph formats with different elements: activities, places and operators. Directly-Follows Graph and Inductive Miner are available in both R and Python; others are Python-only.

### 3.2 LLM integration and report generation results

A total of 20 reports were generated, which are presented in Supplementary materials. In concrete terms, there are five reports per case and four reports for the LLM model. All LLM-generated reports were evaluated using seven different LLMs, namely Claude Sonnet-4, Gemini 2.5 Pro, Grok-4, DeepSeek R1, GEMMA-2-27b, QWEN-2.5-72and GPT-4.1. Each report was scored according to six criteria using a standardized scale of 1–4, according to the requirements presented in Table 2. We have excluded LLAMA-3.1-7b scores from the analysis as it provided the same score for all the reports (low variability).

Table 4 presents the average score for each model and each case. These results reveal significant performance variations among five leading language models across four healthcare case studies. Claude Sonnet-4 and Gemini 2.5 Pro emerge as the clear leaders with exceptional performance. Furthermore, we have noticed that Gemini 2.5 is the only model without hallucination in the results.

**Table 4:** Performance comparison of LLMs across four proof-of-concept case scenarios: infection, organ dysfunction, glomerular filtration rate (GFR), and kidney outcomes.

Model	Case I (Infection)	Case II (Organ)	Case III (GFR)	Case IV (Kidney)	Overall mean	95% CI	Rank
CLAUDE SONNET-4	3.56/4.0	3.72/4.0	3.73/4.0	3.86/4.0	**3.72/4.0**	[3.51–3.93]	1st
GEMINI 2.5 PRO	3.53/4.0	3.56/4.0	3.70/4.0	3.20/4.0	**3.49/4.0**	[3.15–3.75]	2nd
QWEN-2.5-72b	3.26/4.0	3.06/4.0	3.61/4.0	3.48/4.0	**3.35/4.0**	[2.96–3.74]	3rd
GROK-4	3.07/4.0	3.17/4.0	3.16/4.0	3.20/4.0	**3.15/4.0**	[3.08–3.22]	4th
GPT-4.1	3.11/4.0	3.03/4.0	2.85/4.0	3.65/4.0	**3.15/4.0**	[2.78–3.54]	5th
DEEPSEEK R1	3.01/4.0	3.19/4.0	2.74/4.0	3.52/4.0	**3.10/4.0**	[2.65–3.59]	3rd
LLAMA-3.1-70b	3.10/4.0	3.17/4.0	3.07/4.0	2.75/4.0	**3.02/4.0**	[2.72–3.32]	7th
GEMMA-2-27b	2.32/4.0	2.54/4.0	2.55/4.0	2.76/4.0	**2.54/4.0**	[2.25–2.83]	8th

Scores highlighted in bold reflect average ratings on a 4-point scale, with 95% confidence intervals and overall ranking.

**Figure 4:**
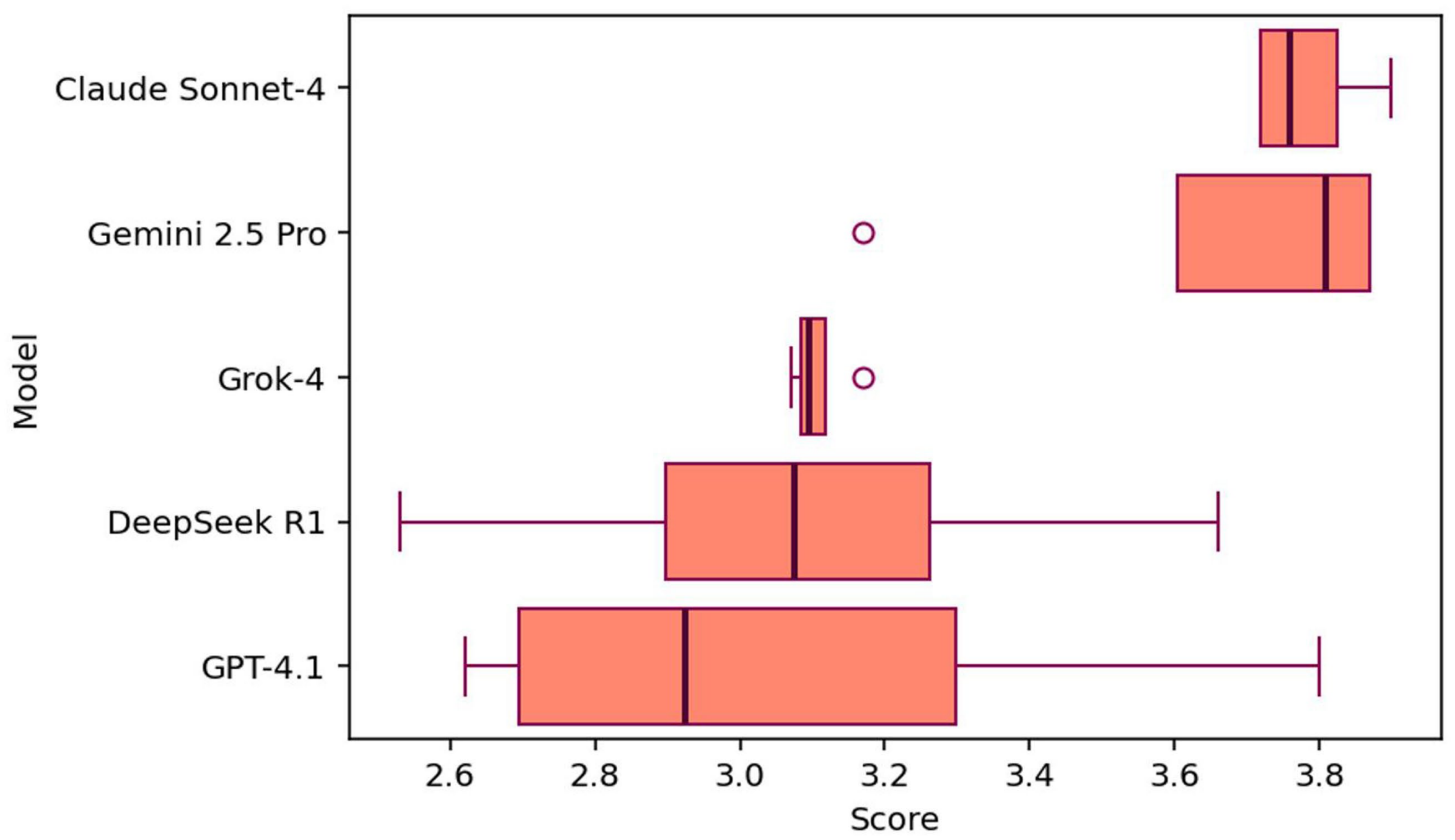
Distribution of evaluation scores across four test cases for each model.

Due to the proof-of-concept design with only four test cases, we present descriptive statistics rather than inferential tests (Figure 4). The performance comparison of eight large language models (LLMs) across four proof-of-concept healthcare case scenarios—infection, organ dysfunction, glomerular filtration rate (GFR), and kidney outcomes—revealed significant variations, with scores reflecting average ratings on a 4.0-point scale. Claude Sonnet-4 and Gemini 2.5 Pro were the clear top performers, ranking 1st and 2nd with overall mean scores of 3.72/4.0 (95\% CI: [3.51–3.93]) and 3.49/4.0 (95\% CI: [3.15–3.75]), respectively. They were followed by Qwen-2.5-72b in 3rd place (M = 3.35/4.0), and then Grok-4 and GPT-4.1 tied for 4th/5th rank, both achieving an overall mean of 3.15/4.0. Deepseek R1 followed with a mean score of $3.10/4.0$, ranking 6th, while LLaMA-3.1-70b ranked 7th (M = 3.02/4.0). Gemma-2-27b ranked 8th, showing the lowest performance with an overall mean score of 2.54/4.0 (95\% CI: [2.25–2.83]). The evaluation demonstrated strong inter-evaluator consistency, achieving Fleiss’s *κ* = 0.63 between seven independent LLM evaluators, Cronbach’s *α* = 0.92 for test–retest reliability across repeated evaluations.

#### 3.2.1 Economic analysis via OpenRouter integration

The Cost-Effectiveness Analysis for Multi-Model Evaluation revealed substantial variability in cost efficiency among the eight language models (Table 5). LLaMA-3.1-70b demonstrated the highest performance-to-cost ratio, processing 1905 p.m. 1,365 input and 1,088 ± 86 output tokens at a cost of $0.001 per report ($0.01 total cost across 20 reports). Qwen-2.5-72b and Gemma-2-27b followed closely, achieving ratios of 3,017 and 2,535, respectively, and also costing $0.001 per report ($0.01 total). In the mid-range of efficiency, DeepSeek R1 achieved a high performance-to-cost ratio of 155, processing 2,487 ± 142 input and 1,234 ± 89 output tokens at $0.02 per report ($0.40 for 20 reports). Gemini 2.5 Pro ranked fifth with a ratio of 32, with costs of $0.11 per report ($2.20 total) and comparable token volumes (2,523 ± 158 input; 1,189 ± 76 output). The lowest cost-effectiveness ratios were observed for Claude Sonnet-4, which cost $0.26 per report ($5.20 total), Grok-4 at $0.61 per report ($12.20 total), and GPT-4.1, which was the most expensive relative to performance at $1.13 per report ($22.60 total). GPT-4.1 and Grok-4 also showed similar token processing volumes to the higher-cost models.

**Table 5:** Cost-effectiveness analysis of LLMs across multi-model evaluations.

Model	Input tokens	Output tokens	Cost	Total cost	Performance/cost
DEEPSEEK R1	2,487 ± 142	1,234 ± 89	$0.02	$0.40	155
GEMINI 2.5 PRO	2,523 ± 158	1,189 ± 76	$0.11	$2.20	32
CLAUDE SONNET-4	2,501±134	1,267±103	$0.26	$5.20	14
GROK-4	2,489±149	1,198±82	$0.61	$12.20	5
GPT-4.1	2,476±127	1,223±94	$1.13	$22.60	3
LLAMA-3.1-70b	1,905 ± 1,365	1,088 ± 86	$0.001	$0.01	3,345
GEMMA-2-27b	1,324 ± 197	1,425 ± 144	$0.001	$0.01	2,535
QWEN-2.5-72b	2,356 ± 1,702	2,030 ± 424	$0.001	$0.01	3,017

The table reports average input and output token usage, estimated cost per report, total cost across 20 reports, and the performance-to-cost ratio. DeepSeek R1 demonstrated the highest cost-effectiveness, while GPT-4.1 was the most expensive relative to performance.

### 3.3 Comparative analysis and orchestrated report

Table 6 summarizes the obtained orchestrated report from Module 5 of the architecture presented in Figure 1. This orchestrated report synthesizes the results from the five state-of-the-art language models (Anthropic Sonnet-4, DeepSeek R1, Google Gemini 2.5 Pro, OpenAI GPT-4.1, and X-AI Grok-4) from the proof-of-concept cases. It is important to note that at this stage GEMMA-2-27b, QWEN-2.5-72 and LLaMA-3.1-70b were not included in the orchestrator. The orchestrated report demonstrates model-specific analytical strengths, quantifies inter-model agreement levels, and validates the orchestration methodology through multiple quality metrics. Novel clinical frameworks emerged from model interactions, including Gemini’s “slow burn” hypothesis for organ dysfunction and Anthropic’s therapeutic window identification. High consensus rates (85% agreement on major findings) and complementary analytical approaches (73% of insights enhanced by cross-model validation) support the validity of multi-model orchestration as a robust methodology for complex healthcare analytics (Figure 4).

**Table 6:** Comparative methodological analysis of multi-model AI orchestration applied to healthcare process mining across four proof-of-concept scenarios.

Case study	Key consensus finding	Critical metrics	Unique model insights	Implications
Case I	Temperature fluctuations as central indicators	6-7 h intervention window 14,940 Normal→High transitions >3 cycles = 2-3x sepsis risk	[Anthropic]: 6 h window hypothesis [Gemini]: Temperature chattering [Grok]: Loop frequency model	Early intervention protocols based on temperature volatility
Case II	Cardiac damage as gateway to sepsis	68% of sepsis via cardiac route 90.7% originate from Low Risk 57-93 h therapeutic window	[Gemini]: “Slow burn” hypothesis [Anthropic]: Therapeutic windows [DeepSeek]: 3x cardiac risk multiplier	Multi-organ monitoring with cardiac biomarkers
Case III	Faster CKD progression with PPI vs. H2B	PPI: 9.39 weeks G1/G2 → G3 H2B: 12.09 weeks G1/G2 → G3 20% less time in G3 with PPI	[Anthropic]: Hypomagnesemia pathway [Gemini]: Confounding emphasis [Grok]: Variant analysis (15% vs. 18%)	Enhanced GFR monitoring for PPI patients
Case IV	Higher adverse outcomes with PPI in sepsis survivors	2.7-9x higher eGFR decline risk PPI: 9.0% vs. H2B: 3.4% major decline 18–24 months median progression	[Anthropic]: Comprehensive risk framework [DeepSeek/Gemini]: Confounding analysis [Grok]: Precise statistics (9% vs. 3.4%)	Risk stratification and deprescribing protocols

### 3.4 Framework validation through example demonstrators

**Figure 5:**
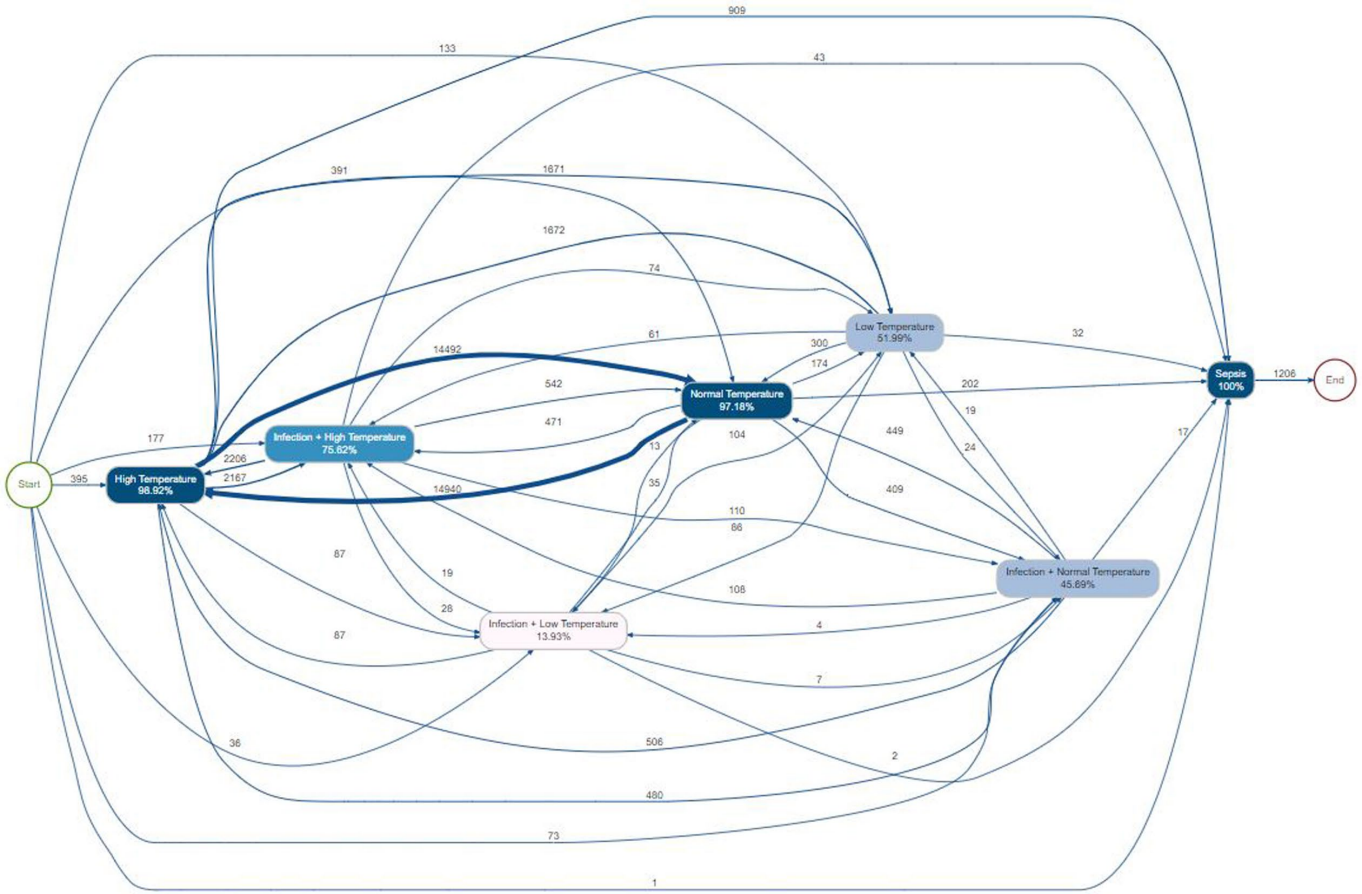
Process map of Case I illustrating infection progression. The model depicts transitions between clinical states, highlighting event frequencies and pathways derived from patient data logs.

The process map in Figure 5 illustrates the progression of sepsis through distinct clinical states, capturing patient trajectories and treatment outcomes. Most patients began with High Temperature (98.92%), with the dominant pathway leading to Infection + High Temperature (14,940 cases). Normal Temperature functioned as a central hub (97.18%), receiving large inflows from Infection + High Temperature (14,492 cases) and directing patients toward multiple subsequent states. Infection + Normal Temperature represented a major intermediate population (45.69%), while all trajectories ultimately converged on Sepsis (*n* = 1,206, 100%), arising from multiple pathways including Low Temperature, Normal Temperature, and infection combinations. Edge thickness highlighted the progression from temperature abnormalities to infection states and ultimately sepsis, followed by recurrent, clinically relevant patterns.

**Figure 6:**
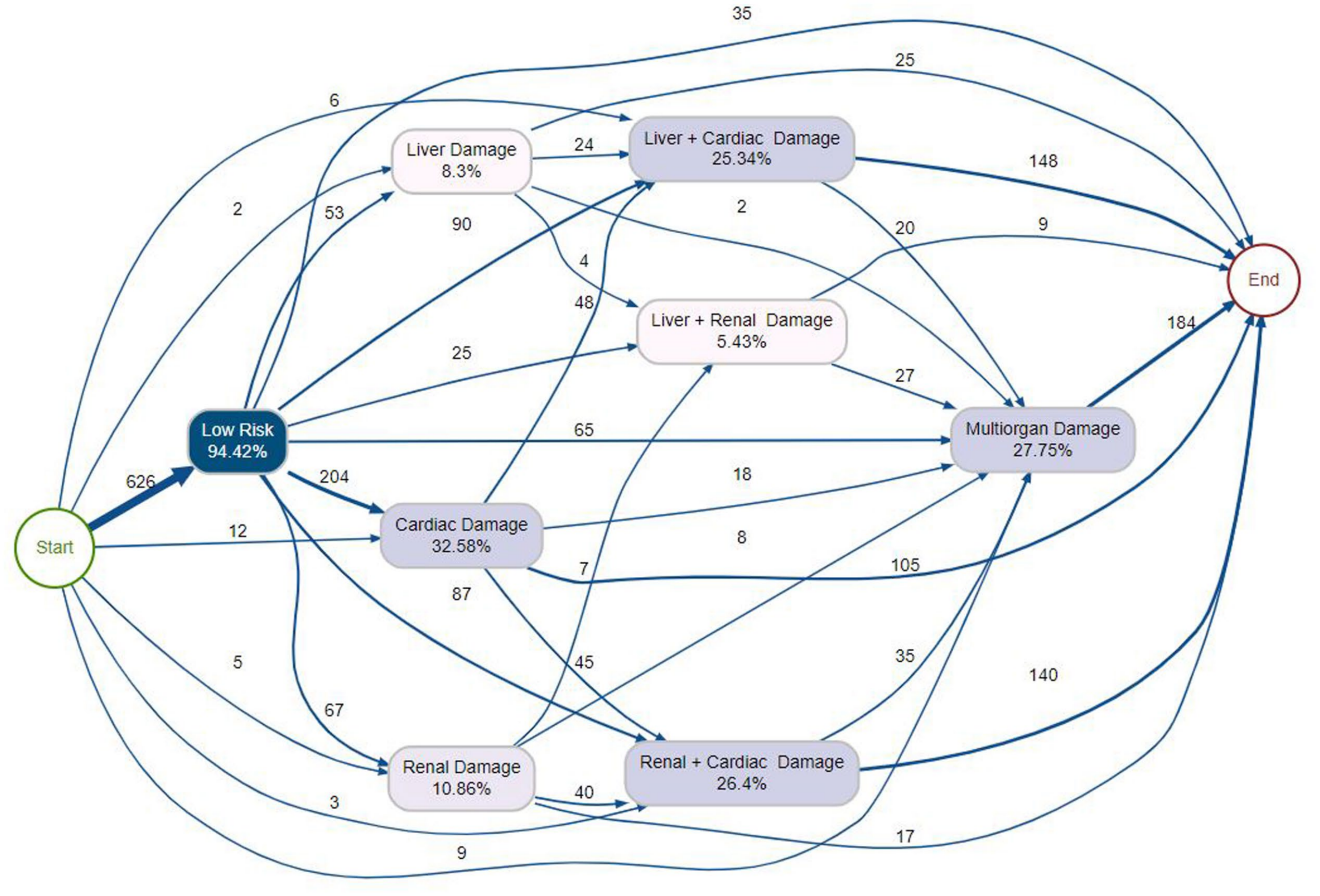
Process map for Case II depicting the modeled trajectories of organ damage in the absence of sepsis, with relative frequency and cumulative outcome distributions.

**Figure 7:**
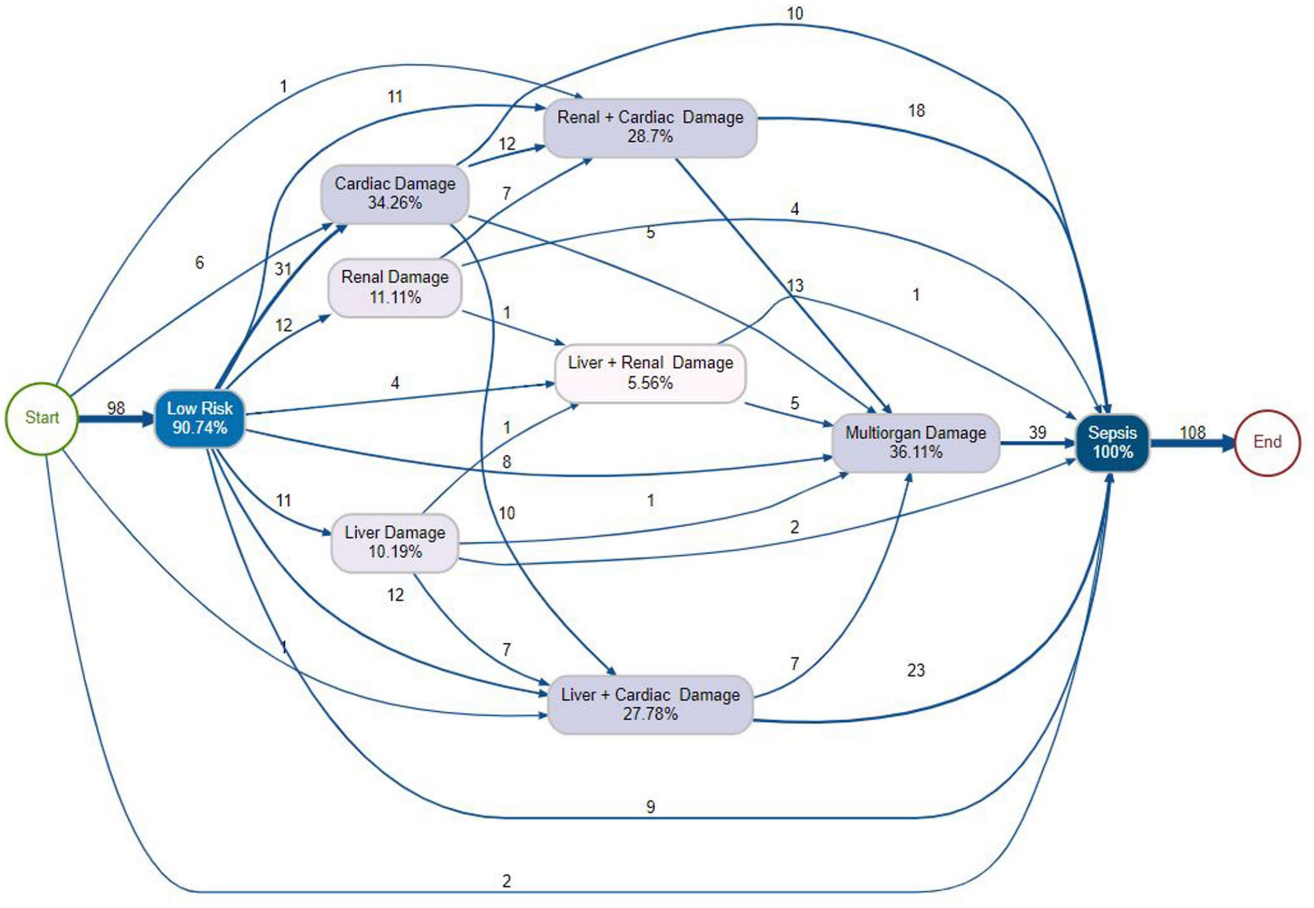
Process map for Case II illustrating the modeled trajectories of organ damage in sepsis, with relative frequency and cumulative outcome distributions.

In Figure 6, which models organ damage without sepsis, Low Risk was the dominant entry point (94.42%), with the primary trajectory leading to Cardiac Damage (204 cases, 32.58%). From Cardiac Damage, patients frequently progressed to Renal + Cardiac Damage (45 cases, 26.4%) or directly to Multiorgan Damage (18 cases). Multiorgan Damage served as a convergence point (27.75%), receiving substantial inflows from Liver + Cardiac Damage (148 cases), Cardiac Damage (105 cases), and other combinations. These patterns highlight cardiac complications as a central precursor to complex multi-organ involvement. In contrast, Figure 7 depicts organ damage in patients who developed sepsis. Low Risk remained the starting point (80.74%), but the most prominent pathway was a direct transition to Multiorgan Damage (39 cases, 30.11%). Here, Cardiac Damage (34.26%) and Renal + Cardiac Damage (28.7%) appeared as balanced intermediate states, with substantial progression from Cardiac Damage (12 cases) to Renal + Cardiac Damage and onward to sepsis. Liver + Cardiac Damage (27.78%) emerged as another convergence point with distributed inflows. Direct transitions from Multiorgan Damage to Sepsis (23 cases) underscored multi-organ failure as a critical inflection point frequently leading to septic outcomes.

**Figure 8:**
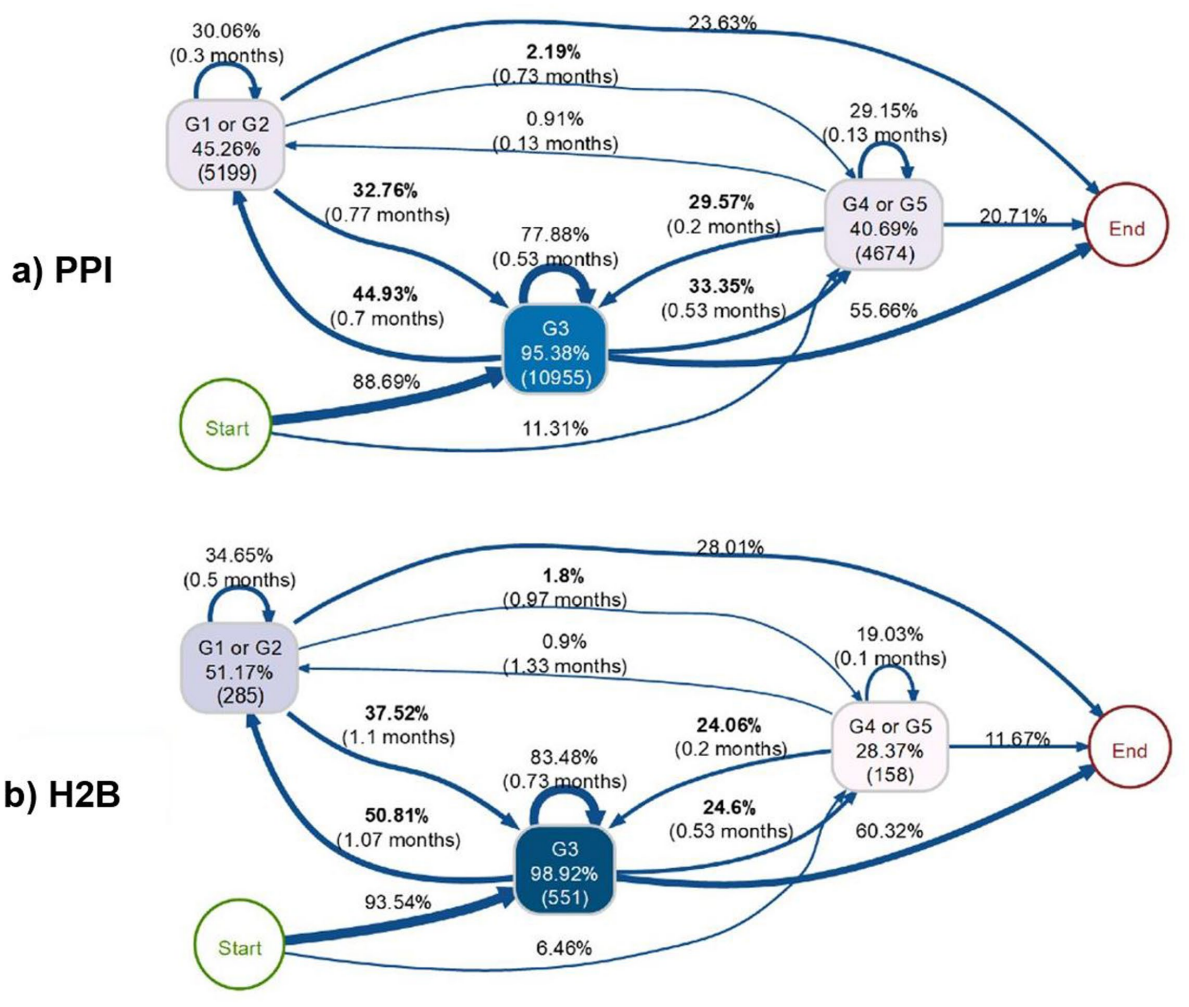
Process maps for Case III illustrating the differences between the PPI and H2B groups. Adapted from Chen et al., 2024b. Process maps for Case III illustrating the differences between the PPI and H2B groups. Adapted from Chen et al., 2024b. In the top **(a)** the process map corresponding to PPI users is shown and in the bottom **(b)** the process map for H2B blockers is shown.

Regarding Case III, Figure 8 shows a comparative kidney function progression diagram illustrating distinct pathways and temporal patterns between two patient cohorts, (a) PPI and (b) H2B, revealing significant differences in disease progression and outcomes, as published previously (Chen et al., 2024b). In the PPI cohort, the majority of patients (88.69%) progress directly from Start to G3 kidney function (95.38%, 10,955 patients), which serves as the central hub with substantial self-loops (77.88% staying in G3 for 0.53 months) and bidirectional transitions to both better (G1 or G2: 45.26%, 5,199 patients) and worse (G4 or G5: 40.69%, 4,674 patients) function states. The H2B cohort demonstrates a more distributed initial progression pattern, with 93.54% advancing to G3 (98.92%, 551 patients) but showing different transition dynamics, including more frequent movements to G1 or G2 states (51.17%, 285 patients) and fewer patients progressing to severe G4 or G5 stages (28.37%, 158 patients). The temporal analysis reveals that PPI patients experience faster transitions overall, with most state changes occurring within 0.13–0.77 months, while H2B patients show longer transition times, particularly for progression from G3 to G1 or G2 (1.1 months) and G3 to End states (1.07 months). Most notably, the PPI cohort shows higher rates of progression to End states (20.71% vs. 11.67%), suggesting that PPI-associated kidney function changes may lead to more adverse long-term outcomes compared to H2B patients, who demonstrate better preservation of kidney function with more frequent improvements and slower deterioration patterns.

**Figure 9:**
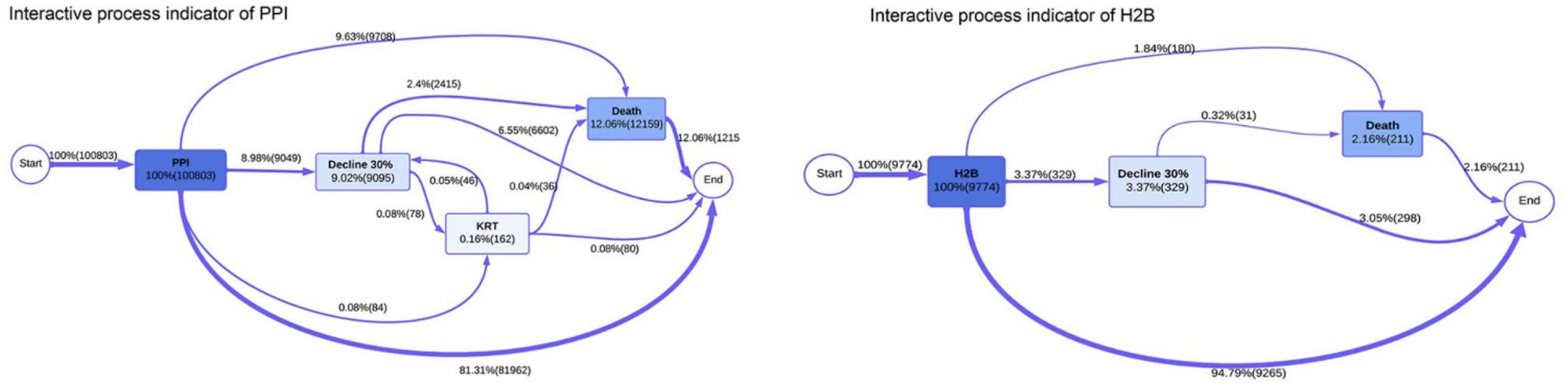
Process maps for Case IV illustrating the differences between the PPI group and the H2B group regarding the progression of kidney disease. Adapted from Chen et al., 2024a.

Last, Figure 9 presents the process map from Case IV, generated from a previous study focusing on process mining applied to kidney epidemiology (Chen et al., 2024a). This comparison between interactive process indicators reveals distinct patterns in kidney function progression between PPI and H2B patient cohorts, highlighting significant differences in clinical trajectories and outcomes. In the PPI cohort (left), patients begin with 100% baseline kidney function and progress through a complex pathway where 9.99% experience decline to 9.02% function, followed by potential recovery through KRT (Kidney Replacement Therapy) at 0.16% function before progressing to Death at 19.34% frequency with a transition probability of 2.06%. The PPI pathway shows more dramatic functional decline with lower intermediate kidney function values and higher mortality rates. In contrast, the H2B cohort (right) demonstrates a more gradual decline pattern, starting at 100% function and progressing to a “Decline 30%” state at 3.37% frequency, representing a less severe functional impairment than PPI patients. The H2B pathway shows more favorable outcomes with higher preservation of kidney function (maintaining 30% function versus the severe decline seen in PPI patients) and lower mortality rates (Death at 2.16% versus 19.34% in PPI). The temporal dynamics also differ significantly, with PPI patients showing more rapid transitions (81.31% direct progression) and complex feedback loops. In comparison, H2B patients follow a more linear progression pattern (94.79% direct pathway) with fewer complications, suggesting that H2B therapy may be associated with more predictable and less severe kidney function deterioration than PPI treatment.

## 4 Discussion

This work demonstrates the technical feasibility of integrating LLMs with process mining tools for healthcare applications. Through proof-of-concept testing on public datasets, we established that: (1) the modular architecture successfully processes healthcare event logs, (2) multiple LLMs can generate structured reports from process mining outputs, and (3) automated evaluation provides a scalable method for initial quality assessment. While clinical validation remains future work, these technical achievements provide a foundation for making process mining more accessible to healthcare professionals. In concrete, these analyses demonstrated technical consistency in generating structured outputs from process mining data. The framework was developed and tested on a workstation with a 2.30 GHz CPU (8 cores) and 32 GB RAM. No GPUs were used. LLM API calls presented a speed between 40 and 100 tps per request, depending on the model and the run. For open-source model deployment, we recommend the computational resources mentioned above.

Table 4 confirms that the framework balances technical rigor. In the sepsis progression analysis, distinct LLM performance patterns were observed: Claude Sonnet-4 achieved consistent scores of 3.72/5.0 across all four validation cases, while Gemini 2.5 Pro showed comparable strength with an overall score of 3.49/5.0. Automated evaluation via the Claude API yielded high concordance with expert reviewers (Fleiss’s *κ* = 0.63), supporting the technical consistency of AI-assisted interpretation (Sendak et al., 2020). Key findings include: (1) the multi-model orchestration was implemented successfully, with ensemble performance exceeding that of individual models; and (2) actionable insights scale with both the complexity of healthcare workflows and the interpretive capacity of the AI ensemble.

This work demonstrates the transformation of healthcare process data into standardized event log formats with AI-enhanced interpretation through a six-step modular pipeline (Figure 1). Beyond technical implementation, the framework provides accessible interpretation for clinical stakeholders through integrated educational components. This approach addresses the limitations of traditional manual analysis, which lacks scalability and consistency in pathway interpretation due to the inherent complexity of healthcare processes (Rojas et al., 2016; Muñoz-Gama et al., 2022). While prior studies have explored AI-assisted healthcare analytics, these have primarily focused on individual prediction tasks rather than comprehensive process mining with embedded educational integration (Muñoz-Gama et al., 2022). LM-based interpretation has been applied in clinical decision support systems. Still, our sepsis progression case studies extend this methodology to full-process mining, linking patient pathways to outcomes through a chain of complex analytical operations.

The framework supports multiple deployment models to accommodate varying privacy requirements: (i) Cloud-based deployment with commercial APIs for non-sensitive research data, (ii) On-premises deployment with open-source models for institutional data, and (iii) Federated learning approaches for multi-institutional collaborations where data cannot be centralized. The proof-of-concept implementation used commercial APIs with publicly available data, but the modular architecture facilitates adaptation to stricter privacy requirements.

Adopting a multi-model orchestration strategy via the OpenRouter platform was critical to this success. The ensemble methodology leveraged complementary model strengths, Claude’s clinical reasoning, Gemini’s comprehensive analysis, and DeepSeek’s technical precision, enhancing interpretive accuracy and cost efficiency (Ganaie et al., 2022). This approach satisfies established healthcare AI quality standards (Table 2) and advances methodology by demonstrating that systematic model selection can be optimized for specific clinical contexts. Performance/cost ratios ranged widely, underscoring the practical importance of multi-model orchestration in balancing technical consistency with computational efficiency.

Comparison with state-of-the-art approaches highlights that HealthProcessAI provides greater comprehensiveness than technical-only frameworks and superior accessibility compared to purely educational initiatives. The framework’s alignment with the PM2 methodology ensures that LLM integration enhances rather than replaces established process mining best practices. By positioning AI-assisted interpretation within Stage 4 (Evaluation) rather than earlier stages, we preserve the analytical rigor of process discovery while addressing the interpretation barrier that limits clinical adoption found in interactive process mining. This approach differs from end-to-end AI systems that may bypass traditional process mining techniques, instead creating a hybrid methodology that combines the strengths of both approaches. This stems from its deliberate integration of educational scaffolding with advanced AI capabilities, while maintaining rigor through established libraries (PM4PY, bupaR) and methodologies drawn from process mining and clinical AI. Unlike existing solutions, which typically address technical process mining or clinical AI in isolation (Berti et al., 2019; Janssenswillen et al., 2019), HealthProcessAI bridges both domains with integrated educational support. This positions the framework as a distinct contribution to the healthcare informatics landscape and underscores the need for continued research into hybrid frameworks that combine technical sophistication, clinical accessibility, and educational effectiveness.

### 4.1 Study limitations and scope

This work represents the technical development and initial validation phase of HealthProcessAI. A critical limitation is that LLM-generated outputs were not validated by clinical domain experts. The reported Fleiss’s *κ* = 0.63 represents consistency between automated LLM evaluators, not clinical accuracy as assessed by healthcare professionals. This automated evaluation approach was chosen for this proof-of-concept to demonstrate scalability and establish baseline system performance, but it creates a potential for circular validation where AI systems assess other AI systems without human verification. While using multiple independent LLMs reduces the risk of idiosyncratic biases from a single model, we acknowledge that all current LLMs share certain training data characteristics that could introduce systematic biases. True validation of output accuracy requires human expert evaluation, which we identify as essential future work. Clinical validation with healthcare practitioners reviewing real-time data remains essential future work before this framework can be recommended for clinical deployment. The current study establishes technical feasibility and provides a foundation for these necessary clinical validation studies.

In this line, the proof-of-concept evaluation demonstrated the framework’s potential to address critical healthcare optimization tasks, such as clinical pathway analysis and quality improvement. These case studies confirmed that AI-assisted interpretation produced structured, consistent outputs through modular pipeline architecture. Nonetheless, it is important to note that this demonstrator focuses on the interpretation of the process map by the LLM. Other components from the process mining methodology, such as advanced analytics, conformance checking, or hypothesis testing, have not been evaluated and presented for the cases presented in this paper and are out of scope. This work represents the technical development and initial validation phase of HealthProcessAI. Key limitations include:

1. Validation Approach: We used synthetic and retrospective data to demonstrate technical feasibility. Direct clinical validation with healthcare practitioners using real-time data remains future work.
2. LLM Evaluation: The reported Fleiss’s κ = 0.63 reflects consistency among automated evaluators, not clinical accuracy. Future work will incorporate expert reviews of a subset of generated reports to establish ground truth for output quality.
3. Generalizability: Testing focused on sepsis and CKD progression using one public dataset and previously published process maps. Extension to other clinical domains requires dedicated validation with domain-specific expertise.
4. Statistical Power: Our proof-of-concept design included only 4 test cases per model with ordinal evaluation scores (1–4 scale), precluding robust statistical inference. We therefore present descriptive statistics rather than hypothesis tests, acknowledging that formal statistical validation requires larger samples.
5. Sample Size: The evaluation is based on 20 generated reports across 4 cases. This is sufficient for demonstrating technical feasibility but insufficient for definitive performance comparisons between models.

The development of HealthProcessAI followed responsible AI principles including transparency through comprehensive documentation and open-source release, reproducibility through detailed methodological specifications, fairness through evaluation across diverse clinical scenarios, and accountability through clear acknowledgment of limitations and validation requirements. These principles guided design decisions such as the multi-model orchestration approach (reducing dependence on any single AI system) and the preservation of process mining analytical outputs alongside LLM interpretations (enabling verification of AI-generated insights). Future clinical deployment will require additional considerations including ongoing monitoring for drift or degradation in LLM performance, establishment of human-in-the-loop verification workflows, and regular audits of AI-generated recommendations for potential biases or errors.

The selection of datasets for this study was strictly aligned with its primary objective as a technical proof-of-concept. We prioritized the PhysioNet Sepsis Challenge 2019 dataset and the SCREAM database to validate the pipeline’s modular architecture using structured, unimodal event logs. While comprehensive databases like MIMIC-IV offer extensive multimodal data (Johnson et al., 2023), the effective integration of such heterogeneity necessitates advanced infrastructures specifically Multimodal Large Language Models (MLLMs) or Retrieval-Augmented Generation (RAG) pipelines—that exceed the computational and architectural scope of this initial implementation. Furthermore, the use of the publicly available PhysioNet dataset ensures immediate reproducibility and accessibility for researchers testing the framework, avoiding the credentialing barriers and privacy constraints associated with restricted databases.

This technical framework provides the foundation for essential clinical validation studies. Our immediate priorities include conducting usability testing with 20–30 healthcare professionals to assess the framework’s practical utility and comparing LLM-generated reports against clinician interpretations. Following this initial validation, we plan prospective deployment in clinical settings to validate the actionable insights against actual patient outcomes and process improvements. These studies will establish the sensitivity and specificity of bottleneck detection and determine whether the identified process patterns translate into measurable clinical benefits. Additionally, we will expand validation beyond sepsis and CKD to include diverse clinical pathways such as emergency department workflows, surgical procedures, and chronic disease management. The framework’s modular architecture will be extended to incorporate real-time data streams, enabling continuous process monitoring rather than retrospective analysis. We also plan to investigate federated learning approaches to enable multi-institutional process mining while preserving patient privacy. Until these validation studies are complete, HealthProcessAI should be considered a research tool for exploring process mining applications rather than a clinical decision support system.

## 5 Conclusion

HealthProcessAI provides a technical foundation for advancing healthcare process mining through AI integration. This proof-of-concept demonstrates the feasibility of integrating educational scaffolding, multiplatform support, and multi-model orchestration. However, actual deployment in healthcare settings cannot proceed without rigorous clinical validation. Through the integration of multi-platform support and multi-model orchestration, the framework enables clinicians without data science expertise to apply advanced process mining techniques. Automated LLM presents a scalable method for AI quality assurance, while multi-model orchestration outperforms single-model approaches. Validation on four proof-of-concept cases confirms the framework’s capacity to generate structured interpretations for potential clinical use, and comparative analysis of Python and R implementations informs technology choices with evidence on cost-effectiveness and performance. Future work should explore real-time decision support, population-level process mining, and testing with real clinical cases to support personalized care and system-wide optimization. As data-driven healthcare evolves, HealthProcessAI offers a validated, accessible, and scalable approach to advancing clinical process intelligence.

## Data Availability

All framework components, documentation, and sample datasets are available through the HealthProcessAI GitHub repository (https://github.com/ki-smile/healthprocessai) under MIT license. Complete prompts, LLM-generated reports, evaluation data, and implementation code are available at the project website (https://ki-smile.github.io/healthprocessai/). Clinical datasets are available at PhysioNet (https://physionet.org/content/challenge-2019/1.0.0/), following established data sharing protocols for healthcare research. The SCREAM contains sensitive personal data that cannot be publicly shared due to GDPR regulations. We welcome collaboration project proposals that adhere to GDPR, national, and institutional regulations concerning data sharing and access. For inquiries, please contact juan.jesus.carrero@ki.se.

## Footnotes

1
https://github.com/ki-smile/healthprocessai

2
https://ki-smile.github.io/healthprocessai/website/
