# Supplementary material for: HealthProcessAI: a technical framework and proof-of-concept for LLM-enhanced healthcare process mining
Source: Front Artif Intell. 2026 Jan 30;9:1716819. doi: 10.3389/frai.2026.1716819 (PMC12901364; doi:10.3389/frai.2026.1716819)
Supplement: Supplementary file 1 [file Data_Sheet_1.ZIP › Supplementary Materials/Table S1.docx]

**Supplementary Table 1**

| **Table** | **Description** |
| --- | --- |
| Table S1 | List of supplementary tables for this article |
| Table S2 | Prompt for *Case I* |
| Table S3 | Prompt for *Case I* |
| Table S4 | Prompt for *Case II* |
| Table S5 | Prompt for *Case III* |
| Table S6 | Prompt for evaluating the reports (Source markdown) |
| Table S7 | Report generated by Claude Sonnet-4 for *Case I* (Source markdown) |
| Table S8 | Report generated by Gemini 2.5 Pro for *Case I* (Source markdown) |
| Table S9 | Report generated by DeepSeek R1 for *Case I* (Source markdown) |
| Table S10 | Report generated by Grok-4 for *Case I* (Source markdown) |
| Table S11 | Report generated by GPT-4.1 for *Case I* (Source markdown) |
| Table S12 | Report generated by Qwen-2.5-72b for *Case I* (Source markdown) |
| Table S13 | Report generated by Gemma-2-27b for *Case I* (Source markdown) |
| Table S14 | Report generated by Llama-3.1-70b for *Case I* (Source markdown) |
| Table S15 | Report generated by Claude Sonnet-4 for *Case II* (Source markdown) |
| Table S16 | Report generated by Gemini 2.5 Pro for *Case II* (Source markdown) |
| Table S17 | Report generated by DeepSeek R1 for *Case II* (Source markdown) |
| Table S18 | Report generated by Grok-4 for *Case II* (Source markdown) |
| Table S19 | Report generated by GPT-4.1 for *Case II* (Source markdown) |
| Table S20 | Report generated by Qwen-2.5-72b for *Case II* (Source markdown) |
| Table S21 | Report generated by Gemma-2-27b for *Case II* (Source markdown) |
| Table S22 | Report generated by Llama-3.1-70b for *Case II* (Source markdown) |
| Table S23 | Report generated by Claude Sonnet-4 for *Case III* (Source markdown) |
| Table S24 | Report generated by Gemini 2.5 Pro for *Case III* (Source markdown) |
| Table S25 | Report generated by DeepSeek R1 for *Case III* (Source markdown) |
| Table S26 | Report generated by Grok-4 for *Case III* (Source markdown) |
| Table S28 | Report generated by GPT-4.1 for *Case III* (Source markdown) |
| Table S29 | Report generated by Qwen-2.5-72b for *Case III* (Source markdown) |
| Table S30 | Report generated by Gemma-2-27b for *Case III* (Source markdown) |
| Table S31 | Report generated by Llama-3.1-70b for *Case III* (Source markdown) |
| Table S32 | Report generated by Claude Sonnet-4 for *Case I* (Source markdown) |
| Table S33 | Report generated by Gemini 2.5 Pro for *Case IV* (Source markdown) |
| Table S34 | Report generated by DeepSeek R1 for *Case IV* (Source markdown) |
| Table S35 | Report generated by Grok-4 for *Case IV* (Source markdown) |
| Table S36 | Report generated by GPT-4.1 for *Case IV* (Source markdown) |
| Table S37 | Report generated by Qwen-2.5-72b for *Case IV* (Source markdown) |
| Table S38 | Report generated by Gemma-2-27b for *Case IV* (Source markdown) |
| Table S39 | Report generated by Llama-3.1-70b for *Case IV* (Source markdown) |
| Table S40 | Rules for quality checking and filtering implemented (*Case I and II*) |
| Table S41 | Pseudocode for infection progression modelling (*Case I*) |
| Table S42 | Pseudocode for organ damage modelling *(Case II)* |
