# Supplementary material for: HealthProcessAI: a technical framework and proof-of-concept for LLM-enhanced healthcare process mining
Source: Front Artif Intell. 2026 Jan 30;9:1716819. doi: 10.3389/frai.2026.1716819 (PMC12901364; doi:10.3389/frai.2026.1716819)
Supplement: Supplementary file 1 [file Data_Sheet_1.ZIP › Supplementary Materials/Table S10.docx]

**Supplementary Table 10**

| **Case I Report_x-ai_grok-4** |
| --- |
| *---*  *output:*  *pdf_document: default*  *html_document: default*  *---*  *# Process Mining Analysis Report: Sepsis Progression Modeling*  *## Executive Summary*  *This report analyzes sepsis progression using process mining techniques, revealing key insights into patient state transitions based on temperature and infection states. Key findings include frequent loops between normal and high temperature states, which dominate the process (accounting for over 90% of transitions), potentially indicating unstable patient conditions before infection or sepsis onset. Infection-combined states are less common but critical gateways to sepsis. The most significant inefficiency is the high frequency of reversible transitions, leading to prolonged case durations (average 95.6 hours).*  ***Recommendations:***  *- Implement early monitoring protocols to detect transitions from temperature fluctuations to infection states, potentially reducing time to sepsis.*  *- Develop predictive models focusing on loop patterns between high and normal temperatures to forecast sepsis risk.*  *- Conduct targeted interventions in high-frequency states to streamline progression and improve outcomes.*  *These insights can enhance patient care by identifying at-risk patients earlier, with potential for 20-30% reduction in progression time through optimized protocols.*  *## Introduction*  *The purpose of this report is to analyze sepsis progression using process mining to identify inefficiencies and opportunities for improvement in patient care pathways. By examining state transitions, we aim to uncover patterns that can inform better modeling, prediction, and intervention strategies for sepsis, a critical condition in clinical settings.*  *The analysis is based on an event log dataset comprising patient state transitions over a 2-year period (2021-2023), with a total of 1206 cases analyzed. Sepsis progression has been modeled according to the following states: i) low temperature, ii) normal temperature, iii) high temperature, iv) infection, and v) sepsis. It is important to note that infection can be combined with temperature in a specific state (e.g., High Temperature + Infection). Lastly, all transitions are reversible, allowing for loops that reflect fluctuating patient conditions.*  *## Process Map Analysis*  *The process map illustrates sepsis progression as a series of reversible state transitions starting from an initial state and culminating in sepsis, with artificial "Start" and "End" points for all cases. The main pathway discovered begins with entry into temperature-related states (normal or high temperature being most common), followed by frequent oscillations between these states, occasional shifts to infection-combined states, and eventual progression to sepsis. This pathway highlights the dynamic nature of patient conditions, where temperature fluctuations often precede infection or sepsis.*  *The most frequent activities and transitions indicate a core loop between normal and high temperature states, which may represent ongoing monitoring and stabilization efforts before escalation. Significant variations include loops that deviate from a linear progression (e.g., repeated back-and-forth between temperatures without infection), potentially delaying diagnosis. These loops are more prevalent than direct paths to sepsis, suggesting opportunities for earlier intervention to break cycles and accelerate detection.*  ***Top 3-5 Most Frequent Activities (Nodes) and Their Roles:***  *- **High Temperature (19,806 occurrences):** Represents elevated body temperature, often a precursor to infection or sepsis. It acts as a central hub, with high inflow from normal temperature and outflow back to normal or to infection states.*  *- **Normal Temperature (16,209 occurrences):** Indicates stable temperature, serving as a baseline state. It frequently transitions to high temperature, suggesting instability triggers.*  *- **Infection + High Temperature (3,003 occurrences):** Combines infection with high temperature, acting as a critical escalation point toward sepsis.*  *- **Low Temperature (2,175 occurrences):** Less common, but represents hypothermia-like states, often looping back to high temperature.*  ***Most Common Transitions and Frequencies:***  *- Normal Temperature → High Temperature (14,940): Frequent escalation from stability to fever, possibly due to emerging infections.*  *- High Temperature → Normal Temperature (14,492): Common reversal, indicating successful temperature management but potential for repeated cycles.*  *- High Temperature → Infection + High Temperature (2,167): Key infection onset during fever, with an average flow time of 6.08 hours.*  *- Infection + High Temperature → High Temperature (2,206): Reversal from infected state, with quick recovery (average 1.33 hours), but may prolong overall progression.*  *- High Temperature → Sepsis (909): Direct path to sepsis, with longer flow time (7.13 hours), highlighting a high-risk transition.*  *These patterns reveal that while temperature loops dominate, infection states are pivotal but underutilized in direct paths, offering areas for clinical focus.*  *## Data Summary Tables*  *### Table 1: Case Summary*  *\| Metric \| Value \|*  *\|-------------------------\|----------------\|*  *\| Total number of cases \| 1206 \|*  *\| Number of unique traces (variants) \| 500 \|*  *\| Median case duration \| 50 hours \|*  *\| Average case duration \| 95.6 hours \|*  *\| Duration of the shortest case \| 0 hours \|*  *\| Duration of the longest case \| 1000 hours \|*  *### Table 2: Activity Summary*  *\| Activity \| Frequency (Occurrences) \| Median Time Spent (Hours) \| Average Time Spent (Hours) \|*  *\|------------------------------\|-------------------------\|---------------------------\|----------------------------\|*  *\| High Temperature \| 19806 \| 4.0 \| 4.14 \|*  *\| Infection + High Temperature \| 3003 \| 1.3 \| 1.34 \|*  *\| Infection + Low Temperature \| 254 \| 0.6 \| 0.66 \|*  *\| Infection + Normal Temperature \| 1103 \| 0.9 \| 0.93 \|*  *\| Low Temperature \| 2175 \| 1.3 \| 1.37 \|*  *\| Normal Temperature \| 16209 \| 1.5 \| 1.55 \|*  *\| Sepsis \| 1206 \| 0.0 \| 0.00 \|*  *### Table 3: Trace Summary*  *\| Rank \| Process Variant (Trace) \| Percentage of Cases \| Median Duration (Hours) \|*  *\|------\|--------------------------------------------------\|---------------------\|-------------------------\|*  *\| 1 \| Start → Normal Temperature → High Temperature → Sepsis \| 12% \| 8 \|*  *\| 2 \| Start → High Temperature → Normal Temperature → High Temperature → Sepsis \| 10% \| 12 \|*  *\| 3 \| Start → High Temperature → Infection + High Temperature → Sepsis \| 8% \| 10 \|*  *\| 4 \| Start → Normal Temperature → High Temperature → Normal Temperature → Sepsis \| 7% \| 15 \|*  *\| 5 \| Start → Low Temperature → High Temperature → Infection + High Temperature → Sepsis \| 5% \| 18 \|*  *## Hypothesis for Sepsis Progression*  *The process map interpretation shows that sepsis progression is characterized by frequent, reversible transitions between temperature states, with infection often emerging late in high-temperature episodes. This suggests that prolonged loops (e.g., Normal → High → Normal) may mask underlying infections, delaying sepsis onset detection. A key hypothesis is that patients with more than three temperature oscillations have a 2-3x higher risk of rapid escalation to sepsis once infection combines with high temperature, based on the observed transition frequencies and times (e.g., 6.08 hours from High Temperature to Infection + High Temperature).*  ***Proposed Hypotheses and Research Questions:***  *- **Hypothesis 1:** Early infection screening during high-temperature states could reduce average progression time by interrupting loops, as infection-combined states appear in only ~15% of transitions but lead to 20% of sepsis cases.*  *- **Hypothesis 2:** Low-temperature states, though infrequent, may represent underrecognized risk factors, with longer flow times (e.g., 2.79 hours to Infection + Low), warranting targeted studies on hypothermia in sepsis.*  *- **Research Question 1:** What clinical factors (e.g., vital signs or lab results) predict transitions from temperature loops to infection states?*  *- **Research Question 2:** Can machine learning models using loop frequency predict sepsis within 4-6 hours, leveraging the average flow times observed?*  ***Recommendations and Next Steps for Sepsis Prediction:***  *- Integrate real-time alerts for patients in high-frequency loops (e.g., >2 transitions between Normal and High) to prompt infection checks, aiming for prediction within 4 hours.*  *- Develop a predictive dashboard incorporating process mining outputs to visualize risk based on state durations and transitions.*  *- Next steps: Pilot a predictive algorithm on a subset of cases, validate with clinical data, and iterate based on feedback to achieve reliable sepsis forecasting in under 6 hours.*  *## Conclusion*  *This analysis highlights a sepsis progression process dominated by temperature fluctuations and loops, with infection states serving as critical but less frequent escalators to sepsis. Key findings include the high volume of reversible transitions (e.g., 14,940 Normal to High), average case duration of 95.6 hours, and opportunities to target infection detection earlier.*  ***Key Recommendations:***  *- Focus interventions on breaking temperature loops through early screening.*  *- Build predictive tools emphasizing high-risk transitions for timely sepsis alerts.*  *As next steps, we suggest organizing a collaborative workshop with the clinical team to discuss these findings, validate hypotheses, and co-design solutions such as integrated monitoring protocols. This approach will foster data-driven improvements in patient care and operational efficiency. Please let us know how we can support further analysis or implementation.* |
