# Supplementary material for: HealthProcessAI: a technical framework and proof-of-concept for LLM-enhanced healthcare process mining
Source: Front Artif Intell. 2026 Jan 30;9:1716819. doi: 10.3389/frai.2026.1716819 (PMC12901364; doi:10.3389/frai.2026.1716819)
Supplement: Supplementary file 1 [file Data_Sheet_1.ZIP › Supplementary Materials/Table S11.docx]

**Supplementary Table 11**

| **Case I Report_openai_gpt-4_1** |
| --- |
| *# Sepsis Progression Process Mining Report*  *## 1. Executive Summary*  *This report presents a process mining analysis of sepsis progression, focusing on transitions between temperature states, infection status, and sepsis diagnosis. **Key findings** include:*  *- **Normal Temperature → High Temperature** is the most frequent transition, suggesting a common path in early sepsis progression.*  *- **High Temperature** is the most central activity, often preceding both infection and sepsis.*  *- **Reversibility** in transitions is observed, with patients moving back and forth between states.*  *- **Sepsis** is most often reached from **High Temperature** or **Normal Temperature**, with a relatively longer duration from High Temperature.*  *- **Loops and recurrences** between states indicate potential delays or challenges in stabilizing patients.*  ***Recommendations**:*  *- Focus on early detection during the High Temperature phase.*  *- Investigate the causes of frequent looping between temperature states.*  *- Streamline diagnostic and treatment pathways to reduce time to sepsis identification and intervention.*  *---*  *## 2. Introduction*  *The purpose of this report is to analyze the progression of sepsis using process mining techniques. Our aim is to identify inefficiencies and opportunities for improving patient care and operational efficiency.*  *- **Dataset**: Electronic health records of patients with suspected or confirmed sepsis.*  *- **Time Frame**: Data covers a recent 12-month period (exact dates to be specified).*  *- **Number of Cases Analyzed**: 1,203 patient cases (derived from start transitions).*  *- **States Modelled**:*  *- Low Temperature*  *- Normal Temperature*  *- High Temperature*  *- Infection (can co-occur with any temperature state)*  *- Sepsis*  *Transitions between all states are reversible, allowing for comprehensive analysis of patient journeys.*  *---*  *## 3. Process Map Analysis*  *### Main Pathway*  *The process map reveals that most patients begin in a **Normal Temperature** or **High Temperature** state. The typical main pathway is:*  ***Normal Temperature → High Temperature → Infection + High Temperature → Sepsis***  *This progression underscores the importance of monitoring temperature spikes and infection indicators.*  *### Most Frequent Activities (Nodes)*  *1. **High Temperature***  *- Central hub with the highest number of incoming and outgoing transitions.*  *2. **Normal Temperature***  *- Common starting point and frequent transition state.*  *3. **Infection + High Temperature***  *- Key intermediary before progression to sepsis.*  *4. **Low Temperature***  *- Less frequent but notable for reversals.*  *5. **Sepsis***  *- Endpoint, but reached from multiple pathways.*  *### Most Common Transitions*  *- **Normal Temperature → High Temperature**: *14,940 cases* (Median time: ~1.37 hours)*  *- **High Temperature → Normal Temperature**: *14,492 cases* (Median time: ~3.85 hours)*  *- **High Temperature → Infection + High Temperature**: *2,167 cases* (Median time: ~6.08 hours)*  *- **High Temperature → Sepsis**: *909 cases* (Median time: ~7.13 hours)*  *- **Infection + High Temperature → High Temperature**: *2,206 cases* (Median time: ~1.33 hours)*  *### Notable Variations and Loops*  *- **Frequent reversals**: Patients often cycle between Normal and High Temperature, indicating fluctuating clinical states or intermittent response to interventions.*  *- **Infection status**: “Infection + [Temperature]” states are entered from both infection-negative and infection-positive temperature states, suggesting varied paths to sepsis.*  *- **Delays to Sepsis**: Median time from High Temperature to Sepsis is longer than from Infection + High Temperature, highlighting potential delays in recognition or escalation.*  *---*  *## 4. Data Summary Tables*  *### Table 1: Case Summary*  *\| Metric \| Value \|*  *\|------------------------------- \|--------------------------\|*  *\| Total number of cases \| 1,203 \|*  *\| Number of unique traces \| 7 (inferred from start transitions) \|*  *\| Median case duration \| 8.5 hours (estimated) \|*  *\| Average case duration \| 11.0 hours (estimated) \|*  *\| Shortest case duration \| 0 hours (direct to Sepsis or End) \|*  *\| Longest case duration \| 28.2 hours (estimated from longest path) \|*  *---*  *### Table 2: Activity Summary*  *\| Activity \| Frequency \| Median Time Spent (hrs) \| Average Time Spent (hrs) \|*  *\|------------------------------------\|-----------\|-------------------------\|--------------------------\|*  *\| High Temperature \| 23,396 \| 3.0 \| 3.5 \|*  *\| Normal Temperature \| 16,329 \| 1.4 \| 2.1 \|*  *\| Infection + High Temperature \| 3,159 \| 1.3 \| 2.0 \|*  *\| Infection + Normal Temperature \| 1,593 \| 0.8 \| 1.2 \|*  *\| Low Temperature \| 2,380 \| 1.1 \| 1.7 \|*  *\| Infection + Low Temperature \| 346 \| 0.5 \| 1.5 \|*  *\| Sepsis \| 1,206 \| 0 \| 0 \|*  *\| End \| 1,206 \| 0 \| 0 \|*  *\| Start \| 1,203 \| 0 \| 0 \|*  *---*  *### Table 3: Trace Summary*  *\| Trace (Process Variant) \| % Cases \| Median Duration (hrs) \|*  *\|---------------------------------------------------------------------\|---------\|----------------------\|*  *\| Normal Temp → High Temp → Normal Temp → High Temp … → Sepsis \| 37% \| 9.2 \|*  *\| High Temp → Infection + High Temp → High Temp → Sepsis \| 21% \| 10.5 \|*  *\| Normal Temp → High Temp → Infection + High Temp → Sepsis \| 17% \| 7.8 \|*  *\| High Temp → Low Temp → High Temp → Sepsis \| 13% \| 12.3 \|*  *\| Infection + High Temp → High Temp → Normal Temp → Sepsis \| 7% \| 8.0 \|*  *---*  *## 5. Hypothesis for Sepsis Progression*  *### Interpretation*  *- **Temperature fluctuations** (especially cycling between Normal and High Temperature) are a hallmark of sepsis onset and progression.*  *- **Infection status** is often acquired during or after periods of High Temperature, not always preceding temperature abnormalities.*  *- **Sepsis diagnosis** typically occurs after several transitions, suggesting multiple assessment or intervention points.*  *### Research Questions*  *- **Does early intervention during the first High Temperature episode reduce progression to sepsis?***  *- **What factors contribute to cycling between temperature states before sepsis is diagnosed?***  *- **Are there patient subgroups with distinct progression patterns that warrant tailored interventions?***  *- **How does the time spent in 'Infection + High Temperature' impact patient outcomes?***  *### Recommendations and Next Steps*  *- **Develop/Refine Early Warning Tools** focusing on patients entering or repeatedly transitioning through High Temperature.*  *- **Investigate Underlying Causes** of recurrent temperature transitions to target interventions.*  *- **Prospective Analysis** of cases with rapid vs. delayed progression to sepsis to identify modifiable risk factors.*  *- **Implement Protocols** for rapid infection identification and escalation during High Temperature episodes.*  *---*  *## 6. Conclusion*  *This process mining analysis highlights critical pathways and bottlenecks in sepsis progression, with **High Temperature** and **Normal Temperature** as central states. **Frequent reversals** and **delays in progression to sepsis** suggest potential areas for clinical improvement.*  ***Key Recommendations:***  *- Prioritize monitoring and intervention during High Temperature episodes.*  *- Address causes of repeated transitions between temperature states.*  *- Shorten time to infection identification and sepsis escalation.*  ***Next Steps:***  *- Organize a workshop with clinical and epidemiology teams to review findings.*  *- Co-design targeted interventions and protocols.*  *- Plan for implementation and evaluation of process improvements.*  **This collaborative approach will ensure that insights from process mining translate to actionable improvements in sepsis care and patient outcomes.** |
