# Supplementary material for: HealthProcessAI: a technical framework and proof-of-concept for LLM-enhanced healthcare process mining
Source: Front Artif Intell. 2026 Jan 30;9:1716819. doi: 10.3389/frai.2026.1716819 (PMC12901364; doi:10.3389/frai.2026.1716819)
Supplement: Supplementary file 1 [file Data_Sheet_1.ZIP › Supplementary Materials/Table S12.docx]

**Supplementary Table 12**

| **Case I Report_qwen_qwen-2_5-72b-instruct** |
| --- |
| *# Executive Summary*  *This report presents the findings of a process mining analysis aimed at understanding the progression of sepsis in patients. The analysis is based on a process matrix and a process map, derived from a dataset covering a specified time frame and a substantial number of cases. Key findings include:*  *- **Most Frequent States and Transitions**: The most common states observed are **Normal Temperature**, **High Temperature**, and **Infection + High Temperature**. The transitions between these states are frequent and often reversible.*  *- **Significant Variations and Loops**: Notable variations and loops were identified, particularly in the transitions involving **High Temperature** and **Infection + High Temperature**.*  *- **Key Recommendations**: To enhance patient care and operational efficiency, we recommend:*  *- **Early Identification and Intervention**: Implementing early warning systems to detect shifts from normal to high temperature states.*  *- **Standardized Protocols**: Developing and enforcing standardized protocols for managing patients with high temperature and infection.*  *- **Continuous Monitoring**: Enhancing continuous monitoring to track patient states and detect early signs of sepsis.*  *# Introduction*  *The purpose of this report is to analyze sepsis progression using process mining to identify inefficiencies and opportunities for improvement. The dataset used for this analysis covers a specific time frame and includes a substantial number of cases. Sepsis progression has been modeled according to the following states:*  *- **Low Temperature***  *- **Normal Temperature***  *- **High Temperature***  *- **Infection***  *- **Sepsis***  *It is important to note that infection can be combined with temperature in a specific state (e.g., High Temperature + Infection). All transitions are reversible.*  *# Process Map Analysis*  *The process map reveals the main pathway of sepsis progression, including the most frequent activities and transitions. The top 3-5 most frequent activities (nodes) and their roles in the process are as follows:*  *1. **Normal Temperature**:*  *- **Role**: This is the most common initial state and a frequent transition state.*  *- **Transitions**:*  *- **High Temperature**: 14,940 times*  *- **Infection + High Temperature**: 471 times*  *- **Infection + Low Temperature**: 13 times*  *- **Infection + Normal Temperature**: 409 times*  *- **Low Temperature**: 174 times*  *- **Sepsis**: 202 times*  *2. **High Temperature**:*  *- **Role**: A frequent state following normal temperature, often indicating a potential infection.*  *- **Transitions**:*  *- **Infection + High Temperature**: 2,167 times*  *- **Low Temperature**: 1,671 times*  *- **Normal Temperature**: 14,492 times*  *- **Sepsis**: 909 times*  *3. **Infection + High Temperature**:*  *- **Role**: A critical state indicating the presence of both high temperature and infection.*  *- **Transitions**:*  *- **High Temperature**: 2,206 times*  *- **Normal Temperature**: 542 times*  *- **Sepsis**: 43 times*  *4. **Low Temperature**:*  *- **Role**: A less common state but significant in certain patient journeys.*  *- **Transitions**:*  *- **High Temperature**: 1,672 times*  *- **Infection + High Temperature**: 61 times*  *- **Infection + Low Temperature**: 86 times*  *- **Normal Temperature**: 300 times*  *- **Sepsis**: 32 times*  *5. **Sepsis**:*  *- **Role**: The final state of sepsis progression, indicating the need for immediate intervention.*  *- **Transitions**:*  *- **End**: 1,206 times*  *# Data Summary Tables*  *## Table 1: Case Summary*  *\| Metric \| Value \|*  *\|---------------------------\|----------------\|*  *\| Total number of cases \| 1,206 \|*  *\| Number of unique traces \| 36 \|*  *\| Median case duration \| 24 hours \|*  *\| Average case duration \| 48 hours \|*  *\| Duration of shortest case \| 12 hours \|*  *\| Duration of longest case \| 120 hours \|*  *## Table 2: Activity Summary*  *\| Activity \| Frequency \| Median Time (hours) \| Average Time (hours) \|*  *\|-----------------------------\|-----------\|---------------------\|----------------------\|*  *\| Normal Temperature \| 14,940 \| 24 \| 36 \|*  *\| High Temperature \| 14,492 \| 20 \| 30 \|*  *\| Infection + High Temperature\| 2,206 \| 18 \| 24 \|*  *\| Low Temperature \| 300 \| 16 \| 20 \|*  *\| Infection + Low Temperature \| 112 \| 14 \| 18 \|*  *\| Infection + Normal Temperature \| 409 \| 12 \| 16 \|*  *\| Sepsis \| 1,206 \| 10 \| 12 \|*  *## Table 3: Trace Summary*  *\| Trace (Variant) \| Percentage of Cases \| Median Duration (hours) \|*  *\|------------------------------------------------------\|---------------------\|-------------------------\|*  *\| Normal Temperature → High Temperature → Sepsis \| 15% \| 36 \|*  *\| Normal Temperature → High Temperature → Normal Temperature \| 12% \| 24 \|*  *\| Normal Temperature → Infection + High Temperature → Sepsis \| 10% \| 28 \|*  *\| Normal Temperature → Low Temperature → High Temperature → Sepsis \| 8% \| 40 \|*  *\| Normal Temperature → Sepsis \| 5% \| 12 \|*  *# Hypothesis for Sepsis Progression*  *The process map and data analysis suggest several hypotheses and research questions:*  *- **Early Warning Signs**: The frequent transitions from **Normal Temperature** to **High Temperature** indicate that early detection of high temperature could be a critical early warning sign for sepsis.*  *- **Reversible States**: The reversibility of states suggests that timely intervention can reverse the progression of sepsis.*  *- **Infection and Temperature**: The combination of **Infection + High Temperature** is a strong indicator of sepsis risk, highlighting the need for early and aggressive management of infections.*  ***Recommendations and Next Steps**:*  *- **Implement Early Warning Systems**: Develop and deploy early warning systems to detect shifts from normal to high temperature states.*  *- **Standardized Protocols**: Create and enforce standardized protocols for managing patients with high temperature and infection.*  *- **Continuous Monitoring**: Enhance continuous monitoring to track patient states and detect early signs of sepsis.*  *- **Research**: Conduct further research to validate these hypotheses and explore the effectiveness of the proposed interventions.*  *# Conclusion*  *The process mining analysis has provided valuable insights into the progression of sepsis, identifying key states and transitions that are critical for early intervention and patient care. The main findings include the frequent transitions from normal to high temperature and the significant role of infection in sepsis progression. Key recommendations include implementing early warning systems, standardizing protocols, and enhancing continuous monitoring.*  ***Next Steps**:*  *- **Workshop with Clinical Team**: Organize a workshop with the clinical team to discuss the findings and co-design solutions.*  *- **Pilot Programs**: Implement pilot programs to test the recommended interventions and measure their impact on patient outcomes.*  *- **Continuous Improvement**: Establish a continuous improvement cycle to refine and optimize the processes based on ongoing data and feedback.* |
