# Supplementary material for: HealthProcessAI: a technical framework and proof-of-concept for LLM-enhanced healthcare process mining
Source: Front Artif Intell. 2026 Jan 30;9:1716819. doi: 10.3389/frai.2026.1716819 (PMC12901364; doi:10.3389/frai.2026.1716819)
Supplement: Supplementary file 1 [file Data_Sheet_1.ZIP › Supplementary Materials/Table S13.docx]

**Supplementary Table 13**

| **Case I Report_google_gemma-2-27b-it** |
| --- |
| *## Executive Summary*  *This report presents the results of a process mining analysis of sepsis progression using a dataset of [Insert Number] patient cases. The analysis reveals several key findings about the typical progression of sepsis, including the most common pathways and variations from the expected pattern.*  ***Key Findings:***  ** **Predominant Pathway:** The analysis identifies a dominant pathway where patients typically transition from "High Temperature" to "Infection + High Temperature," then potentially to "Sepsis."*  ** **Significant Variations:** While the dominant pathway is common, there are significant variations in the progression, with some patients experiencing "Low Temperature" or "Normal Temperature" states before or during the infection stages.*  ** **Importance of "Normal Temperature" State:** The "Normal Temperature" state appears to play a crucial role in the process, acting as a potential precursor to both "Infection" and "Sepsis."*  ***Recommendations:***  ** Further investigation is needed to understand the factors contributing to the variations observed in sepsis progression.*  ** The prominent role of the "Normal Temperature" state warrants further analysis to determine its predictive value for sepsis development.*  ** A workshop with clinical stakeholders is recommended to discuss these findings and collaboratively develop strategies for improved sepsis prediction and management.*  *## Introduction*  *This report aims to analyze sepsis progression using process mining techniques. The goal is to identify inefficiencies and opportunities for improvement in patient care by understanding how sepsis develops and evolves over time.*  *The analysis is based on a dataset containing [Insert Number] patient cases, covering a time frame of [Insert Time Frame]. The dataset captures patient transitions through five key states representing different stages of sepsis progression:*  ** **Low Temperature:** Characterized by a low body temperature.*  ** **Normal Temperature:** Characterized by a normal body temperature.*  ** **High Temperature:** Characterized by a high body temperature.*  ** **Infection:** Indicates the presence of infection. This state can be combined with temperature states (e.g., "High Temperature + Infection").*  ** **Sepsis:** Represents the culmination of the infectious process, characterized by organ dysfunction.*  *It is important to note that transitions between these states are reversible, meaning a patient can move back and forth between certain states.*  *## Process Map Analysis*  *The process map reveals a primary pathway in sepsis progression, with several notable variations.*  ***Dominant Pathway:***  ** The most frequent activity is **High Temperature**, which occurs in almost all cases, often as the initial state.*  ** From **High Temperature**, the most common transition is to **Infection + High Temperature**.*  ** From **Infection + High Temperature**, the most frequent transition is to **Sepsis**.*  ***Variations and Loops:***  ** **"Normal Temperature" as a Precursor:***  *A significant number of cases transition through the **Normal Temperature** state before **High Temperature** or **Infection**. This suggests that **Normal Temperature** may be an important intermediary state in sepsis development.*  ** **Reverse Transitions:***  *The map shows instances of reverse transitions, such as from **Infection + High Temperature** back to **High Temperature** or **Normal Temperature**. This highlights the dynamic nature of sepsis and the potential for fluctuations in patient condition.*  ***Top 5 Most Frequent Activities:***  ** **High Temperature:** This is the most frequent starting point and a common state throughout the process.*  ** **Infection + High Temperature:** This state represents a significant stage in the progression, often leading to sepsis.*  ** **Normal Temperature:** This state plays a crucial role, potentially acting as a precursor to infection and sepsis.*  ** **Low Temperature:** This state is less frequent but still a notable part of the overall process.*  ** **Sepsis:** This is the final state in the primary pathway, indicating the culmination of the infectious process.*  ***Key Transitions:***  ** **High Temperature to Infection + High Temperature:** This is the most frequent transition, indicating the progression towards a more severe stage of the disease.*  ** **Infection + High Temperature to Sepsis:** This transition marks a critical juncture, signifying the onset of sepsis.*  ** **High Temperature to Normal Temperature:** This transition suggests a potential recovery or stabilization before infection develops.*  *## Data Summary Tables*  *### Table 1: Case Summary*  *\| Metric \| Value \|*  *\|---\|---\|*  *\| Total number of cases \| [Insert Number] \|*  *\| Number of unique traces (variants) \| [Insert Number] \|*  *\| Median case duration \| [Insert Value] \|*  *\| Average case duration \| [Insert Value] \|*  *\| Duration of the shortest case \| [Insert Value] \|*  *\| Duration of the longest case \| [Insert Value] \|*  *### Table 2: Activity Summary*  *\| Activity \| Frequency \| Median Time Spent \| Average Time Spent \|*  *\|---\|---\|---\|---\|*  *\| High Temperature \| [Insert Value] \| [Insert Value] \| [Insert Value] \|*  *\| Infection + High Temperature \| [Insert Value] \| [Insert Value] \| [Insert Value] \|*  *\| Normal Temperature \| [Insert Value] \| [Insert Value] \| [Insert Value] \|*  *\| Low Temperature \| [Insert Value] \| [Insert Value] \| [Insert Value] \|*  *\| Sepsis \| [Insert Value] \| [Insert Value] \| [Insert Value] \|*  *### Table 3: Trace Summary*  *\| Trace \| Percentage of Cases \| Median Duration \|*  *\|---\|---\|---\|*  *\|[Insert Top 5 Most Frequent Traces from Process Mining Analysis]\| [Insert Percentage] \| [Insert Value] \|*  *\|[Insert Top 5 Most Frequent Traces from Process Mining Analysis]\| [Insert Percentage] \| [Insert Value] \|*  *\|[Insert Top 5 Most Frequent Traces from Process Mining Analysis]\| [Insert Percentage] \| [Insert Value] \|*  *\|[Insert Top 5 Most Frequent Traces from Process Mining Analysis]\| [Insert Percentage] \| [Insert Value] \|*  *\|[Insert Top 5 Most Frequent Traces from Process Mining Analysis]\| [Insert Percentage] \| [Insert Value] \|*  *## Hypothesis for Sepsis Progression*  *The process map analysis suggests several hypotheses regarding sepsis progression and opportunities for further research:*  ***1. "Normal Temperature" as a Predictive Marker:***  *Considering the significant role of the "Normal Temperature" state, further research is needed to determine if prolonged periods of "Normal Temperature" after initial infection can be a predictor of sepsis development.*  ***2. Variability in Progression:***  *The observed variations from the dominant pathway raise questions about the factors contributing to these differences. Investigating the potential influence of patient demographics, comorbidities, and treatment strategies on sepsis progression is crucial.*  ***3. Role of Reverse Transitions:***  *Understanding the factors driving reverse transitions, such as from **Infection + High Temperature** back to **High Temperature**, could provide valuable insights into the dynamic nature of sepsis and potential opportunities for intervention.*  ****  *## Conclusion*  *This analysis provides valuable insights into the typical progression of sepsis, highlighting the dominant pathway, significant variations, and the potential role of the "Normal Temperature" state.*  ***Key Recommendations:***  ** **Further research is needed to validate the hypotheses generated from the process map analysis.***  ** **A collaborative workshop with clinical stakeholders is recommended to discuss the findings and co-design solutions for improved sepsis prediction and management.***  *This analysis serves as a starting point for a deeper understanding of sepsis progression, paving the way for more targeted interventions and improved patient outcomes.* |
