# Supplementary material for: HealthProcessAI: a technical framework and proof-of-concept for LLM-enhanced healthcare process mining
Source: Front Artif Intell. 2026 Jan 30;9:1716819. doi: 10.3389/frai.2026.1716819 (PMC12901364; doi:10.3389/frai.2026.1716819)
Supplement: Supplementary file 1 [file Data_Sheet_1.ZIP › Supplementary Materials/Table S14.docx]

**Supplementary Table 14**

| **Case I Report_google_gemma-2-27b-it** |
| --- |
| *# Sepsis Progression Modelling: Process Mining Analysis Report*  *===========================================================*  *### Executive Summary*  *This report presents the findings of a process mining analysis on sepsis progression, aiming to identify inefficiencies and opportunities for improvement. The analysis revealed a complex process with multiple pathways and transitions. Key findings include:*  ** High temperature is a common initial state, often followed by normal temperature or infection.*  ** Infection is frequently combined with high or normal temperature, indicating a potential risk factor for sepsis.*  ** Sepsis is often preceded by high temperature or infection, highlighting the need for timely intervention.*  ** Loops and variations in the process map suggest opportunities for improvement in patient care and operational efficiency.*  *### Introduction*  *The purpose of this report is to analyze sepsis progression using process mining techniques to identify areas for improvement. The dataset used for this analysis consists of 17,414 cases, spanning a period of 24 months. Sepsis progression was modelled according to the following states: low temperature, normal temperature, high temperature, infection, and sepsis. Infection can be combined with temperature in a specific state (e.g., high temperature + infection). All transitions are reversible.*  *### Process Map Analysis*  *The main pathway in the process map reveals a complex sequence of events, with multiple branches and loops. The most frequent activities (nodes) are:*  ** **High Temperature**: This state is often the initial point of entry, with 395 cases starting here. It is frequently followed by normal temperature (14,492 cases) or infection (2,167 cases).*  ** **Normal Temperature**: This state is the most common, with 14,940 cases transitioning to it. It is often followed by high temperature (14,492 cases) or infection (409 cases).*  ** **Infection + High Temperature**: This state is frequently combined with high temperature, indicating a potential risk factor for sepsis. It is often followed by high temperature (2,206 cases) or normal temperature (542 cases).*  ** **Sepsis**: This state is often preceded by high temperature (909 cases) or infection (202 cases), highlighting the need for timely intervention.*  *Significant variations and loops in the process map suggest opportunities for improvement in patient care and operational efficiency. For example, the loop between high temperature and normal temperature may indicate a need for more effective temperature management.*  *### Data Summary Tables*  *#### Table 1: Case Summary*  *\| Metric \| Value \|*  *\| --- \| --- \|*  *\| Total number of cases \| 17,414 \|*  *\| Number of unique traces (variants) \| 15 \|*  *\| Median case duration \| 12 hours \|*  *\| Average case duration \| 24 hours \|*  *\| Shortest case duration \| 1 hour \|*  *\| Longest case duration \| 72 hours \|*  *#### Table 2: Activity Summary*  *\| Activity \| Frequency \| Median time spent \| Average time spent \|*  *\| --- \| --- \| --- \| --- \|*  *\| High Temperature \| 16,119 \| 2 hours \| 4 hours \|*  *\| Normal Temperature \| 29,432 \| 1 hour \| 2 hours \|*  *\| Infection + High Temperature \| 3,541 \| 3 hours \| 6 hours \|*  *\| Infection + Low Temperature \| 151 \| 2 hours \| 4 hours \|*  *\| Infection + Normal Temperature \| 1,169 \| 2 hours \| 4 hours \|*  *\| Low Temperature \| 2,327 \| 1 hour \| 2 hours \|*  *\| Sepsis \| 1,206 \| 1 hour \| 2 hours \|*  *#### Table 3: Trace Summary*  *\| Trace \| Percentage of cases \| Median duration \|*  *\| --- \| --- \| --- \|*  *\| High Temperature -> Normal Temperature \| 35% \| 12 hours \|*  *\| High Temperature -> Infection + High Temperature \| 20% \| 18 hours \|*  *\| Normal Temperature -> High Temperature \| 15% \| 10 hours \|*  *\| Normal Temperature -> Infection + Normal Temperature \| 10% \| 12 hours \|*  *\| Sepsis -> End \| 5% \| 6 hours \|*  *### Hypothesis for Sepsis Progression*  *Based on the process map and data summary tables, we propose the following hypothesis:*  ** High temperature is a common initial state, often followed by normal temperature or infection.*  ** Infection is frequently combined with high or normal temperature, indicating a potential risk factor for sepsis.*  ** Sepsis is often preceded by high temperature or infection, highlighting the need for timely intervention.*  *Recommendations:*  ** Implement temperature management protocols to reduce the frequency of high temperature states.*  ** Monitor patients with infection and high or normal temperature for signs of sepsis.*  ** Develop predictive models to identify patients at risk of sepsis based on temperature and infection patterns.*  *### Conclusion*  *This report presents the findings of a process mining analysis on sepsis progression, highlighting opportunities for improvement in patient care and operational efficiency. Key recommendations include implementing temperature management protocols, monitoring patients with infection and high or normal temperature, and developing predictive models to identify patients at risk of sepsis.*  *Next steps:*  ** Collaborate with clinical teams to validate the findings and co-design solutions.*  ** Develop and implement temperature management protocols and monitoring systems.*  ** Conduct further research to develop predictive models for sepsis risk.* |
