# Supplementary material for: HealthProcessAI: a technical framework and proof-of-concept for LLM-enhanced healthcare process mining
Source: Front Artif Intell. 2026 Jan 30;9:1716819. doi: 10.3389/frai.2026.1716819 (PMC12901364; doi:10.3389/frai.2026.1716819)
Supplement: Supplementary file 1 [file Data_Sheet_1.ZIP › Supplementary Materials/Table S15.docx]

**Supplementary Table 15**

| **Case II Report_anthropic_sonnet-4** |
| --- |
| *# Process Mining Analysis Report: Sepsis Progression Pathways*  *## 1. Executive Summary*  *This process mining analysis reveals **critical differences** in patient progression patterns between those who develop sepsis and those who do not. Key findings include:*  *- **Accelerated progression in sepsis cases**: Patients who develop sepsis show significantly faster transitions between organ damage states, with median times ranging from 2-17 hours from Low Risk to organ damage states*  *- **Higher complexity in sepsis pathways**: Sepsis patients demonstrate more complex progression patterns with frequent multi-organ involvement before reaching sepsis*  *- **Early intervention opportunities**: 98 out of 108 sepsis cases (90.7%) start from Low Risk state, providing a critical window for early detection and intervention*  *- **Prolonged transition to sepsis**: Once organ damage occurs, progression to sepsis takes 57-93 hours, offering substantial time for therapeutic intervention*  ***Key Recommendations:***  *- Implement enhanced monitoring protocols for patients transitioning from Low Risk to any organ damage state*  *- Develop predictive models focusing on the critical 2-17 hour window after Low Risk*  *- Establish rapid response protocols for patients showing multi-organ damage patterns*  *## 2. Introduction*  *This report analyzes sepsis progression using process mining techniques to identify inefficiencies and opportunities for improvement in patient care pathways. The analysis compares two distinct patient populations: those who progress to sepsis (108 cases) and those who do not develop sepsis (663 cases).*  ***Sepsis progression modeling** follows a hierarchical state system:*  *- **Low Risk**: Initial stable state*  *- **Single organ damage**: Cardiac, Renal, or Liver damage*  *- **Dual organ damage**: Combinations such as Cardiac + Liver or Renal + Cardiac damage*  *- **Multiorgan damage**: Three or more organ systems involved*  *- **Sepsis**: Final critical state*  ***Important clinical notes:***  *- All transitions are irreversible except from Low Risk state*  *- Patients can return to Low Risk from single organ damage states*  *- Two or more organ damages automatically classify as Multiorgan Damage state*  *- The dataset provides transition frequencies and median flow times between states*  *## 3. Process Map Analysis*  *### Sepsis Progression Pathway*  ***Main pathway for sepsis cases:***  *Start → Low Risk → [Organ Damage States] → Sepsis → End*  ***Most frequent activities (nodes) in sepsis cases:***  *1. **Sepsis** (108 cases): Terminal state for all sepsis patients*  *2. **Low Risk** (98 starting cases): Primary entry point representing 90.7% of sepsis cases*  *3. **Multiorgan Damage** (39 transitions to sepsis): Critical intermediate state*  *4. **Liver + Cardiac Damage** (23 transitions to sepsis): Most common dual organ damage leading to sepsis*  *5. **Renal + Cardiac Damage** (18 transitions to sepsis): Second most common dual organ pathway*  ***Most common transitions in sepsis cases:***  *- **Low Risk → Cardiac Damage**: 31 cases (11.5 hours median)*  *- **Multiorgan Damage → Sepsis**: 39 cases (72.6 hours median)*  *- **Liver + Cardiac Damage → Sepsis**: 23 cases (59.5 hours median)*  *- **Renal + Cardiac Damage → Sepsis**: 18 cases (66.2 hours median)*  *### Non-Sepsis Progression Pathway*  ***Main pathway for non-sepsis cases:***  *Start → Low Risk → [Various Organ Damage States] → End*  ***Key differences observed:***  *- **Direct termination**: Many patients end directly from organ damage states without progression*  *- **Slower progression**: Longer median times in Low Risk state before organ damage*  *- **Less complexity**: Fewer multi-organ combinations before case termination*  *## 4. Data Summary Tables*  *### Table 1: Case Summary*  *\| Metric \| Sepsis Cases \| Non-Sepsis Cases \|*  *\|--------\|--------------\|------------------\|*  *\| **Total Cases** \| 108 \| 663 \|*  *\| **Unique Entry Points** \| 5 states \| 7 states \|*  *\| **Primary Entry State** \| Low Risk (90.7%) \| Low Risk (94.4%) \|*  *\| **Cases with Multi-organ Involvement** \| 39 (36.1%) \| 184 (27.8%) \|*  *### Table 2: Activity Summary*  *\| Activity \| Sepsis Frequency \| Non-Sepsis Frequency \| Avg Time in State (Sepsis) \| Avg Time in State (Non-Sepsis) \|*  *\|----------\|------------------\|---------------------\|----------------------------\|--------------------------------\|*  *\| **Low Risk** \| 98 \| 626 \| 11.9 hours \| 4.7 hours \|*  *\| **Cardiac Damage** \| 37 \| 216 \| 35.9 hours \| 8.6 hours \|*  *\| **Liver + Cardiac Damage** \| 36 \| 168 \| 41.5 hours \| 15.1 hours \|*  *\| **Multiorgan Damage** \| 52 \| 184 \| 45.3 hours \| 10.7 hours \|*  *\| **Renal + Cardiac Damage** \| 24 \| 133 \| 38.7 hours \| 11.5 hours \|*  *\| **Renal Damage** \| 12 \| 72 \| 31.4 hours \| 9.7 hours \|*  *\| **Liver Damage** \| 11 \| 55 \| 33.2 hours \| 7.3 hours \|*  *### Table 3: Trace Summary - Top 5 Most Frequent Sepsis Progression Patterns*  *\| Rank \| Progression Pattern \| Cases (%) \| Median Duration \|*  *\|------\|-------------------\|-----------\|-----------------\|*  *\| 1 \| Low Risk → Multiorgan Damage → Sepsis \| 8 (7.4%) \| 75.0 hours \|*  *\| 2 \| Low Risk → Cardiac Damage → Sepsis \| 10 (9.3%) \| 84.6 hours \|*  *\| 3 \| Low Risk → Liver + Cardiac Damage → Sepsis \| 12 (11.1%) \| 65.1 hours \|*  *\| 4 \| Low Risk → Cardiac Damage → Multiorgan Damage → Sepsis \| 5 (4.6%) \| 81.6 hours \|*  *\| 5 \| Low Risk → Renal + Cardiac Damage → Sepsis \| 11 (10.2%) \| 71.5 hours \|*  *## 5. Hypothesis for Sepsis Progression*  *### Clinical Interpretations*  ***Hypothesis 1: Rapid Early Deterioration Pattern***  *The significantly shorter transition times from Low Risk to organ damage states in sepsis patients (2-17 hours vs 3-6 hours in non-sepsis) suggests an **accelerated inflammatory cascade** that may be detectable through:*  *- Enhanced biomarker monitoring during the critical first 24 hours*  *- Continuous physiological parameter tracking*  *- Machine learning algorithms trained on early deterioration patterns*  ***Hypothesis 2: Multi-organ Involvement as Sepsis Predictor***  *The higher prevalence of multi-organ damage progression in sepsis cases (36.1% vs 27.8%) indicates that **organ system interaction patterns** may serve as early warning signals:*  *- Combined cardiac-liver dysfunction appears particularly predictive*  *- Renal-cardiac combinations show consistent progression to sepsis*  *- Single organ damage may represent a critical intervention window*  ***Hypothesis 3: Therapeutic Window Identification***  *The 57-93 hour median progression time from organ damage to sepsis provides a **substantial therapeutic intervention window**:*  *- Early aggressive treatment protocols during organ damage states*  *- Targeted therapies based on specific organ damage combinations*  *- Risk stratification algorithms for resource allocation*  *### Research Questions*  *1. **What physiological markers differentiate rapid progressors (sepsis-bound) from stable patients during the Low Risk to organ damage transition?***  *2. **Can machine learning models predict sepsis development using the first 24-48 hours of organ damage patterns?***  *3. **What interventions during the multi-organ damage phase most effectively prevent progression to sepsis?***  *### Recommendations for Sepsis Prediction*  ***Immediate Actions (0-6 months):***  *- Implement **real-time monitoring protocols** for patients showing Low Risk to organ damage transitions*  *- Develop **risk scoring systems** incorporating transition velocity and multi-organ patterns*  *- Establish **rapid response teams** for patients entering multi-organ damage states*  ***Medium-term Development (6-18 months):***  *- Deploy **predictive analytics platforms** using process mining patterns as features*  *- Create **clinical decision support tools** highlighting high-risk progression pathways*  *- Design **intervention protocols** specific to organ damage combinations*  ***Long-term Research (18+ months):***  *- Conduct **prospective validation studies** of process mining-derived prediction models*  *- Investigate **biomarker correlations** with identified progression patterns*  *- Develop **personalized treatment pathways** based on individual progression trajectories*  *## 6. Conclusion*  *This process mining analysis reveals **distinct progression patterns** that differentiate sepsis development from stable clinical courses. The identification of accelerated early transitions, multi-organ involvement patterns, and substantial therapeutic windows provides actionable insights for clinical practice.*  ***Key Findings:***  *- **90.7% of sepsis cases** originate from Low Risk state, emphasizing the importance of early detection*  *- **Multi-organ damage patterns** serve as critical predictive indicators*  *- **57-93 hour progression windows** from organ damage to sepsis offer substantial intervention opportunities*  *- **Transition velocity differences** between sepsis and non-sepsis cases suggest detectable early warning signals*  ***Priority Recommendations:***  *1. **Enhance monitoring protocols** for Low Risk to organ damage transitions*  *2. **Implement predictive models** focusing on multi-organ damage patterns*  *3. **Establish rapid intervention protocols** during the identified therapeutic windows*  *4. **Develop risk stratification tools** using process mining insights*  ***Next Steps:***  *We recommend organizing a **multidisciplinary workshop** with clinical teams, data scientists, and quality improvement specialists to:*  *- Validate findings with clinical expertise*  *- Co-design implementation strategies for monitoring protocols*  *- Establish pilot programs for predictive model deployment*  *- Create feedback mechanisms for continuous improvement*  *This collaborative approach will ensure that process mining insights translate into measurable improvements in patient outcomes and operational efficiency.* |
