# Supplementary material for: HealthProcessAI: a technical framework and proof-of-concept for LLM-enhanced healthcare process mining
Source: Front Artif Intell. 2026 Jan 30;9:1716819. doi: 10.3389/frai.2026.1716819 (PMC12901364; doi:10.3389/frai.2026.1716819)
Supplement: Supplementary file 1 [file Data_Sheet_1.ZIP › Supplementary Materials/Table S16.docx]

**Supplementary Table 16**

| **Case II Report_google_gemini-2_5-pro** |
| --- |
| *# Sepsis Progression Analysis: A Process Mining Report*  *## 1. Executive Summary*  *This report presents a process mining analysis of patient pathways, comparing a cohort of 108 patients who developed sepsis with a cohort of 663 patients who did not. Our analysis reveals starkly different clinical journeys between the two groups, offering critical insights for early identification and intervention.*  ***Key Findings:***  ** **Divergent Pathways:** The non-sepsis patient journey is characterized by rapid transitions (average 5.5 hours) and multiple points of recovery (exit to 'End' state). In stark contrast, the sepsis pathway is a slower, more insidious funnel towards a 'Sepsis' diagnosis, with an average transition time of **45.8 hours**—over eight times longer.*  ** **Critical Transition Window:** The progression from **Multiorgan Damage** to **Sepsis** is the most common final step in the sepsis cohort. This transition takes an average of **72.6 hours**, representing a crucial, albeit late, window for potential intervention.*  ** **Early Warning Signs:** The initial transition from a 'Low Risk' state to the first sign of organ damage is a key differentiator. In patients who ultimately develop sepsis, this first step is significantly slower (e.g., 11.5 hours to develop Cardiac Damage) compared to the non-sepsis group (5.9 hours). This suggests that a slower, less acute onset of organ damage may be a high-risk feature.*  ***Key Recommendations:***  *1. **Refine Alerting Protocols:** Focus predictive models on the characteristics of the *first* organ damage event. A slower, more gradual decline from a 'Low Risk' state should be considered a high-priority flag for sepsis risk.*  *2. **Target the 'Multiorgan Damage' State:** Patients entering a 'Multiorgan Damage' state must be considered at extreme risk. The 72-hour window before a typical sepsis diagnosis is a critical period for aggressive, protocol-driven management.*  *3. **Promote Recovery Pathways:** Emulate the non-sepsis group's trajectory by focusing clinical efforts on resolving initial organ damage to prevent the cascade towards multiorgan failure.*  *---*  *## 2. Introduction*  *The purpose of this report is to analyze and compare the clinical pathways of patients with and without a sepsis diagnosis, using process mining techniques. By visualizing and quantifying patient journeys, we aim to uncover key patterns, identify bottlenecks, and propose data-driven strategies to improve early detection of sepsis and enhance patient outcomes.*  *The analysis is based on retrospective data for two distinct patient groups. The model of disease progression includes the following irreversible states (with the exception of returning from 'Low Risk'):*  ** **Low Risk:** The initial state for most patients.*  ** **Organ Damage States:** Cardiac Damage, Renal Damage, Liver Damage.*  ** **Combined Damage States:** e.g., 'Cardiac + Liver Damage'.*  ** **Multiorgan Damage:** A state representing the combination of two or more distinct organ damages.*  ** **Sepsis:** The final adverse outcome state for one cohort.*  *This analysis focuses on the transitions *between* these states to understand the flow and timing of disease progression.*  *---*  *## 3. Process Map Analysis*  *The process mining analysis reveals two fundamentally different "maps" for patient progression.*  *#### Sepsis Patient Pathway (108 Cases)*  *The journey for patients who develop sepsis is best described as a **funnel**. Almost all patients (91%) start in a 'Low Risk' state and progress sequentially through one or more organ damage states before being diagnosed with Sepsis.*  ** **Main Entry Point:** The most frequent initial event is the transition from **`Low Risk` -> `Cardiac Damage`** (31 instances). This is the primary gateway out of the low-risk category.*  ** **Central Hub of Failure:** The **`Multiorgan Damage`** state is a critical precursor to sepsis. It is the most common direct antecedent, with the transition **`Multiorgan Damage` -> `Sepsis`** occurring 39 times.*  ** **Slow Progression:** A defining characteristic of this group is the long duration of transitions leading to the 'Sepsis' state. For example, the average time from entering 'Multiorgan Damage' to a 'Sepsis' diagnosis is **72.6 hours**. This indicates a prolonged period of critical illness preceding the formal diagnosis.*  *#### Non-Sepsis Patient Pathway (663 Cases)*  *The journey for patients who do not develop sepsis is a **branching tree with multiple exits**. Patients experience organ damage but frequently stabilize and recover, represented by transitions to an 'End' state from various points.*  ** **High-Volume Entry:** Similar to the sepsis group, the most common starting pathway is **`Low Risk` -> `Cardiac Damage`** (204 instances).*  ** **Frequent Recovery:** Unlike the sepsis group, recovery is common. The most frequent transitions in the entire process are those leading to resolution, such as **`Liver + Cardiac Damage` -> `End`** (148 cases) and **`Renal + Cardiac Damage` -> `End`** (140 cases).*  ** **Rapid Transitions:** The time between states is significantly shorter. The average transition from **`Low Risk` -> `Cardiac Damage`** is only **5.9 hours**, compared to 11.5 hours in the sepsis group. This suggests a more acute, but ultimately resolvable, event.*  *---*  *## 4. Data Summary Tables*  ****Note on Metrics:*** *The provided data is an aggregated summary of transitions, not a complete event log. Therefore, metrics like "Unique Traces" and "Case Duration" cannot be precisely calculated. The tables below use available data to provide the best possible summary. "Average Duration" in Table 1 refers to the weighted average time of a single transition, not the total patient journey.**  *#### Table 1: Case Summary*  *\| Metric \| Sepsis Group \| Non-Sepsis Group \| Key Difference \|*  *\| :--- \| :--- \| :--- \| :--- \|*  *\| **Total Number of Cases** \| 108 \| 663 \| - \|*  *\| **Unique Traces (Variants)**\| Not Available \| Not Available \| - \|*  *\| **Weighted Avg. Transition Duration** \| **45.8 hours** \| **5.5 hours** \| Sepsis pathway transitions are **8x slower** on average. \|*  *\| **Longest Avg. Transition** \| `Liver Damage -> Sepsis` (93h) \| `Liver + Cardiac -> Multiorgan` (25h) \| Longest transitions in the sepsis group lead to Sepsis. \|*  *\| **Shortest Avg. Transition**\| `Low Risk -> Liver + Renal` (2.3h) \| `Low Risk -> Multiorgan` (2.9h) \| Initial transitions can be rapid in both groups. \|*  *#### Table 2: Activity (State) Summary*  *This table shows the frequency of each clinical state and the average time it took for patients to transition *out* of that state.*  *\| Activity (State) \| Sepsis Group Freq. \| Sepsis Group Avg. Time to Exit \| Non-Sepsis Group Freq. \| Non-Sepsis Group Avg. Time to Exit \|*  *\| :--- \| :--- \| :--- \| :--- \| :--- \|*  *\| **Low Risk** \| 98 \| 15.1 hours \| 626 \| 5.0 hours \|*  *\| **Cardiac Damage** \| 37 \| 39.5 hours \| 216 \| 10.9 hours \|*  *\| **Renal Damage** \| 12 \| 26.6 hours \| 72 \| 13.1 hours \|*  *\| **Liver Damage** \| 12 \| 27.3 hours \| 55 \| 8.0 hours \|*  *\| **Renal + Cardiac Damage** \| 24 \| 48.0 hours \| 123 \| 18.2 hours \|*  *\| **Liver + Cardiac Damage** \| 29 \| 51.1 hours \| 144 \| 14.1 hours \|*  *\| **Multiorgan Damage** \| 68 \| 72.6 hours \| 154 \| 14.6 hours \|*  *#### Table 3: Top 5 Most Frequent Transitions*  *\| Rank \| Sepsis Group Transition \| Count \| Avg. Duration (hrs) \|*  *\| :--- \| :--- \| :--- \| :--- \|*  *\| 1 \| `Multiorgan Damage -> Sepsis` \| 39 \| 72.6 \|*  *\| 2 \| `Low Risk -> Cardiac Damage` \| 31 \| 11.5 \|*  *\| 3 \| `Liver + Cardiac Damage -> Sepsis`\| 23 \| 59.5 \|*  *\| 4 \| `Renal + Cardiac Damage -> Sepsis`\| 18 \| 66.2 \|*  *\| 5 \| `Renal + Cardiac -> Multiorgan` \| 13 \| 22.7 \|*  *\| Rank \| Non-Sepsis Group Transition \| Count \| Avg. Duration (hrs) \|*  *\| :--- \| :--- \| :--- \| :--- \|*  *\| 1 \| `Low Risk -> Cardiac Damage` \| 204 \| 5.9 \|*  *\| 2 \| `Liver + Cardiac Damage -> End` \| 148 \| 0.0 \|*  *\| 3 \| `Renal + Cardiac Damage -> End` \| 140 \| 0.0 \|*  *\| 4 \| `Cardiac Damage -> End` \| 105 \| 0.0 \|*  *\| 5 \| `Low Risk -> Liver + Cardiac` \| 90 \| 3.6 \|*  *---*  *## 5. Hypothesis for Sepsis Progression*  *This analysis provides a new lens through which to view sepsis progression, leading to several testable hypotheses and research questions.*  ***Hypothesis 1: The "Slow Burn" Onset***  *The initial transition from 'Low Risk' to the first organ damage is significantly slower in patients who will develop sepsis (e.g., 11.5h vs. 5.9h for cardiac damage). This contradicts the idea that a faster decline is always worse. It may be that a slower, more insidious onset allows for compensatory mechanisms to be exhausted, leading to an irreversible cascade.*  ** **Research Question:** Do patients with a slower, sub-acute initial rise in organ damage markers (e.g., troponin, creatinine) have a higher likelihood of developing sepsis compared to those with a sharp, acute rise who then recover?*  ** **Recommendation:** Predictive models should incorporate not just the presence of organ damage, but also the *rate of onset*. A slow, steady decline over 12-24 hours may be a more potent predictor of sepsis than a rapid spike.*  ***Hypothesis 2: 'Multiorgan Damage' is the Point of No Return***  *In the sepsis cohort, entry into the 'Multiorgan Damage' state is a near-terminal event, initiating a 72-hour countdown to a sepsis diagnosis. In the non-sepsis group, this state is still survivable and transitions to 'End' are possible (though not shown in top 5, they exist in the data).*  ** **Research Question:** What specific clinical or biological factors differentiate patients who recover from 'Multiorgan Damage' versus those who progress to sepsis?*  ** **Recommendation:** Implement an immediate, high-intensity care bundle for any patient entering the 'Multiorgan Damage' state. This is the last, best window to alter the patient's trajectory.*  ***Hypothesis 3: Cardiac Damage as a Primary Gateway***  *In both cohorts, 'Cardiac Damage' is the most common first step away from a 'Low Risk' state. However, its consequences are dramatically different. In the non-sepsis group, it often leads to recovery. In the sepsis group, it is a definitive step towards further deterioration.*  ** **Research Question:** Are there different etiologies or phenotypes of initial 'Cardiac Damage' that determine these divergent paths?*  ** **Recommendation:** Further stratify patients with initial cardiac damage. Those with concurrent risk factors (e.g., rising inflammatory markers, borderline kidney function) should be monitored with the assumption they are on the sepsis pathway until proven otherwise.*  *---*  *## 6. Conclusion*  *Process mining has provided a powerful, visual, and quantitative confirmation of what clinicians often observe: the pathway to sepsis is distinct, insidious, and slow-moving compared to more acute, recoverable conditions. The key takeaway is that the timing and character of disease progression, not just the presence of organ damage, are critical for risk stratification.*  ***Key Findings Summarized:***  ** The sepsis pathway is a slow funnel (avg. transition 45.8h); the non-sepsis pathway is a fast, branching tree of recovery (avg. transition 5.5h).*  ** A slow onset of initial organ damage is a high-risk feature.*  ** The transition from `Multiorgan Damage -> Sepsis` is a 72-hour window for final intervention.*  ***Next Steps:***  *We recommend a collaborative workshop with the clinical and epidemiological teams to:*  *1. **Validate These Findings:** Discuss these process maps in the context of clinical experience.*  *2. **Refine Hypotheses:** Brainstorm clinical or biological reasons for the observed differences in timing and pathways.*  *3. **Co-design Interventions:** Develop concrete changes to protocols and predictive models based on these insights, particularly focusing on the rate of onset and the management of patients in the 'Multiorgan Damage' state.* |
