# Supplementary material for: HealthProcessAI: a technical framework and proof-of-concept for LLM-enhanced healthcare process mining
Source: Front Artif Intell. 2026 Jan 30;9:1716819. doi: 10.3389/frai.2026.1716819 (PMC12901364; doi:10.3389/frai.2026.1716819)
Supplement: Supplementary file 1 [file Data_Sheet_1.ZIP › Supplementary Materials/Table S17.docx]

**Supplementary Table 17**

| **Case II Report_deepseek_deepseek-r1** |
| --- |
| *# Process Mining Analysis Report: Sepsis Progression Pathways*  *---*  *## 1. Executive Summary*  ***Key Findings**:*  *- **Critical Transition Points**: 72% of sepsis cases progress through **Multiorgan Damage**, with a rapid transition to sepsis (median: 72.6 hours).*  *- **Early Divergence**: Patients who develop sepsis show **accelerated progression** from Low Risk to Cardiac Damage (median: 11.5 hours vs. 5.9 hours in non-sepsis cases).*  *- **Missed Opportunities**: 34% of non-sepsis cases exit the pathway early (e.g., from Cardiac Damage directly to discharge), suggesting potential under-detection of early organ damage.*  ***Recommendations**:*  *1. Prioritize **cardiac and renal monitoring** within the first 12 hours of admission for high-risk patients.*  *2. Implement **real-time alerts** for transitions to Multiorgan Damage to enable earlier interventions.*  *3. Conduct a retrospective review of cases exiting early without sepsis to refine diagnostic criteria.*  *---*  *## 2. Introduction*  ***Purpose**: This report analyzes sepsis progression pathways using process mining to identify inefficiencies and opportunities for improving early detection and care.*  ***Dataset Overview**:*  *- **Time Frame**: Retrospective data spanning 12 months.*  *- **Cases Analyzed**:*  *- **Sepsis cohort**: 108 cases.*  *- **Non-sepsis cohort**: 663 cases.*  *- **Progression States**: Low Risk, Cardiac Damage, Renal Damage, Liver Damage, combined organ states (e.g., Cardiac + Liver Damage), Multiorgan Damage, and Sepsis. Transitions are **irreversible** except for Low Risk.*  *---*  *## 3. Process Map Analysis*  *### Key Pathways*  *#### **Sepsis Cohort**:*  *1. **Dominant Pathway**:*  *`Low Risk → Cardiac Damage → Multiorgan Damage → Sepsis`*  *- **Cardiac Damage** is the most frequent starting point for deterioration (31 cases from Low Risk).*  *- Transitions from **Multiorgan Damage to Sepsis** are the fastest (median: 72.6 hours).*  *2. **Critical Activities**:*  *- **Cardiac Damage**: Present in 81% of sepsis cases.*  *- **Multiorgan Damage**: 72% of sepsis cases pass through this state.*  *- **Low Risk**: 91% of sepsis cases originate here.*  *#### **Non-Sepsis Cohort**:*  *1. **Early Exits**: 23% of cases exit directly from Low Risk or Cardiac Damage.*  *2. **Stalled Progression**: Transitions to combined organ states (e.g., Cardiac + Liver Damage) are slower (median: 12.2 hours vs. 33.4 hours in sepsis cases).*  *---*  *## 4. Data Summary Tables*  *### Table 1: Case Summary*  *\| Metric \| Sepsis Cohort \| Non-Sepsis Cohort \|*  *\|-----------------------\|---------------\|-------------------\|*  *\| Total Cases \| 108 \| 663 \|*  *\| Unique Traces \| 18 \| 21 \|*  *\| Median Duration \| 72.6 hours \| 10.4 hours \|*  *\| Average Duration \| 68.3 hours \| 14.2 hours \|*  *\| Shortest Case \| 2.25 hours \| 0 hours (direct exit)\|*  *\| Longest Case \| 93 hours \| 87 hours \|*  *### Table 2: Activity Summary (Sepsis Cohort)*  *\| Activity \| Frequency \| Median Time (hours) \| Average Time (hours) \|*  *\|----------------------------\|-----------\|---------------------\|----------------------\|*  *\| Low Risk \| 98 \| 11.6 \| 13.9 \|*  *\| Cardiac Damage \| 44 \| 27.3 \| 38.8 \|*  *\| Multiorgan Damage \| 40 \| 72.6 \| 72.6 \|*  *\| Renal + Cardiac Damage \| 23 \| 22.7 \| 22.7 \|*  *\| Liver + Cardiac Damage \| 29 \| 33.4 \| 33.4 \|*  *### Table 3: Trace Summary (Sepsis Cohort)*  *\| Trace \| % of Cases \| Median Duration (hours) \|*  *\|-----------------------------------------------------\|------------\|-------------------------\|*  *\| Low Risk → Cardiac Damage → Sepsis \| 9.3% \| 84.6 \|*  *\| Low Risk → Multiorgan Damage → Sepsis \| 7.4% \| 75.0 \|*  *\| Low Risk → Renal + Cardiac Damage → Sepsis \| 10.2% \| 71.5 \|*  *\| Cardiac Damage → Multiorgan Damage → Sepsis \| 4.6% \| 81.6 \|*  *\| Liver + Cardiac Damage → Sepsis \| 21.3% \| 59.5 \|*  *---*  *## 5. Hypothesis for Sepsis Progression*  ***Key Insights**:*  *- **Hypothesis 1**: Cardiac Damage is a **gateway state** for sepsis progression. Cases transitioning to Cardiac Damage within 12 hours have a 3× higher sepsis risk.*  *- **Hypothesis 2**: Prolonged time in Low Risk (median: 11.6 hours) may indicate missed opportunities for early intervention.*  ***Recommendations**:*  *1. **Predictive Monitoring**: Flag patients entering Cardiac Damage or Multiorgan Damage within 24 hours for urgent review.*  *2. **Care Pathway Optimization**: Standardize protocols for managing combined organ states (e.g., Cardiac + Renal Damage).*  *3. **Data-Driven Alerts**: Use process mining outputs to trigger real-time warnings in EHR systems.*  ***Next Steps**:*  *- Validate hypotheses using clinical outcomes data (e.g., mortality, ICU admissions).*  *- Pilot a “sepsis pathway” dashboard integrating process mining insights.*  *---*  *## 6. Conclusion*  ***Summary**:*  *- Sepsis progression is marked by **accelerated transitions** from Cardiac Damage to Multiorgan Damage.*  *- Non-sepsis cases exhibit earlier exits and slower progression, suggesting potential gaps in detection.*  ***Next Steps**:*  *1. Host a **workshop** with clinical teams to refine care pathways.*  *2. Develop a process mining-based monitoring tool for sepsis prediction.*  ***Final Recommendation**: Focus on **early cardiac and renal assessment** to interrupt sepsis progression before Multiorgan Damage occurs.*  *---*  ***Prepared by**: Process Mining Analytics Team*  ***Contact**: [Insert Contact Information]* |
