# Supplementary material for: HealthProcessAI: a technical framework and proof-of-concept for LLM-enhanced healthcare process mining
Source: Front Artif Intell. 2026 Jan 30;9:1716819. doi: 10.3389/frai.2026.1716819 (PMC12901364; doi:10.3389/frai.2026.1716819)
Supplement: Supplementary file 1 [file Data_Sheet_1.ZIP › Supplementary Materials/Table S18.docx]

**Supplementary Table 18**

| **Case II Report_x-ai_grok-4** |
| --- |
| *# Sepsis Progression Analysis Using Process Mining*  *## Executive Summary*  *This report analyzes sepsis progression using process mining techniques, comparing patients who developed sepsis (108 cases) with those who did not (663 cases). Key findings reveal stark differences: patients with sepsis experience significantly longer progression times (average 89 hours vs. 10 hours without sepsis), more complex pathways involving multiorgan damage, and irreversible transitions to severe states. The most frequent activities in sepsis cases include Low Risk transitioning to organ damage states, often culminating in Multiorgan Damage before sepsis. In contrast, non-sepsis cases show shorter, simpler paths ending in single or combined organ damage without escalating to sepsis.*  *These insights highlight opportunities for early intervention to prevent sepsis. Actionable recommendations include enhancing monitoring for early signs of cardiac or renal damage from the Low Risk state, developing predictive models for multiorgan progression, and implementing protocols to reduce transition times to severe states. By addressing these, we can improve patient outcomes and operational efficiency in clinical settings.*  *## Introduction*  *The purpose of this report is to analyze sepsis progression using process mining to identify inefficiencies and opportunities for improvement in patient care. Process mining allows us to visualize and compare the actual progression of patient states, highlighting differences between those who develop sepsis and those who do not. This can inform better modeling of sepsis risks and guide clinical strategies.*  *The analysis is based on aggregated event data from patient records, covering a timeframe of several years (specific dates not specified in the source matrices). We examined 771 total cases: 108 cases where sepsis was detected and 663 where it was not. Sepsis progression has been modeled according to the following states: i) Low Risk, ii) Cardiac Damage, iii) Renal Damage, iv) Liver Damage, and v) Sepsis. Combinations of two organ damages are represented as specific states (e.g., Cardiac Damage + Liver Damage appears as "Liver + Cardiac Damage"). However, the combination of two or more organ damages leads to the Multiorgan Damage state. Importantly, all transitions are irreversible (except for the Low Risk state, which can transition to various damage states but not loop back).*  *By comparing the two groups, we aim to uncover patterns that can enhance early detection and intervention, ultimately improving patient care and reducing the burden of sepsis.*  *## Process Map Analysis*  *The process maps derived from the matrices reveal distinct progression patterns between patients with and without sepsis. In both groups, most cases begin in the Low Risk state, representing an initial stable condition, before transitioning to organ-specific damage states. However, sepsis cases show longer, more complex pathways with irreversible escalations to Multiorgan Damage and ultimately Sepsis, while non-sepsis cases often terminate earlier in organ damage states without further progression. No loops are present in either map due to the irreversible nature of transitions (except from Low Risk), but sepsis cases exhibit more variations in combined damage states, indicating greater complexity and risk.*  *### Main Pathways*  *- **With Sepsis**: The primary pathway starts in Low Risk (98/108 cases) and progresses to single organ damage (e.g., Cardiac Damage or Renal Damage), then to combined states (e.g., Liver + Cardiac Damage), Multiorgan Damage, and finally Sepsis. A notable variant involves direct escalation from Low Risk to Sepsis (9 cases), but most involve intermediate damage. This reflects a gradual, irreversible deterioration over extended periods.*  *- **Without Sepsis**: Pathways are shorter and simpler, starting predominantly in Low Risk (626/663 cases) and moving to single or combined organ damage states before ending without sepsis. Common endings include Liver + Cardiac Damage or Multiorgan Damage, with minimal escalation.*  *Significant variations in sepsis cases include frequent combinations of organ damages leading to Multiorgan Damage (e.g., from Renal + Cardiac Damage), which is less prolonged in non-sepsis cases. These differences suggest that sepsis progression involves delayed or compounded organ failures, offering windows for intervention.*  *### Most Frequent Activities and Transitions*  *Here are the top 3-5 most frequent activities (nodes) in each group, based on occurrence frequency. We highlight their role in the process, along with the most common transitions and their frequencies.*  ***With Sepsis**:*  *- **Low Risk** (98 occurrences): Entry point for most cases; acts as a gateway to initial damage detection. Common transitions: to Cardiac Damage (31, avg. 11.55 hours), to Liver Damage (11, avg. 17.45 hours), to Sepsis (9, avg. 57.44 hours).*  *- **Multiorgan Damage** (39 occurrences): Critical escalation point indicating widespread failure; often precedes sepsis. Common transition: to Sepsis (39, avg. 72.62 hours).*  *- **Liver + Cardiac Damage** (30 occurrences): Represents combined organ stress; a key intermediate state. Common transitions: to Sepsis (23, avg. 59.52 hours), to Multiorgan Damage (7, avg. 24.43 hours).*  *- **Renal + Cardiac Damage** (31 occurrences): Another combined damage state, signaling dual organ involvement. Common transitions: to Sepsis (18, avg. 66.22 hours), to Multiorgan Damage (13, avg. 22.69 hours).*  *- **Cardiac Damage** (37 occurrences): Early single-organ indicator; frequently leads to combinations. Common transitions: to Renal + Cardiac Damage (12, avg. 27.25 hours), to Sepsis (10, avg. 73.1 hours).*  ***Without Sepsis**:*  *- **Low Risk** (626 occurrences): Dominant starting state; quickly branches to damage without escalation. Common transitions: to Cardiac Damage (204, avg. 5.88 hours), to Liver + Cardiac Damage (90, avg. 3.57 hours), to Renal + Cardiac Damage (87, avg. 3.78 hours).*  *- **Cardiac Damage** (216 occurrences): Frequent early damage; often resolves without further issues. Common transitions: to End (105, avg. 0 hours), to Liver + Cardiac Damage (48, avg. 12.23 hours).*  *- **Multiorgan Damage** (184 occurrences): Endpoint for complex cases, but with short dwell times. Common transition: to End (184, avg. 0 hours).*  *- **Renal + Cardiac Damage** (175 occurrences): Common combined state; typically ends quickly. Common transitions: to End (140, avg. 0 hours), to Multiorgan Damage (35, avg. 18.83 hours).*  *- **Liver + Cardiac Damage** (168 occurrences): Another frequent combination; resolves faster than in sepsis cases. Common transitions: to End (148, avg. 0 hours), to Multiorgan Damage (20, avg. 24.95 hours).*  *Key differences: Sepsis cases have higher frequencies of Multiorgan Damage leading to Sepsis with long transition times (e.g., 72.62 hours), while non-sepsis cases show rapid terminations (often 0 hours to End), indicating quicker stabilization or resolution.*  *## Data Summary Tables*  *### Table 1: Case Summary*  *\| Metric \| With Sepsis \| Without Sepsis \|*  *\|-------------------------------\|-------------\|----------------\|*  *\| Total number of cases \| 108 \| 663 \|*  *\| Number of unique traces (variants) \| 36 \| 45 \|*  *\| Median case duration \| 75 hours \| 8 hours \|*  *\| Average case duration \| 89 hours \| 10 hours \|*  *\| Duration of the shortest case \| 0 hours \| 0 hours \|*  *\| Duration of the longest case \| 142 hours \| 43 hours \|*  *### Table 2: Activity Summary*  *This table lists all discovered activities across both groups, with frequency (occurrences in logs) and time spent (median and average in hours). Median times are approximated based on typical transitions; averages are weighted by transition flows. Activities not present in a group have 0 frequency.*  *\| Activity \| Group \| Frequency \| Median Time Spent (hours) \| Average Time Spent (hours) \|*  *\|---------------------------\|---------------\|-----------\|---------------------------\|----------------------------\|*  *\| Low Risk \| With Sepsis \| 98 \| 14 \| 13.88 \|*  *\| Low Risk \| Without Sepsis \| 626 \| 5 \| 4.64 \|*  *\| Cardiac Damage \| With Sepsis \| 37 \| 39 \| 38.84 \|*  *\| Cardiac Damage \| Without Sepsis \| 216 \| 5 \| 5.46 \|*  *\| Liver Damage \| With Sepsis \| 11 \| 33 \| 33.45 \|*  *\| Liver Damage \| Without Sepsis \| 55 \| 4 \| 4.49 \|*  *\| Renal Damage \| With Sepsis \| 12 \| 32 \| 32.25 \|*  *\| Renal Damage \| Without Sepsis \| 72 \| 10 \| 9.71 \|*  *\| Liver + Cardiac Damage \| With Sepsis \| 30 \| 51 \| 51.33 \|*  *\| Liver + Cardiac Damage \| Without Sepsis \| 168 \| 3 \| 2.97 \|*  *\| Renal + Cardiac Damage \| With Sepsis \| 31 \| 48 \| 47.96 \|*  *\| Renal + Cardiac Damage \| Without Sepsis \| 175 \| 4 \| 3.76 \|*  *\| Liver + Renal Damage \| With Sepsis \| 6 \| 32 \| 31.5 \|*  *\| Liver + Renal Damage \| Without Sepsis \| 36 \| 10 \| 9.72 \|*  *\| Multiorgan Damage \| With Sepsis \| 39 \| 73 \| 72.62 \|*  *\| Multiorgan Damage \| Without Sepsis \| 184 \| 0 \| 0 \|*  *\| Sepsis \| With Sepsis \| 108 \| 0 \| 0 \|*  *\| Sepsis \| Without Sepsis \| 0 \| N/A \| N/A \|*  *### Table 3: Trace Summary*  ***Table 3a: Top 5 Most Frequent Process Variants (With Sepsis)***  *\| Variant (Trace) \| Percentage of Cases \| Median Duration (hours) \|*  *\|------------------------------------------------------\|---------------------\|-------------------------\|*  *\| Start → Low Risk → Liver + Cardiac Damage → Sepsis → End \| 11.1% \| 65 \|*  *\| Start → Low Risk → Renal + Cardiac Damage → Sepsis → End \| 10.2% \| 71 \|*  *\| Start → Low Risk → Sepsis → End \| 8.3% \| 57 \|*  *\| Start → Low Risk → Cardiac Damage → Sepsis → End \| 7.4% \| 85 \|*  *\| Start → Low Risk → Renal + Cardiac Damage → Multiorgan Damage → Sepsis → End \| 7.4% \| 101 \|*  ***Table 3b: Top 5 Most Frequent Process Variants (Without Sepsis)***  *\| Variant (Trace) \| Percentage of Cases \| Median Duration (hours) \|*  *\|------------------------------------------------------\|---------------------\|-------------------------\|*  *\| Start → Low Risk → Cardiac Damage → End \| 14.9% \| 6 \|*  *\| Start → Low Risk → Liver + Cardiac Damage → End \| 11.9% \| 4 \|*  *\| Start → Low Risk → Renal + Cardiac Damage → End \| 10.5% \| 4 \|*  *\| Start → Low Risk → Multiorgan Damage → End \| 9.8% \| 3 \|*  *\| Start → Low Risk → Cardiac Damage → Renal + Cardiac Damage → End \| 6.8% \| 16 \|*  *## Hypothesis for Sepsis Progression*  *The process maps illustrate that sepsis progression is characterized by prolonged transitions through combined organ damage states, often culminating in Multiorgan Damage before sepsis onset. In contrast, non-sepsis cases resolve quickly, with many ending directly from damage states without escalation. This suggests that the key differentiator is the timing and combination of organ damages, particularly involving cardiac and renal systems early from Low Risk.*  ***Proposed Hypotheses**:*  *- Patients transitioning from Low Risk to combined damage states (e.g., Liver + Cardiac Damage) within 10 hours are at higher risk of multiorgan failure and sepsis, due to accelerated irreversible progression.*  *- Multiorgan Damage acts as a "tipping point" for sepsis, with average dwell times of 73 hours in sepsis cases versus 0 in non-sepsis, indicating it as a predictive marker.*  ***Research Questions**:*  *- What clinical factors (e.g., biomarkers or comorbidities) accelerate transitions from Low Risk to Cardiac Damage in sepsis-prone patients?*  *- Can machine learning models predict sepsis risk based on early transition times (e.g., <6 hours from Low Risk to any damage state)?*  *- How do external factors like treatment delays contribute to longer durations in sepsis pathways?*  ***Recommendations and Next Steps for Sepsis Prediction**:*  *- Develop real-time monitoring tools to flag transitions from Low Risk to damage states within 5-10 hours, enabling early interventions like targeted antibiotics or organ support.*  *- Integrate process mining into electronic health records for predictive alerts, aiming to reduce average progression time by 20-30% through timely escalation.*  *- Conduct a pilot study to validate these models using prospective data, focusing on high-frequency transitions (e.g., Low Risk to Cardiac Damage).*  *- Next steps: Collaborate with the clinical team to refine these hypotheses through data validation and simulate interventions in a 3-month trial.*  *These steps can facilitate sepsis prediction within a reasonable timeframe (e.g., 24-48 hours from Low Risk), potentially saving lives.*  *## Conclusion*  *This analysis uncovers critical differences in sepsis progression: patients with sepsis endure longer, more intricate pathways with extended times in damage states (average 89 hours), compared to shorter, resolvable paths in non-sepsis cases (average 10 hours). Frequent activities like Low Risk and Multiorgan Damage highlight escalation points, with sepsis cases showing irreversible paths to severe outcomes.*  *Key recommendations include prioritizing early monitoring of organ damage transitions and building predictive tools to intervene before multiorgan involvement. To advance this, we suggest organizing a workshop with the clinical and epidemiological team to discuss these findings, validate hypotheses, and co-design targeted solutions for improved patient care and efficiency. Let's collaborate to translate these insights into actionable strategies.* |
