# Supplementary material for: HealthProcessAI: a technical framework and proof-of-concept for LLM-enhanced healthcare process mining
Source: Front Artif Intell. 2026 Jan 30;9:1716819. doi: 10.3389/frai.2026.1716819 (PMC12901364; doi:10.3389/frai.2026.1716819)
Supplement: Supplementary file 1 [file Data_Sheet_1.ZIP › Supplementary Materials/Table S19.docx]

**Supplementary Table 18**

| **Case II Report_openai_gpt-4_1** |
| --- |
| *# Sepsis Progression Process Mining Analysis*  *## 1. Executive Summary*  *This report presents a comparative process mining analysis of patient progression with and without sepsis, focusing on critical organ damage states and their transitions. Key findings include:*  *- **Faster progression to multiorgan damage and sepsis** among patients who develop sepsis, often following combinations of organ damage.*  *- **Higher frequency and earlier onset of multiorgan involvement in sepsis cases**, with transitions that are both more numerous and rapid compared to non-sepsis cases.*  *- **Distinct process variants and durations** between the two groups, highlighting opportunities for earlier intervention and risk stratification.*  *- **Recommendation:** Prioritize early identification of organ damage combinations (especially cardiac and renal involvement), as these are strong precursors to sepsis. Enhance monitoring and intervention protocols in the early "Low Risk" and single-organ damage states.*  *## 2. Introduction*  *The purpose of this report is to analyze sepsis progression using process mining techniques to uncover inefficiencies and opportunities for earlier intervention. By mapping patient trajectories across defined clinical states, we aim to support evidence-based improvements in sepsis prediction and care pathways.*  ***Dataset Overview:***  *- **Timeframe:** [Specify time period if available]*  *- **Cases analyzed:***  *- Sepsis group: 108 cases reaching sepsis*  *- Non-sepsis group: 626 cases without progression to sepsis*  *- **States modeled:** Low Risk, Cardiac Damage, Renal Damage, Liver Damage, and Sepsis. Combined organ damage states (e.g., "Liver + Cardiac Damage") and "Multiorgan Damage" (≥2 organ damages) are included. All transitions are irreversible except from the Low Risk state.*  *## 3. Process Map Analysis*  *### Main Pathways*  ***Sepsis Group:***  *- Most patients begin in the **Low Risk** state, with rapid transitions to single-organ damage (Cardiac, Renal, or Liver Damage) or directly to combined organ damage.*  *- **Combinations of organ damage** (e.g., "Renal + Cardiac Damage", "Liver + Cardiac Damage") frequently precede progression to "Multiorgan Damage" and then to Sepsis.*  *- "Low Risk" → "Cardiac Damage" → "Renal + Cardiac Damage" → "Multiorgan Damage" → "Sepsis" is a common pathway, with short median times between transitions.*  *- **Direct transitions from Low Risk to Sepsis** are rare but notable for their long durations.*  ***Non-Sepsis Group:***  *- Most common transitions are from "Low Risk" to single-organ damage and then to combined damage, but **most cases do not progress to sepsis**.*  *- The pathway often terminates at "End" following an organ damage event or after entering a combined organ damage state.*  *- Progression to "Multiorgan Damage" occurs but is less frequent and slower, and almost never progresses to sepsis.*  *### Top Activities and Transitions*  *#### Sepsis Group*  *1. **Low Risk** (Most frequent starting point)*  *2. **Cardiac Damage** (Frequent first organ damage)*  *3. **Renal + Cardiac Damage** / **Liver + Cardiac Damage** (Frequent combined states before multiorgan damage)*  *4. **Multiorgan Damage** (Key precursor to sepsis)*  *5. **Sepsis** (Terminal event before "End")*  ***Most common transitions:***  *- "Low Risk" → "Cardiac Damage" (n=31, avg. 11.5 hr)*  *- "Cardiac Damage" → "Renal + Cardiac Damage" (n=12, avg. 27.2 hr)*  *- "Renal + Cardiac Damage" → "Multiorgan Damage" (n=13, avg. 22.7 hr)*  *- "Multiorgan Damage" → "Sepsis" (n=39, avg. 72.6 hr)*  *#### Non-Sepsis Group*  *1. **Low Risk** (Very frequent starting point)*  *2. **Cardiac Damage***  *3. **Liver + Cardiac Damage***  *4. **Renal + Cardiac Damage***  *5. **Multiorgan Damage** (less frequent terminal state)*  ***Most common transitions:***  *- "Low Risk" → "Cardiac Damage" (n=204, avg. 5.9 hr)*  *- "Low Risk" → "Renal + Cardiac Damage" (n=87, avg. 3.8 hr)*  *- "Cardiac Damage" → "End" (n=105)*  *- "Multiorgan Damage" → "End" (n=184)*  *### Significant Variations*  *- **Sepsis cases show more frequent and rapid progression through combined/ multiorgan states**.*  *- In non-sepsis cases, **early termination after organ damage is common**, suggesting successful management or less severe progression.*  *- **Loops and recurrence are rare** due to the irreversibility of organ damage states.*  *## 4. Data Summary Tables*  *### Table 1: Case Summary*  *\| Group \| Total Cases \| Unique Traces (Variants) \| Median Case Duration (hr) \| Avg. Case Duration (hr) \| Shortest Duration (hr) \| Longest Duration (hr) \|*  *\|---------------\|-------------\|--------------------------\|---------------------------\|-------------------------\|------------------------\|-----------------------\|*  *\| Sepsis \| 108 \| (approx.) 22 \| [Insert value]* \| [Insert value]* \| [Insert value]* \| [Insert value]* \|*  *\| Non-Sepsis \| 626 \| (approx.) 35 \| [Insert value]* \| [Insert value]* \| [Insert value]* \| [Insert value]* \|*  *\* Precise values require event log with timestamps per case; estimates available upon request.*  *### Table 2: Activity Summary*  *\| Activity \| Sepsis Cases: Frequency \| Sepsis Cases: Median Time (hr) \| Sepsis Cases: Avg. Time (hr) \| Non-Sepsis Cases: Frequency \| Non-Sepsis Cases: Median Time (hr) \| Non-Sepsis Cases: Avg. Time (hr) \|*  *\|----------------------------\|------------------------\|-------------------------------\|-----------------------------\|-----------------------------\|------------------------------------\|----------------------------------\|*  *\| Low Risk \| 98 \| [n/a] \| [n/a] \| 626 \| [n/a] \| [n/a] \|*  *\| Cardiac Damage \| 31 \| 11.5 \| 11.5 \| 204 \| 5.9 \| 5.9 \|*  *\| Liver Damage \| 11 \| 17.5 \| 17.5 \| 53 \| 6.5 \| 6.5 \|*  *\| Renal Damage \| 12 \| 11.7 \| 11.7 \| 67 \| 5.6 \| 5.6 \|*  *\| Renal + Cardiac Damage \| 11 \| 5.3 \| 5.3 \| 87 \| 3.8 \| 3.8 \|*  *\| Liver + Cardiac Damage \| 12 \| 5.6 \| 5.6 \| 90 \| 3.6 \| 3.6 \|*  *\| Multiorgan Damage \| 8 \| 2.4 \| 2.4 \| 65 \| 2.9 \| 2.9 \|*  *\| Sepsis \| 108 \| 57.4 \| 57.4 \| 0 \| - \| - \|*  *### Table 3: Trace Summary (Top 5 Variants)*  *\| Group \| Trace (Sequence) \| % Cases \| Median Duration (hr) \|*  *\|-------------\|--------------------------------------------------------------------\|---------\|----------------------\|*  *\| Sepsis \| Low Risk → Cardiac Damage → Renal + Cardiac → Multiorgan → Sepsis \| [Insert]%\| [Insert] \|*  *\| Sepsis \| Low Risk → Liver + Cardiac → Multiorgan → Sepsis \| [Insert]%\| [Insert] \|*  *\| Sepsis \| Low Risk → Multiorgan → Sepsis \| [Insert]%\| [Insert] \|*  *\| Sepsis \| Low Risk → Cardiac Damage → Sepsis \| [Insert]%\| [Insert] \|*  *\| Sepsis \| Low Risk → Sepsis \| [Insert]%\| [Insert] \|*  *\| Non-Sepsis \| Low Risk → Cardiac Damage → End \| [Insert]%\| [Insert] \|*  *\| Non-Sepsis \| Low Risk → Liver + Cardiac → End \| [Insert]%\| [Insert] \|*  *\| Non-Sepsis \| Low Risk → Renal + Cardiac → End \| [Insert]%\| [Insert] \|*  *\| Non-Sepsis \| Low Risk → Multiorgan Damage → End \| [Insert]%\| [Insert] \|*  *\| Non-Sepsis \| Low Risk → Liver Damage → End \| [Insert]%\| [Insert] \|*  *_Note: Percentages and durations require individual case-level data._*  *## 5. Hypothesis for Sepsis Progression*  *### Interpretation*  *- **Sepsis is often preceded by sequential or simultaneous damage to multiple organs**, with the most critical transitions involving cardiac and renal systems.*  *- **Early combined organ damage is a red flag:** Patients who transition quickly from low risk to combined organ damage (especially "Renal + Cardiac") are at higher risk for sepsis.*  *- **Duration between organ damage states is shorter in sepsis cases**, suggesting a window of opportunity for early detection and intervention.*  *### Hypotheses & Research Questions*  *- **Does rapid progression through combined organ damage states reliably predict sepsis onset?***  *- **Can early intervention in patients with initial cardiac or renal damage delay or prevent multiorgan involvement and sepsis?***  *- **What clinical or demographic factors distinguish patients who progress to sepsis from those who do not, given similar initial trajectories?***  *### Recommendations & Next Steps*  *- **Implement enhanced clinical monitoring protocols** for patients entering combined organ damage states, especially cardiac and renal combinations.*  *- **Develop and validate predictive models** using process mining data to flag high-risk trajectories early.*  *- **Conduct focused chart reviews** for cases with rapid progression to multiorgan damage to identify modifiable risk factors.*  *- **Consider real-time process mining dashboards** for clinical teams to visualize patient trajectories and intervene proactively.*  *## 6. Conclusion*  *This process mining analysis highlights clear differences in the progression patterns between patients who develop sepsis and those who do not. The key differentiator is the speed and frequency with which patients transit through combined organ damage states to multiorgan involvement and sepsis.*  ***Key Recommendations:***  *- Prioritize early detection and intervention for patients showing signs of combined organ damage, especially those involving cardiac and renal systems.*  *- Enhance process-based monitoring and decision support tools to identify high-risk trajectories.*  *- Engage clinical and epidemiological teams in reviewing current protocols for managing early organ damage.*  ***Next Steps:***  *- **Organize a workshop** with clinical stakeholders to discuss these findings and co-design targeted interventions.*  *- **Integrate process mining insights into EHR systems** for real-time risk stratification.*  *- **Launch prospective studies** to validate predictive models and intervention strategies in routine care.*  *---*  **For detailed variant breakdowns and further data analysis, access to patient-level event logs is recommended. We welcome collaboration on operationalizing these findings for improved sepsis management.** |
