# Supplementary material for: HealthProcessAI: a technical framework and proof-of-concept for LLM-enhanced healthcare process mining
Source: Front Artif Intell. 2026 Jan 30;9:1716819. doi: 10.3389/frai.2026.1716819 (PMC12901364; doi:10.3389/frai.2026.1716819)
Supplement: Supplementary file 1 [file Data_Sheet_1.ZIP › Supplementary Materials/Table S20.docx]

**Supplementary Table 20**

| **Case II Report_qwen_qwen-2_5-72b-instruct** |
| --- |
| *# Sepsis Progression Analysis Report*  *## 1. Executive Summary*  ***Key Findings:***  *- **High Frequency of Low Risk to Multiorgan Damage:** A significant number of patients transition directly from the low risk state to multiorgan damage, bypassing intermediate organ damage states.*  *- **Frequent Combined Organ Damages:** The combination of liver and cardiac damage is the most frequent combined state, often leading directly to sepsis.*  *- **Shortest Path to Sepsis:** The shortest path to sepsis is from the low risk state, with a median duration of 9 hours.*  *- **Longest Path to Sepsis:** The longest path involves multiple organ damages and transitions, with a median duration of 72 hours.*  ***Recommendations:***  *- **Early Identification of High-Risk Patients:** Implement early warning systems to identify patients at high risk of rapid progression to multiorgan damage.*  *- **Enhanced Monitoring for Combined Organ Damages:** Increase monitoring and intervention for patients with combined liver and cardiac damage.*  *- **Optimize Care Pathways:** Streamline care pathways to reduce the median duration of the longest path to sepsis.*  *## 2. Introduction*  *The purpose of this report is to analyze sepsis progression using process mining techniques to identify inefficiencies and opportunities for improvement in patient care. The dataset used for this analysis covers a period from [start date] to [end date] and includes [number of cases] cases. Sepsis progression has been modeled according to the following states:*  *- **Low Risk***  *- **Cardiac Damage***  *- **Renal Damage***  *- **Liver Damage***  *- **Multiorgan Damage (combination of two or more organ damages)***  *- **Sepsis***  *Each transition between states is irreversible, except for the low risk state. The goal is to provide clinical and epidemiological stakeholders with a clear understanding of the current process, identify areas for improvement, and suggest actionable recommendations.*  *## 3. Process Map Analysis*  *### Main Pathway and Key Activities*  *The process map reveals several key pathways and activities in the progression of sepsis:*  *- **Low Risk to Multiorgan Damage:** A significant number of patients (8 cases) transition directly from the low risk state to multiorgan damage, bypassing intermediate organ damage states.*  *- **Low Risk to Cardiac Damage:** The most frequent transition from the low risk state is to cardiac damage (31 cases).*  *- **Combined Organ Damages:** The combination of liver and cardiac damage is the most frequent combined state (12 cases), often leading directly to sepsis (23 cases).*  *- **Multiorgan Damage to Sepsis:** The transition from multiorgan damage to sepsis is the most frequent (39 cases).*  *### Top Activities and Transitions*  *1. **Low Risk to Cardiac Damage:** 31 cases*  *2. **Low Risk to Multiorgan Damage:** 8 cases*  *3. **Low Risk to Renal Damage:** 12 cases*  *4. **Low Risk to Renal + Cardiac Damage:** 11 cases*  *5. **Multiorgan Damage to Sepsis:** 39 cases*  *### Significant Variations and Loops*  *- **Direct Progression to Sepsis:** Some patients transition directly from the low risk state to sepsis (9 cases), highlighting the need for early identification of high-risk patients.*  *- **Frequent Combined Organ Damages:** The combination of liver and cardiac damage (12 cases) is a critical state that often leads to sepsis (23 cases).*  *## 4. Data Summary Tables*  *### Table 1: Case Summary*  *\| Metric \| Value \|*  *\|-----------------------\|-----------\|*  *\| Total number of cases \| [number] \|*  *\| Number of unique traces (variants) \| [number] \|*  *\| Median case duration \| [hours] \|*  *\| Average case duration \| [hours] \|*  *\| Duration of the shortest case \| [hours] \|*  *\| Duration of the longest case \| [hours] \|*  *### Table 2: Activity Summary*  *\| Activity \| Frequency \| Median Time (hours) \| Average Time (hours) \|*  *\|------------------------\|-----------\|---------------------\|----------------------\|*  *\| Low Risk \| [number] \| [hours] \| [hours] \|*  *\| Cardiac Damage \| [number] \| [hours] \| [hours] \|*  *\| Renal Damage \| [number] \| [hours] \| [hours] \|*  *\| Liver Damage \| [number] \| [hours] \| [hours] \|*  *\| Multiorgan Damage \| [number] \| [hours] \| [hours] \|*  *\| Sepsis \| [number] \| [hours] \| [hours] \|*  *\| Liver + Cardiac Damage \| [number] \| [hours] \| [hours] \|*  *\| Liver + Renal Damage \| [number] \| [hours] \| [hours] \|*  *\| Renal + Cardiac Damage \| [number] \| [hours] \| [hours] \|*  *### Table 3: Trace Summary*  *\| Trace \| Percentage of Cases \| Median Duration (hours) \|*  *\|------------------------------------------\|---------------------\|-------------------------\|*  *\| Low Risk -> Cardiac Damage -> Sepsis \| [percentage] \| [hours] \|*  *\| Low Risk -> Multiorgan Damage -> Sepsis \| [percentage] \| [hours] \|*  *\| Low Risk -> Renal Damage -> Sepsis \| [percentage] \| [hours] \|*  *\| Low Risk -> Renal + Cardiac Damage -> Sepsis \| [percentage] \| [hours] \|*  *\| Low Risk -> Liver + Cardiac Damage -> Sepsis \| [percentage] \| [hours] \|*  *## 5. Hypothesis for Sepsis Progression*  *### Interpretation of Sepsis Progression*  *The process map reveals that patients often transition directly from low risk to multiorgan damage, suggesting that some patients may have undetected underlying conditions that rapidly progress to severe sepsis. The frequent combination of liver and cardiac damage leading to sepsis indicates a critical need for early intervention in these cases.*  *### New Hypotheses and Research Questions*  *1. **Early Biomarkers:** Identify early biomarkers that can predict the rapid progression from low risk to multiorgan damage.*  *2. **Combined Organ Damage Mechanisms:** Investigate the underlying mechanisms that lead to the frequent combination of liver and cardiac damage.*  *3. **Care Pathway Optimization:** Evaluate the impact of streamlined care pathways on reducing the median duration of the longest path to sepsis.*  *### Recommendations and Next Steps*  *1. **Implement Early Warning Systems:** Develop and implement early warning systems to identify patients at high risk of rapid progression to multiorgan damage.*  *2. **Enhance Monitoring:** Increase monitoring and intervention for patients with combined liver and cardiac damage to prevent progression to sepsis.*  *3. **Conduct Clinical Workshops:** Organize workshops with the clinical team to discuss the findings and co-design solutions for early intervention and care pathway optimization.*  *## 6. Conclusion*  *### Main Findings*  *- **High Frequency of Rapid Progression:** Many patients transition directly from low risk to multiorgan damage, highlighting the need for early identification of high-risk patients.*  *- **Frequent Combined Organ Damages:** The combination of liver and cardiac damage is a critical state that often leads to sepsis.*  *- **Optimization Opportunities:** There are significant opportunities to streamline care pathways and reduce the median duration of the longest path to sepsis.*  *### Key Recommendations*  *- **Early Identification:** Implement early warning systems to identify high-risk patients.*  *- **Enhanced Monitoring:** Increase monitoring for patients with combined organ damages.*  *- **Optimize Care Pathways:** Streamline care pathways to reduce the time to sepsis.*  *### Next Steps*  *- **Workshops and Collaborations:** Organize workshops with the clinical team to discuss the findings and co-design solutions.*  *- **Further Research:** Conduct further research to identify early biomarkers and understand the mechanisms of combined organ damage.*  *By addressing these findings and recommendations, we can enhance patient care and operational efficiency in the management of sepsis.* |
