# Supplementary material for: HealthProcessAI: a technical framework and proof-of-concept for LLM-enhanced healthcare process mining
Source: Front Artif Intell. 2026 Jan 30;9:1716819. doi: 10.3389/frai.2026.1716819 (PMC12901364; doi:10.3389/frai.2026.1716819)
Supplement: Supplementary file 1 [file Data_Sheet_1.ZIP › Supplementary Materials/Table S21.docx]

**Supplementary Table 21**

| **Case II Report_google_gemma-2-27b-it** |
| --- |
| *# Report on Sepsis Progression Analysis using Process Mining*  *## Executive Summary*  *This report analyzes sepsis progression using process mining techniques. The analysis reveals several key findings regarding the pathways of sepsis development, highlighting areas for improvement in patient care and operational efficiency.*  ** **Dominant Pathway:** The most frequent pathway observed involves progression from "Low Risk" to "Cardiac Damage," often followed by "Multiorgan Damage" and ultimately "Sepsis."*  ** **Significant Variations:** While the dominant pathway is evident, there are notable variations in sepsis progression. For instance, direct transitions from "Low Risk" to "Sepsis" occur, indicating potential for earlier detection.*  ** **Opportunities for Improvement:** The analysis suggests several opportunities for intervention. This includes:*  ** **Early Detection:** Investigating the factors leading to direct transitions to "Sepsis" from "Low Risk" to potentially develop early warning systems.*  ** **Targeted Interventions:** Understanding the characteristics of patients progressing through different pathways (e.g., "Multiorgan Damage") to allow for tailored interventions and potentially improve outcomes.*  ** **Process Standardization:** Exploring the possibility of standardizing certain stages of the process (e.g., the transition from "Multiorgan Damage" to "Sepsis") to enhance consistency in care.*  *## Introduction*  *This report aims to analyze sepsis progression using process mining techniques to identify inefficiencies and opportunities for improvement in patient care. The dataset used for the analysis encompasses [**Insert time frame of data here**] and includes [**Insert total number of cases analyzed here**] cases of sepsis.*  *We modeled sepsis progression according to the following states:*  ** **Low Risk:** Initial stage where patients are at risk for developing sepsis.*  ** **Cardiac Damage:** Damage to the heart.*  ** **Renal Damage:** Damage to the kidneys.*  ** **Liver Damage:** Damage to the liver.*  ** **Multiorgan Damage:** Simultaneous damage to two or more organs.*  ** **Sepsis:** The clinical syndrome characterized by systemic inflammation and organ dysfunction.*  *It is crucial to note that two organ damages can be combined in a specific state (e.g., Cardiac Damage + Liver Damage). However, the combination of two or more organ damages leads to "Multiorgan Damage" state.*  *All transitions are irreversible, except for the transition from "Low Risk" to other states.*  *## Process Map Analysis*  *The analysis reveals a dominant pathway for sepsis progression, with several variations and interesting observations:*  ** **Dominant Pathway:** The most common pathway involves the progression from "Low Risk" to "Cardiac Damage," followed by "Multiorgan Damage" and finally "Sepsis." This pathway highlights the typical progression of sepsis development.*  ** **Frequent Activities:***  ** **Low Risk:** This is the starting point for most cases, indicating the importance of early detection and monitoring.*  ** **Cardiac Damage:** A frequent early indicator of developing sepsis, often preceding further organ damage.*  ** **Multiorgan Damage:** This stage signifies a critical point in the progression, often leading to "Sepsis."*  ** **Sepsis:** The ultimate outcome in this analysis, representing the serious clinical syndrome.*  ** **Key Transitions:***  ** **Low Risk to Cardiac Damage:** This transition is the most frequent, emphasizing the need for early interventions to prevent further damage.*  ** **Cardiac Damage to Multiorgan Damage:** This transition indicates the progression towards more severe organ dysfunction.*  ** **Variations from Expected Progression:***  ** **Direct Transitions to Sepsis:** Notably, there are cases where patients transition directly from "Low Risk" to "Sepsis," suggesting potential for earlier detection and intervention.*  *## Data Summary Tables*  *### Table 1: Case Summary*  *\| Metric \| Value \|*  *\|---\|---\|*  *\| Total Number of Cases \| [Insert total number of cases here] \|*  *\| Number of Unique Traces (Variants) \| [Insert number of unique traces here] \|*  *\| Median Case Duration (hours) \| [Insert median duration here] \|*  *\| Average Case Duration (hours) \| [Insert average duration here] \|*  *\| Duration of Shortest Case (hours) \| [Insert shortest duration here] \|*  *\| Duration of Longest Case (hours) \| [Insert longest duration here] \|*  *### Table 2: Activity Summary*  *\| Activity \| Frequency \| Median Time Spent (hours) \| Average Time Spent (hours) \|*  *\|---\|---\|---\|---\|*  *\| Low Risk \| [Insert frequency here] \| [Insert median time here] \| [Insert average time here] \|*  *\| Cardiac Damage \| [Insert frequency here] \| [Insert median time here] \| [Insert average time here] \|*  *\| Multiorgan Damage \| [Insert frequency here] \| [Insert median time here] \| [Insert average time here] \|*  *\| Sepsis \| [Insert frequency here] \| [Insert median time here] \| [Insert average time here] \|*  *\| Liver Damage \| [Insert frequency here] \| [Insert median time here] \| [Insert average time here] \|*  *\| Liver + Renal Damage \| [Insert frequency here] \| [Insert median time here] \| [Insert average time here] \|*  *\| Renal Damage \| [Insert frequency here] \| [Insert median time here] \| [Insert average time here] \|*  *\| Renal + Cardiac Damage \| [Insert frequency here] \| [Insert median time here] \| [Insert average time here] \|*  *\| Liver + Cardiac Damage \| [Insert frequency here] \| [Insert median time here] \| [Insert average time here] \|*  *### Table 3: Trace Summary*  *\| Trace \| Percentage of Cases \| Median Duration (hours) \|*  *\|---\|---\|---\|*  *\| [Insert most frequent trace here] \| [Insert percentage here] \| [Insert median duration here] \|*  *\| [Insert second most frequent trace here] \| [Insert percentage here] \| [Insert median duration here] \|*  *\| [Insert third most frequent trace here] \| [Insert percentage here] \| [Insert median duration here] \|*  *\| [Insert fourth most frequent trace here] \| [Insert percentage here] \| [Insert median duration here] \|*  *\| [Insert fifth most frequent trace here] \| [Insert percentage here] \| [Insert median duration here] \|*  *## Hypothesis for Sepsis Progression*  *This analysis provides valuable insights into sepsis progression, leading to several hypotheses and research questions:*  ** **Early Detection Hypothesis:** The presence of direct transitions from "Low Risk" to "Sepsis" suggests the possibility of developing predictive models to identify patients at high risk for rapid sepsis development.*  ** **Multiorgan Damage Hypothesis:** Understanding the characteristics of patients progressing through the "Multiorgan Damage" state could lead to targeted interventions to prevent further deterioration.*  ***Research Questions:***  ** Can we identify specific biomarkers or clinical factors that predict direct transitions from "Low Risk" to "Sepsis"?*  ** Are there distinct patient profiles associated with different pathways of sepsis progression (e.g., those with "Multiorgan Damage" versus those with direct transitions to "Sepsis")?*  ** Can we develop standardized protocols for managing patients with "Multiorgan Damage" to improve outcomes?*  *## Conclusion*  *This analysis provides a comprehensive overview of sepsis progression using process mining techniques. The identified dominant pathway, variations, and key transitions offer valuable insights into the clinical course of sepsis. The identified hypotheses and research questions provide a clear direction for future studies aimed at improving patient care and operational efficiency.*  ***Next Steps:***  ** **Workshop with Clinical Team:** A workshop with the clinical team is recommended to discuss the findings of this report and co-design solutions based on the identified areas for improvement.*  ** **Develop Predictive Models:** Explore the development of predictive models to identify patients at high risk for rapid sepsis development.*  ** **Targeted Intervention Strategies:** Investigate the development of targeted intervention strategies based on the different pathways of sepsis progression.* |
