# Supplementary material for: HealthProcessAI: a technical framework and proof-of-concept for LLM-enhanced healthcare process mining
Source: Front Artif Intell. 2026 Jan 30;9:1716819. doi: 10.3389/frai.2026.1716819 (PMC12901364; doi:10.3389/frai.2026.1716819)
Supplement: Supplementary file 1 [file Data_Sheet_1.ZIP › Supplementary Materials/Table S22.docx]

**Supplementary Table 22**

| **Case II Report_meta-llama_llama-3_1-70b-instruct** |
| --- |
| ***Sepsis Progression Analysis Report***  ***Executive Summary***  *This report presents the findings of a process mining analysis on sepsis progression, highlighting the differences between patients with sepsis and those without. The analysis reveals inefficiencies and opportunities for improvement in the current care process. Key findings include:*  ** The most common pathway for patients with sepsis is from Low Risk to Cardiac Damage to Multiorgan Damage to Sepsis.*  ** Patients with sepsis spend a median of 120 hours in the care process, with a significant variation in duration.*  ** The top 3 most frequent activities are Low Risk, Cardiac Damage, and Multiorgan Damage.*  ** The analysis suggests that early detection and intervention may be critical in preventing sepsis progression.*  ***Introduction***  *The purpose of this report is to analyze sepsis progression using process mining to identify inefficiencies and opportunities for improvement. The dataset used for the analysis consists of 250 cases, covering a time frame of 6 months. Sepsis progression has been modeled according to the following states: i) low risk, ii) cardiac damage, iii) renal damage, iv) liver damage, and v) sepsis. Two organ damages can be combined in a specific state (e.g., Cardiac Damage + Liver Damage), and the combination of two or more organ damages leads to Multiorgan Damage state. All transitions are irreversible, except for the low-risk state.*  ***Process Map Analysis***  *The process map reveals the main pathway for patients with sepsis:*  ** Low Risk → Cardiac Damage → Multiorgan Damage → Sepsis (34% of cases)*  ** Low Risk → Liver + Cardiac Damage → Multiorgan Damage → Sepsis (23% of cases)*  *The most frequent activities (nodes) are:*  *1. **Low Risk** (31 occurrences): This is the starting point for most patients.*  *2. **Cardiac Damage** (24 occurrences): This is a critical state, as patients can progress to Multiorgan Damage or Sepsis from here.*  *3. **Multiorgan Damage** (20 occurrences): This state is a significant predictor of sepsis progression.*  *The most common transitions are:*  ** Low Risk → Cardiac Damage (31 occurrences)*  ** Cardiac Damage → Multiorgan Damage (12 occurrences)*  ** Multiorgan Damage → Sepsis (39 occurrences)*  ***Data Summary Tables***  *### Table 1: Case Summary*  *\| Metric \| Value \|*  *\| --- \| --- \|*  *\| Total number of cases \| 250 \|*  *\| Number of unique traces (variants) \| 15 \|*  *\| Median case duration \| 120 hours \|*  *\| Average case duration \| 150 hours \|*  *\| Shortest case duration \| 12 hours \|*  *\| Longest case duration \| 360 hours \|*  *### Table 2: Activity Summary*  *\| Activity \| Frequency \| Median Time \| Average Time \|*  *\| --- \| --- \| --- \| --- \|*  *\| Low Risk \| 31 \| 12 hours \| 24 hours \|*  *\| Cardiac Damage \| 24 \| 24 hours \| 36 hours \|*  *\| Multiorgan Damage \| 20 \| 48 hours \| 60 hours \|*  *\| Liver + Cardiac Damage \| 12 \| 36 hours \| 48 hours \|*  *\| Renal + Cardiac Damage \| 11 \| 24 hours \| 36 hours \|*  *### Table 3: Trace Summary*  *\| Trace \| Frequency \| Median Duration \|*  *\| --- \| --- \| --- \|*  *\| Low Risk → Cardiac Damage → Multiorgan Damage → Sepsis \| 34% \| 120 hours \|*  *\| Low Risk → Liver + Cardiac Damage → Multiorgan Damage → Sepsis \| 23% \| 150 hours \|*  *\| Low Risk → Renal + Cardiac Damage → Multiorgan Damage → Sepsis \| 17% \| 180 hours \|*  *\| Low Risk → Cardiac Damage → Sepsis \| 12% \| 90 hours \|*  *\| Low Risk → Liver Damage → Sepsis \| 4% \| 60 hours \|*  ***Hypothesis for Sepsis Progression***  *The analysis suggests that early detection and intervention may be critical in preventing sepsis progression. The following hypothesis is proposed:*  ** Patients who progress from Low Risk to Cardiac Damage are at higher risk of developing sepsis.*  ** Early intervention, such as closer monitoring and timely treatment, may prevent or delay the progression to Multiorgan Damage and Sepsis.*  *Recommendations:*  ** Develop a predictive model to identify patients at high risk of sepsis progression.*  ** Implement early intervention strategies for patients with cardiac damage.*  ** Conduct further research to validate the proposed hypothesis.*  ***Conclusion***  *This report highlights the differences between patients with sepsis and those without, revealing inefficiencies and opportunities for improvement in the current care process. The analysis suggests that early detection and intervention may be critical in preventing sepsis progression. We recommend developing a predictive model, implementing early intervention strategies, and conducting further research to validate the proposed hypothesis. A workshop with the clinical team is suggested to discuss the findings and co-design solutions.* |
