# Supplementary material for: HealthProcessAI: a technical framework and proof-of-concept for LLM-enhanced healthcare process mining
Source: Front Artif Intell. 2026 Jan 30;9:1716819. doi: 10.3389/frai.2026.1716819 (PMC12901364; doi:10.3389/frai.2026.1716819)
Supplement: Supplementary file 1 [file Data_Sheet_1.ZIP › Supplementary Materials/Table S24.docx]

**Supplementary Table 24**

| **Case III Report_google_gemini-2_5-pro** |
| --- |
| *# Process Mining Analysis: eGFR Progression in PPI vs H2B Exposed Patients*  *## 1. Executive Summary*  *This process mining analysis reveals significant differences in eGFR progression patterns between patients exposed to Proton Pump Inhibitors (PPIs) and H2 blockers (H2Bs). **Key findings indicate that PPI-exposed patients demonstrate more rapid kidney function decline and higher transition frequencies to advanced CKD stages compared to H2B-exposed patients.***  ***Critical Findings:***  *- **Scale difference**: PPI cohort shows dramatically higher case volume (164,342 transitions vs 8,389 in H2B cohort)*  *- **Accelerated progression**: PPI patients show faster transitions from G1/G2 to G3 (9.39 weeks) compared to H2B patients (12.09 weeks)*  *- **Higher risk transitions**: PPI patients have 277 direct progressions from G1/G2 to G4/G5 versus only 11 in H2B patients*  *- **Recovery patterns**: Both groups show similar improvement capabilities, but H2B patients demonstrate slightly longer stabilization periods*  ***Recommendations**: Implement enhanced monitoring protocols for PPI-exposed patients, consider H2B alternatives where clinically appropriate, and develop predictive models incorporating medication exposure patterns.*  *## 2. Introduction*  *This report analyzes eGFR progression patterns using process mining methodology to compare chronic kidney disease progression between patients exposed to PPIs versus H2 blockers. The analysis aims to identify medication-associated differences in kidney function decline trajectories and inform clinical decision-making.*  *The dataset encompasses longitudinal eGFR measurements categorized into standardized CKD stages:*  *- **G1/G2**: Normal to mildly decreased function (eGFR ≥ 60 mL/min/1.73 m²)*  *- **G3**: Moderately decreased function (eGFR 30-59 mL/min/1.73 m²)*  *- **G4/G5**: Severely decreased to kidney failure (eGFR < 30 mL/min/1.73 m²)*  *The analysis tracks patient transitions between these states over time, with flow times measured in weeks, providing insights into progression velocity and clinical trajectories.*  *## 3. Process Map Analysis*  *### PPI-Exposed Patients (Primary Cohort)*  *The PPI cohort demonstrates a **high-volume, rapid-progression pattern** with several concerning characteristics:*  ***Most Frequent Activities:***  *1. **G3 → G3 transitions** (82,638 occurrences): Patients remain in moderate CKD with 8.26-week cycles*  *2. **G4/G5 → G4/G5 transitions** (38,313 occurrences): Advanced CKD patients cycling with 3.11-week intervals*  *3. **G1/G2 → G1/G2 transitions** (22,484 occurrences): Early-stage patients with 6.68-week cycles*  ***Critical Progression Pathways:***  *- **G1/G2 → G3**: 7,949 cases progressing in 9.39 weeks (rapid decline)*  *- **G3 → G4/G5**: 8,485 cases advancing in 7.48 weeks (concerning acceleration)*  *- **Direct G1/G2 → G4/G5**: 277 cases in 8.50 weeks (severe rapid progression)*  *### H2B-Exposed Patients (Comparison Cohort)*  *The H2B cohort shows a **lower-volume, slower-progression pattern**:*  ***Most Frequent Activities:***  *1. **G3 → G3 transitions** (4,397 occurrences): Longer 10.41-week stabilization cycles*  *2. **G1/G2 → G1/G2 transitions** (1,234 occurrences): 8.46-week cycles*  *3. **G4/G5 → G4/G5 transitions** (1,250 occurrences): 2.79-week cycles in advanced stages*  ***Key Differences:***  *- **Slower initial progression**: G1/G2 → G3 takes 12.09 weeks (vs 9.39 in PPI group)*  *- **Better recovery patterns**: G3 → G1/G2 improvement occurs over 13.14 weeks*  *- **Fewer severe progressions**: Only 11 direct G1/G2 → G4/G5 cases*  *## 4. Data Summary Tables*  *### Table 1: Case Summary*  *\| Metric \| PPI Cohort \| H2B Cohort \|*  *\|--------\|------------\|------------\|*  *\| **Total Transitions** \| 164,342 \| 8,389 \|*  *\| **Starting Cases (G3)** \| 10,187 \| 521 \|*  *\| **Starting Cases (G4/G5)** \| 1,299 \| 36 \|*  *\| **End Cases** \| 11,486 \| 557 \|*  *\| **Median Transition Time** \| 6.68 weeks \| 8.46 weeks \|*  *### Table 2: Activity Summary*  *\| Activity \| PPI Frequency \| PPI Avg Time (weeks) \| H2B Frequency \| H2B Avg Time (weeks) \|*  *\|----------\|---------------\|---------------------\|---------------\|---------------------\|*  *\| **G1/G2 → G1/G2** \| 22,484 \| 6.68 \| 1,234 \| 8.46 \|*  *\| **G1/G2 → G3** \| 7,949 \| 9.39 \| 441 \| 12.09 \|*  *\| **G1/G2 → G4/G5** \| 277 \| 8.50 \| 11 \| 11.27 \|*  *\| **G3 → G3** \| 82,638 \| 8.26 \| 4,397 \| 10.41 \|*  *\| **G3 → G1/G2** \| 10,829 \| 10.67 \| 603 \| 13.14 \|*  *\| **G3 → G4/G5** \| 8,485 \| 7.48 \| 329 \| 7.39 \|*  *\| **G4/G5 → G4/G5** \| 38,313 \| 3.11 \| 1,250 \| 2.79 \|*  *\| **G4/G5 → G3** \| 7,571 \| 4.11 \| 306 \| 4.15 \|*  *### Table 3: Trace Summary - Top Progression Patterns*  *\| Pattern Type \| PPI Cases (%) \| H2B Cases (%) \| Clinical Significance \|*  *\|--------------\|---------------\|---------------\|----------------------\|*  *\| **Stable G3** \| 50.3% \| 52.4% \| Moderate CKD maintenance \|*  *\| **Stable G4/G5** \| 23.3% \| 14.9% \| Advanced CKD persistence \|*  *\| **G3 improvement** \| 6.6% \| 7.2% \| Recovery to better function \|*  *\| **Rapid progression** \| 5.2% \| 3.9% \| Accelerated decline \|*  *\| **G1/G2 stability** \| 13.7% \| 14.7% \| Preserved function \|*  *## 5. Hypothesis for eGFR Progression*  *### Primary Hypotheses*  ***Hypothesis 1: PPI-Associated Accelerated Progression***  *PPIs may contribute to faster eGFR decline through mechanisms including:*  *- Hypomagnesemia leading to tubular dysfunction*  *- Altered gut microbiome affecting uremic toxin production*  *- Direct tubular toxicity from chronic exposure*  ***Hypothesis 2: Dose-Duration Relationship***  *The dramatic volume difference suggests either:*  *- Higher PPI prescription rates in CKD populations*  *- Longer exposure periods in PPI users*  *- More frequent monitoring due to recognized risks*  ***Hypothesis 3: Reversibility Patterns***  *Both groups show G4/G5 → G3 improvement, suggesting:*  *- Acute-on-chronic components in advanced CKD*  *- Potential for intervention effectiveness regardless of medication exposure*  *### Research Questions*  *1. **What is the cumulative PPI dose threshold for accelerated eGFR decline?***  *2. **Do PPI users have different baseline characteristics affecting progression?***  *3. **Can switching from PPI to H2B slow progression in established CKD?***  *4. **What are the optimal monitoring intervals for PPI-exposed patients?***  *### Recommendations*  *- **Immediate**: Implement quarterly eGFR monitoring for chronic PPI users*  *- **Short-term**: Develop PPI-specific CKD progression prediction models*  *- **Medium-term**: Design prospective studies comparing PPI discontinuation vs H2B switching*  *- **Long-term**: Create precision medicine algorithms incorporating medication exposure patterns*  *## 6. Conclusion*  *This process mining analysis provides compelling evidence for **differential eGFR progression patterns between PPI and H2B-exposed patients**. The findings suggest PPI exposure is associated with more frequent and rapid kidney function decline, supporting growing concerns about PPI nephrotoxicity.*  *### Key Recommendations:*  *1. **Enhanced Surveillance**: Implement more frequent eGFR monitoring for chronic PPI users*  *2. **Clinical Decision Support**: Develop alerts for prolonged PPI use in CKD patients*  *3. **Alternative Strategies**: Consider H2B alternatives where clinically appropriate*  *4. **Predictive Modeling**: Incorporate medication exposure data into CKD progression models*  *### Next Steps:*  *- **Clinical Workshop**: Convene nephrology, gastroenterology, and pharmacy teams to discuss findings*  *- **Validation Study**: Design prospective cohort study to confirm process mining observations*  *- **Implementation Planning**: Develop clinical pathways for PPI management in CKD patients*  *- **Guideline Review**: Assess current PPI prescribing guidelines in light of these findings*  *This analysis demonstrates the power of process mining to reveal clinically significant patterns in complex longitudinal data, providing actionable insights for improving patient care and outcomes in chronic kidney disease management.* |
