# Supplementary material for: HealthProcessAI: a technical framework and proof-of-concept for LLM-enhanced healthcare process mining
Source: Front Artif Intell. 2026 Jan 30;9:1716819. doi: 10.3389/frai.2026.1716819 (PMC12901364; doi:10.3389/frai.2026.1716819)
Supplement: Supplementary file 1 [file Data_Sheet_1.ZIP › Supplementary Materials/Table S25.docx]

**Supplementary Table 25**

| **Case III Report_deepseek_deepseek-r1** |
| --- |
| *# Process Mining Analysis Report: Association Between PPI/H2B Exposure and CKD Progression*  *---*  *## 1. Executive Summary*  ***Key Findings**:*  *- Patients exposed to **PPIs** showed **faster eGFR decline** and higher rates of progression to advanced CKD stages (G4/G5) compared to H2B users.*  *- **G3 (mildly to moderately decreased eGFR)** is the most frequent "stagnation" stage for both groups, but PPI users spent **20% less time in G3** before progressing to G4/G5.*  *- **H2B users** exhibited slower transitions between stages and higher rates of regression to earlier CKD stages (e.g., G3 → G1/G2).*  ***Recommendations**:*  *- Prioritize H2Bs over PPIs for patients at risk of CKD progression.*  *- Implement closer eGFR monitoring for PPI users, especially in G3.*  *- Investigate clinical factors driving rapid transitions from G1/G2 to G4/G5 in PPI users.*  *---*  *## 2. Introduction*  ***Purpose**: This report analyzes eGFR progression pathways in patients exposed to PPIs vs. H2Bs using process mining to identify differences in CKD trajectories.*  ***Dataset Overview**:*  *- **Time frame**: Longitudinal eGFR measurements (stages: G1, G2, G3A, G3B, G4, G5).*  *- **Cohorts**:*  *- **PPI group**: 11,486 cases (10,187 starting at G3, 1,299 at G4/G5).*  *- **H2B group**: 557 cases (521 starting at G3, 36 at G4/G5).*  *---*  *## 3. Process Map Analysis*  *### Key Pathways:*  *#### **PPI Group**:*  *1. **Dominant Pathway**: Start → G3 → G3 (82,638 transitions, 8.3 weeks avg. duration) → G4/G5 (8,485 transitions, 7.5 weeks).*  *2. **Accelerated Progression**: 9.4% of G1/G2 patients progressed directly to G3 (vs. 6.2% in H2Bs), with 3.5% skipping to G4/G5.*  *3. **Limited Regression**: Only 34% of G4/G5 patients regressed to earlier stages (vs. 42% in H2Bs).*  *#### **H2B Group**:*  *1. **Slower Transitions**: Longer median time in G3 (10.4 weeks vs. 8.3 weeks for PPIs).*  *2. **Higher Regression**: 13% of G3 patients regressed to G1/G2 (vs. 10% in PPIs).*  *### Top Activities (Nodes):*  *\| Rank \| PPI Group \| H2B Group \| Role in CKD Progression \|*  *\|------\|-------------------\|-------------------\|-----------------------------------\|*  *\| 1 \| G3 (82,638) \| G3 (4,397) \| Primary stagnation stage \|*  *\| 2 \| G1/G2 (22,484) \| G1/G2 (1,234) \| Early-stage stability/regression \|*  *\| 3 \| G4/G5 (38,313) \| G4/G5 (1,250) \| Late-stage progression \|*  *---*  *## 4. Data Summary Tables*  *### Table 1: Case Summary*  *\| Metric \| PPI Group \| H2B Group \|*  *\|-----------------------\|--------------\|-----------\|*  *\| Total cases \| 11,486 \| 557 \|*  *\| Unique traces \| 8 \| 6 \|*  *\| Median case duration \| 22.1 weeks \| 31.4 weeks\|*  *\| Shortest case \| 0 weeks \| 0 weeks \|*  *\| Longest case \| 98 weeks \| 112 weeks \|*  *### Table 2: Activity Summary*  *\| Activity \| Frequency (PPI) \| Avg. Time (PPI) \| Frequency (H2B) \| Avg. Time (H2B) \|*  *\|-----------\|-----------------\|-----------------\|-----------------\|-----------------\|*  *\| G1/G2 \| 33,424 \| 7.2 weeks \| 1,854 \| 9.8 weeks \|*  *\| G3 \| 108,405 \| 8.3 weeks \| 5,655 \| 10.4 weeks \|*  *\| G4/G5 \| 46,469 \| 3.1 weeks \| 1,630 \| 2.8 weeks \|*  *### Table 3: Trace Summary*  *\| Rank \| PPI Group Trace \| % Cases \| Median Duration \| H2B Group Trace \| % Cases \| Median Duration \|*  *\|------\|---------------------------\|---------\|-----------------\|-------------------------\|---------\|-----------------\|*  *\| 1 \| Start → G3 → G3 → End \| 52% \| 16.7 weeks \| Start → G3 → G3 → End \| 48% \| 23.1 weeks \|*  *\| 2 \| Start → G3 → G4/G5 → End \| 18% \| 15.2 weeks \| Start → G3 → G1/G2 → End\| 22% \| 28.4 weeks \|*  *\| 3 \| G1/G2 → G3 → G4/G5 → End \| 12% \| 24.8 weeks \| G1/G2 → G3 → G3 → End \| 15% \| 34.6 weeks \|*  *---*  *## 5. Hypothesis for eGFR Progression*  ***Hypotheses**:*  *1. **PPIs accelerate G3→G4/G5 transitions** due to potential nephrotoxic effects (e.g., interstitial nephritis).*  *2. **H2Bs may stabilize eGFR** via slower progression and higher regression rates.*  ***Recommendations**:*  *- Develop a risk score combining medication exposure, baseline eGFR, and transition rates to predict progression.*  *- Validate findings with a prospective cohort study tracking biomarkers (e.g., albuminuria).*  *---*  *## 6. Conclusion*  ***Summary**: PPI use is associated with faster CKD progression, particularly in G3. H2Bs may offer a safer alternative for at-risk patients.*  ***Next Steps**:*  *1. Host a workshop to refine clinical guidelines for PPI/H2B prescribing.*  *2. Pilot a monitoring protocol for PPI users in G3 (e.g., 3-month eGFR checks).*  *3. Explore process mining for real-time CKD trajectory prediction.*  ***Collaboration Invitation**: We propose a joint session to prioritize interventions and co-design a predictive model for CKD management.*  *```* |
