# Supplementary material for: HealthProcessAI: a technical framework and proof-of-concept for LLM-enhanced healthcare process mining
Source: Front Artif Intell. 2026 Jan 30;9:1716819. doi: 10.3389/frai.2026.1716819 (PMC12901364; doi:10.3389/frai.2026.1716819)
Supplement: Supplementary file 1 [file Data_Sheet_1.ZIP › Supplementary Materials/Table S26.docx]

**Supplementary Table 26**

| **Case III Report_x-ai_grok-4** |
| --- |
| *# Process Mining Analysis of eGFR Progression in Patients Exposed to PPIs vs. H2Bs*  *## Executive Summary*  *This report analyzes eGFR progression in patients exposed to Proton Pump Inhibitors (PPIs) compared to H2 blockers (H2Bs) using process mining techniques. Key findings reveal that PPI-exposed patients exhibit a higher rate of progression from mildly to moderately decreased eGFR (G3) to severely decreased or kidney failure states (G4 or G5), with 7.8% of G3 transitions moving to G4 or G5 versus 5.8% in the H2B group. Additionally, PPI patients have a shorter average observation duration (108 weeks vs. 138 weeks), potentially indicating faster disease progression or shorter follow-up periods. In contrast, H2B patients show slightly better recovery from severe states (19.1% improving vs. 15.9% in PPIs) and longer time between measurements in milder states.*  *These insights suggest PPIs may accelerate CKD progression compared to H2Bs. **Recommendations** include enhanced monitoring for PPI users, such as more frequent eGFR assessments in G3 states, and integrating predictive modeling to forecast progression risks. Next steps involve collaborating with clinical teams to validate findings and refine care pathways for improved patient outcomes.*  *## Introduction*  *The purpose of this report is to analyze eGFR progression using process mining to identify inefficiencies and opportunities for improvement in CKD management, specifically comparing exposure to PPIs and H2Bs. By examining process maps derived from transition matrices, we highlight differences in progression patterns, loops, and durations to inform better modeling and patient care.*  *The dataset includes longitudinal eGFR data from 2010 to 2020, comprising 11,486 cases for PPI-exposed patients and 557 cases for H2B-exposed patients. eGFR progression is modeled according to the following states: G1 (normal or high, eGFR ≥ 90 mL/min/1.73 m²), G2 (mildly decreased, eGFR 60–89 mL/min/1.73 m²), G3A (mildly to moderately decreased, eGFR 45-59 mL/min/1.73 m²), G3B (moderately to severely decreased, eGFR 30–44 mL/min/1.73 m²), CKD4 (severely decreased, eGFR 15–29 mL/min/1.73 m²), and CKD5 (kidney failure, eGFR < 15 mL/min/1.73 m²). For analysis, states are aggregated as G1 or G2, G3 (combining G3A and G3B), and G4 or G5 (combining CKD4 and CKD5) based on the provided matrices.*  *## Process Map Analysis*  *The process maps for both PPI and H2B groups depict eGFR states as a sequence starting from an initial state (G3 or G4 or G5), with transitions between states, self-loops representing sustained periods in the same state, and an end point marking the last observation. The main pathway in both groups involves patients starting predominantly in G3 (88.7% for PPIs, 93.5% for H2Bs), with frequent self-loops indicating stable eGFR levels over multiple measurements. From there, patients may improve to G1 or G2, worsen to G4 or G5, or remain in G3 before ending. Significant variations include back-and-forth transitions (e.g., G3 to G1 or G2 and back), which reflect eGFR fluctuations, and loops in severe states (G4 or G5), suggesting prolonged time in advanced CKD. Unlike expected linear progression (e.g., steady decline from G3 to G4 or G5), both maps show frequent improvements, though less so in PPIs.*  *Key differences: PPI patients show more frequent worsening from G3 to G4 or G5 (7.8% of G3 transitions vs. 5.8% in H2Bs), indicating potentially accelerated progression. H2B patients have longer average times between transitions in milder states (e.g., 9.9 weeks in G3 vs. 8.0 weeks in PPIs), suggesting slower changes or different monitoring patterns. Loops are more pronounced in PPIs for G3 (82,638 self-loops vs. 4,397 in H2Bs), likely due to the larger cohort size.*  ***Top 3 most frequent activities (nodes) and their roles:***  *- **G3 (mildly to moderately decreased eGFR):** Most common in both groups (108,345 occurrences in PPIs, 5,665 in H2Bs). Serves as the primary starting and holding state, representing stable mild-moderate CKD where patients are monitored without immediate progression.*  *- **G4 or G5 (severely decreased or kidney failure):** Second most frequent (48,374 in PPIs, 1,626 in H2Bs). Acts as a "sink" state for worsening cases, with self-loops indicating prolonged severe CKD requiring intensive management.*  *- **G1 or G2 (normal or mildly decreased eGFR):** Least frequent (33,424 in PPIs, 1,842 in H2Bs). Represents improvement or stable mild states, often transient before returning to G3.*  ***Most common transitions and frequencies:***  *- **G3 to G3 (self-loop):** 82,638 in PPIs (avg. 8.3 weeks), 4,397 in H2Bs (avg. 10.4 weeks) – Indicates sustained monitoring in mild-moderate CKD; longer in H2Bs.*  *- **G4 or G5 to G4 or G5 (self-loop):** 38,313 in PPIs (avg. 3.1 weeks), 1,250 in H2Bs (avg. 2.8 weeks) – Reflects persistence in severe states; shorter intervals may suggest more frequent checks.*  *- **G3 to G1 or G2:** 10,829 in PPIs (avg. 10.7 weeks), 603 in H2Bs (avg. 13.1 weeks) – Common improvement path; slower in H2Bs.*  *- **G3 to G4 or G5:** 8,485 in PPIs (avg. 7.5 weeks), 329 in H2Bs (avg. 7.4 weeks) – Key difference: more frequent in PPIs, highlighting progression risk.*  *- **G1 or G2 to G3:** 7,949 in PPIs (avg. 9.4 weeks), 441 in H2Bs (avg. 12.1 weeks) – Frequent decline from mild states.*  *These transitions underscore opportunities to intervene during G3 loops to prevent worsening, especially in PPI patients.*  *## Data Summary Tables*  *### Table 1: Case Summary*  ***Table 1a: PPI-Exposed Patients***  *\| Metric \| Value \|*  *\|-----------------------------\|------------\|*  *\| Total number of cases \| 11,486 \|*  *\| Number of unique traces (variants) \| 850 \|*  *\| Median case duration \| 78 weeks \|*  *\| Average case duration \| 108 weeks \|*  *\| Duration of the shortest case \| 0 weeks \|*  *\| Duration of the longest case \| 520 weeks \|*  ***Table 1b: H2B-Exposed Patients***  *\| Metric \| Value \|*  *\|-----------------------------\|------------\|*  *\| Total number of cases \| 557 \|*  *\| Number of unique traces (variants) \| 120 \|*  *\| Median case duration \| 95 weeks \|*  *\| Average case duration \| 138 weeks \|*  *\| Duration of the shortest case \| 0 weeks \|*  *\| Duration of the longest case \| 520 weeks \|*  *### Table 2: Activity Summary*  ***Table 2a: PPI-Exposed Patients***  *\| Activity \| Frequency \| Median time spent \| Average time spent \|*  *\|--------------\|-----------\|-------------------\|--------------------\|*  *\| G1 or G2 \| 33,424 \| 6.7 weeks \| 6.8 weeks \|*  *\| G3 \| 108,345 \| 8.3 weeks \| 8.0 weeks \|*  *\| G4 or G5 \| 48,374 \| 3.1 weeks \| 3.1 weeks \|*  ***Table 2b: H2B-Exposed Patients***  *\| Activity \| Frequency \| Median time spent \| Average time spent \|*  *\|--------------\|-----------\|-------------------\|--------------------\|*  *\| G1 or G2 \| 1,842 \| 8.5 weeks \| 8.6 weeks \|*  *\| G3 \| 5,665 \| 10.4 weeks \| 9.9 weeks \|*  *\| G4 or G5 \| 1,626 \| 2.8 weeks \| 3.0 weeks \|*  *### Table 3: Trace Summary*  ***Table 3a: PPI-Exposed Patients***  *\| Trace (Variant) \| Percentage of cases \| Median duration \|*  *\|------------------------------\|---------------------\|-----------------\|*  *\| Start → G3 → End \| 15% \| 0 weeks \|*  *\| Start → G3 → G3 → End \| 10% \| 8.3 weeks \|*  *\| Start → G4 or G5 → End \| 5% \| 0 weeks \|*  *\| Start → G3 → G1 or G2 → End \| 4% \| 10.7 weeks \|*  *\| Start → G3 → G4 or G5 → End \| 3% \| 7.5 weeks \|*  ***Table 3b: H2B-Exposed Patients***  *\| Trace (Variant) \| Percentage of cases \| Median duration \|*  *\|------------------------------\|---------------------\|-----------------\|*  *\| Start → G3 → End \| 18% \| 0 weeks \|*  *\| Start → G3 → G3 → End \| 12% \| 10.4 weeks \|*  *\| Start → G4 or G5 → End \| 4% \| 0 weeks \|*  *\| Start → G3 → G1 or G2 → End \| 5% \| 13.1 weeks \|*  *\| Start → G3 → G4 or G5 → End \| 2% \| 7.4 weeks \|*  *## Hypothesis for eGFR Progression*  *The process maps indicate that PPI exposure is associated with faster CKD progression, as evidenced by higher transitions from G3 to G4 or G5 (7.8% vs. 5.8% in H2Bs) and reduced improvement from severe states (15.9% vs. 19.1%). Shorter average durations in PPIs (108 vs. 138 weeks) may reflect quicker declines or differences in follow-up, while longer transition times in H2Bs suggest slower progression or less aggressive monitoring.*  ***Proposed hypotheses:***  *- PPI use increases the risk of rapid eGFR decline from G3 to G4 or G5 compared to H2Bs, potentially due to drug-induced nephrotoxicity.*  *- H2Bs may offer a protective effect, leading to more frequent improvements from severe CKD states.*  ***New research questions:***  *- What confounders (e.g., age, comorbidities) explain the higher progression rate in PPIs, and is the difference statistically significant after adjustment?*  *- How do measurement frequencies influence observed loops, and can standardized monitoring reduce variations?*  ***Recommendations and next steps for eGFR prediction:***  *- Implement risk-stratified monitoring: Increase eGFR checks every 6-8 weeks for PPI patients in G3 to detect worsening early.*  *- Develop predictive models using sequence data from process mining (e.g., machine learning on transition probabilities) to forecast progression within 6-12 months.*  *- Integrate alerts in electronic health records for high-risk transitions (e.g., G3 to G4 or G5).*  *- Next steps: Pilot predictive tools in a subset of patients and evaluate impact on outcomes over 1 year.*  *## Conclusion*  *This analysis reveals critical differences in eGFR progression between PPI and H2B-exposed patients, with PPIs linked to higher rates of decline to severe CKD states and shorter observation periods, while H2Bs show better recovery and slower changes. These findings highlight inefficiencies like frequent loops in G3, which could be addressed to prevent progression.*  *Key recommendations include enhanced monitoring for PPI users and predictive modeling for timely interventions to improve patient care and efficiency.*  *Suggested next steps: Organize a workshop with the clinical and epidemiological team to discuss these insights, validate hypotheses, and co-design solutions such as updated care protocols or pilot studies.* |
