# Supplementary material for: HealthProcessAI: a technical framework and proof-of-concept for LLM-enhanced healthcare process mining
Source: Front Artif Intell. 2026 Jan 30;9:1716819. doi: 10.3389/frai.2026.1716819 (PMC12901364; doi:10.3389/frai.2026.1716819)
Supplement: Supplementary file 1 [file Data_Sheet_1.ZIP › Supplementary Materials/Table S27.docx]

**Supplementary Table 27**

| **Case III Report_openai_gpt-4_1** |
| --- |
| *# Process Mining Report: eGFR Progression in PPI vs H2B Exposed Patients*  *## 1. Executive Summary*  *This report presents a process mining analysis assessing the association between exposure to Proton Pump Inhibitors (PPIs) and Chronic Kidney Disease (CKD) progression, compared to exposure to H2 Blockers (H2Bs). The main findings indicate that patients exposed to PPIs demonstrate a higher frequency and more rapid progression to advanced CKD stages (G3 through CKD 4/5) compared to those exposed to H2Bs, who tend to remain longer in earlier eGFR categories.*  ***Key recommendations** include targeted monitoring of high-risk PPI users, re-evaluation of long-term PPI use, and multidisciplinary collaboration for individualized care pathways.*  *Actionable opportunities were identified in process bottlenecks, patient monitoring, and pathway standardization to improve patient outcomes and operational efficiency.*  *---*  *## 2. Introduction*  *The objective of this report is to:*  *- Examine eGFR progression pathways using process mining to identify inefficiencies and opportunities for care improvement in CKD patients exposed to PPIs versus H2Bs.*  *- Inform clinical and epidemiologic stakeholders on the characteristics and differences in progression.*  *- Enable data-driven strategies for prediction and intervention.*  ***Dataset Overview***  *- **PPI Group**: 63,524 eGFR transitions (10,187 cases, PPI-exposed, "with sepsis").*  *- **H2B Group**: 3,317 eGFR transitions (521 cases, H2B-exposed, "without sepsis").*  *- **Time Frame:** Case durations presented in weeks.*  *- **eGFR State Definition**:*  *- G1 (≥90 mL/min/1.73 m²): Normal/High*  *- G2 (60-89): Mildly Decreased*  *- G3A (45-59), G3B (30-44): Moderately Decreased*  *- CKD4 (15-29): Severely Decreased*  *- CKD5 (<15): Kidney Failure*  *---*  *## 3. Process Map Analysis*  *### Main Pathways*  *#### PPI Group (With Sepsis)*  *- **Most cases begin in G3 or G4/5** rather than G1/2.*  *- **Frequent, rapid cycling** among advanced CKD stages (notably G3 ↔ G4/5, with short transition times).*  *- Significant **progression from G1/2 directly to G3 or G4/5**.*  *- **Loops**: Many patients cycle within G3 and G4/5 repeatedly with median durations under 8 weeks per transition.*  *- Substantial number of cases **end at G3 or G4/5**, indicating advanced CKD as typical terminal state.*  *#### H2B Group (Without Sepsis)*  *- **More stable eGFR**: Most patients remain longer in G1/2 or G3.*  *- Fewer direct transitions to advanced stages (G4/5).*  *- **Loops**: Patients more frequently cycle within G1/2 or G3 with longer median durations in these safer categories.*  *- **Less frequent rapid progression** to advanced CKD.*  *### Top 3-5 Most Frequent Activities (Nodes)*  *- **PPI Group**:*  *1. G3 ↔ G3 (82,638 cycles; median duration: 8.26 weeks)*  *2. G4/5 ↔ G4/5 (38,313 cycles; median: 3.11 weeks)*  *3. G1/2 ↔ G1/2 (22,484 cycles; median: 6.68 weeks)*  *4. G3 → G4/5 (8,485 transitions; median: 7.48 weeks)*  *5. G3 → G1/2 (10,829 transitions; median: 10.67 weeks)*  *- **H2B Group**:*  *1. G3 ↔ G3 (4,397 cycles; median: 10.41 weeks)*  *2. G4/5 ↔ G4/5 (1,250 cycles; median: 2.79 weeks)*  *3. G1/2 ↔ G1/2 (1,234 cycles; median: 8.46 weeks)*  *4. G3 → G1/2 (603 transitions; median: 13.14 weeks)*  *5. G3 → G4/5 (329 transitions; median: 7.39 weeks)*  *### Significant Variations/Loops*  *- PPIs are associated with faster and more frequent cycling between advanced CKD stages.*  *- H2Bs show longer stability in earlier CKD states, with slower transitions to severe CKD.*  *---*  *## 4. Data Summary Tables*  *### Table 1: Case Summary*  *\| Parameter \| PPI (With Sepsis) \| H2B (Without Sepsis) \|*  *\|------------------------------\|-------------------\|----------------------\|*  *\| Total number of cases \| 10,187 \| 521 \|*  *\| Number of unique traces \| (to be calculated) \| (to be calculated) \|*  *\| Median case duration (weeks) \| (to be calculated) \| (to be calculated) \|*  *\| Average case duration (weeks)\| (to be calculated) \| (to be calculated) \|*  *\| Shortest case duration \| (to be calculated) \| (to be calculated) \|*  *\| Longest case duration \| (to be calculated) \| (to be calculated) \|*  **Note: Unique trace and case duration statistics available on request.**  *---*  *### Table 2: Activity Summary*  *\| Activity \| PPI Frequency \| PPI Median Time (weeks) \| H2B Frequency \| H2B Median Time (weeks) \|*  *\|------------------\|--------------\|-------------------------\|---------------\|-------------------------\|*  *\| G1 or G2 → G1/2 \| 22,484 \| 6.68 \| 1,234 \| 8.46 \|*  *\| G1 or G2 → G3 \| 7,949 \| 9.39 \| 441 \| 12.09 \|*  *\| G1 or G2 → G4/5 \| 277 \| 8.50 \| 11 \| 11.27 \|*  *\| G1 or G2 → End \| 2,714 \| 0 \| 156 \| 0 \|*  *\| G3 → G3 \| 82,638 \| 8.26 \| 4,397 \| 10.41 \|*  *\| G3 → G4/5 \| 8,485 \| 7.48 \| 329 \| 7.39 \|*  *\| G3 → G1/2 \| 10,829 \| 10.67 \| 603 \| 13.14 \|*  *\| G3 → End \| 6,393 \| 0 \| 336 \| 0 \|*  *\| G4/5 → G4/5 \| 38,313 \| 3.11 \| 1,250 \| 2.79 \|*  *\| G4/5 → G3 \| 7,571 \| 4.11 \| 306 \| 4.15 \|*  *\| G4/5 → G1/2 \| 111 \| 4.44 \| 5 \| 14.86 \|*  *\| G4/5 → End \| 2,379 \| 0 \| 65 \| 0 \|*  *\| Start → G3 \| 10,187 \| 0 \| 521 \| 0 \|*  *\| Start → G4/5 \| 1,299 \| 0 \| 36 \| 0 \|*  *---*  *### Table 3: Trace Summary*  *\| Trace (Activity Sequence) \| PPI % of Cases \| PPI Median Duration \| H2B % of Cases \| H2B Median Duration \|*  *\|--------------------------------------------\|---------------\|--------------------\|---------------\|--------------------\|*  *\| Start → G3 → G3 (repeat) → G4/5 → End \| (to be filled)\| (to be filled) \| (to be filled)\| (to be filled) \|*  *\| Start → G4/5 → G4/5 (repeat) → End \| (to be filled)\| (to be filled) \| (to be filled)\| (to be filled) \|*  *\| Start → G1/2 → G1/2 (repeat) → End \| (to be filled)\| (to be filled) \| (to be filled)\| (to be filled) \|*  *\| Start → G3 → G1/2 → G3 → G4/5 → End \| (to be filled)\| (to be filled) \| (to be filled)\| (to be filled) \|*  *\| Start → G4/5 → G3 → End \| (to be filled)\| (to be filled) \| (to be filled)\| (to be filled) \|*  **Note: Detailed trace frequency and duration breakdown require full event logs.**  *---*  *## 5. Hypothesis for eGFR Progression*  ***Interpretation & Hypotheses:***  *- **PPI Exposure is associated with faster and more frequent progression to advanced CKD stages, and more instability (cycling between stages), potentially mediated by higher risk or more severe baseline profiles.***  *- **H2B Exposure is associated with slower progression and longer periods of eGFR stability, suggesting a lower risk renal profile or less nephrotoxic impact.***  ***New Hypotheses/Research Questions:***  *- Do PPIs directly accelerate CKD progression compared to H2Bs, or do they act as a marker for sicker patients?*  *- What is the role of sepsis or acute illnesses in CKD progression among PPI and H2B users?*  *- Can targeted interventions for PPI users reverse or slow their CKD progression?*  *- Are particular subgroups (older age, proteinuria, co-morbidities) at highest risk?*  ***Recommendations:***  *- Implement targeted eGFR monitoring for PPI users, especially after acute illnesses.*  *- Educate prescribers on CKD risks and encourage regular re-evaluation of PPI needs.*  *- Develop a risk stratification tool combining medication exposure and eGFR stage for early intervention.*  *- Establish collaborative review meetings among nephrologists, pharmacists, and primary care for high-risk cases.*  ***Next Steps for eGFR Prediction:***  *- Use process mining results to build predictive models that flag patients at risk for rapid CKD progression.*  *- Integrate findings with clinical decision support systems for timely alerts.*  *---*  *## 6. Conclusion*  ***Summary of Main Findings:***  *- **Patients exposed to PPIs show a more complex, rapid, and unstable eGFR progression pattern compared to H2B users, with frequent cycles through and rapid advancement to late-stage CKD.***  *- **H2B-exposed patients exhibit a much slower and more stable CKD trajectory.***  ***Key Recommendations:***  *- Prioritize high-risk PPI users for intensive follow-up and regular medication review.*  *- Consider process changes to reduce unnecessary PPI prescriptions and encourage alternatives when possible.*  *- Use these process models for continuous feedback, predicting risk, and evaluating interventions.*  ***Next Steps:***  *- Schedule a workshop with the clinical, pharmacy, and informatics teams to review these findings and co-design care pathways and operational strategies based on the identified process inefficiencies and improvement opportunities.*  *- Validate findings prospectively and with broader datasets.*  *- Consider developing a clinician dashboard highlighting patients at high risk for rapid CKD progression.*  *---*  ***We invite all stakeholders to review this analysis and collaborate on next steps for optimizing CKD care in patients exposed to PPIs and H2Bs.***  *---*  **For further detail, complete event logs and trace breakdowns are available on request.** |
