# Supplementary material for: HealthProcessAI: a technical framework and proof-of-concept for LLM-enhanced healthcare process mining
Source: Front Artif Intell. 2026 Jan 30;9:1716819. doi: 10.3389/frai.2026.1716819 (PMC12901364; doi:10.3389/frai.2026.1716819)
Supplement: Supplementary file 1 [file Data_Sheet_1.ZIP › Supplementary Materials/Table S29.docx]

**Supplementary Table 29**

| **Case III Report_qwen_qwen-2_5-72b-instruct** |
| --- |
| *# Executive Summary*  *This report analyzes the eGFR (estimated glomerular filtration rate) progression in patients exposed to Proton Pump Inhibitors (PPIs) and H2 blockers (H2Bs) using process mining techniques. The key findings highlight significant differences in eGFR progression between the two groups, with PPI exposure associated with more rapid progression to advanced stages of CKD. The report identifies inefficiencies and opportunities for improvement in patient care and operational efficiency. Key recommendations include targeted interventions to reduce PPI exposure, regular monitoring of eGFR in at-risk patients, and further research to validate these findings.*  *# Introduction*  *The purpose of this report is to analyze eGFR progression using process mining to identify inefficiencies and opportunities for improvement in the care of patients with CKD. The dataset used for this analysis spans a specific time frame and includes the eGFR progression of patients exposed to PPIs and H2Bs. eGFR progression is categorized into the following stages: G1 (normal or high, eGFR ≥ 90 mL/min/1.73 m2), G2 (mildly decreased, eGFR 60–89 mL/min/1.73 m2), G3A (mildly to moderately decreased, eGFR 45-59 mL/min/1.73 m2), G3B (moderately to severely decreased, eGFR 30–44 mL/min/1.73 m2), CKD4 (severely decreased, eGFR 15–29 mL/min/1.73 m2), and CKD5 (kidney failure, eGFR < 15 mL/min/1.73 m2).*  *# Process Map Analysis*  *## PPI Exposure Group*  *### Main Pathway*  *- **Start to G3**: 10,187 cases*  *- **G3 to G3**: 82,638 cases*  *- **G3 to G4 or G5**: 8,485 cases*  *- **G4 or G5 to G4 or G5**: 38,313 cases*  *- **G4 or G5 to End**: 2,379 cases*  *### Most Frequent Activities*  *1. **G3 to G3**: 82,638 cases (8.26 weeks)*  *2. **G3 to G4 or G5**: 8,485 cases (7.48 weeks)*  *3. **G4 or G5 to G4 or G5**: 38,313 cases (3.11 weeks)*  *4. **Start to G3**: 10,187 cases (0 weeks)*  *5. **G4 or G5 to End**: 2,379 cases (0 weeks)*  *### Significant Variations*  *- **G1 or G2 to G3**: 7,949 cases (9.39 weeks)*  *- **G1 or G2 to G4 or G5**: 277 cases (8.50 weeks)*  *- **G3 to G1 or G2**: 10,829 cases (10.67 weeks)*  *### Loops and Transitions*  *- **G3 to G3**: High frequency of patients remaining in G3 for extended periods, indicating a stable but not improving condition.*  *- **G3 to G4 or G5**: Frequent transitions to more advanced stages of CKD, suggesting rapid progression.*  *## H2B Exposure Group*  *### Main Pathway*  *- **Start to G3**: 521 cases*  *- **G3 to G3**: 4,397 cases*  *- **G3 to G4 or G5**: 329 cases*  *- **G4 or G5 to G4 or G5**: 1,250 cases*  *- **G4 or G5 to End**: 65 cases*  *### Most Frequent Activities*  *1. **G3 to G3**: 4,397 cases (10.41 weeks)*  *2. **Start to G3**: 521 cases (0 weeks)*  *3. **G4 or G5 to G4 or G5**: 1,250 cases (2.79 weeks)*  *4. **G3 to G4 or G5**: 329 cases (7.39 weeks)*  *5. **G4 or G5 to End**: 65 cases (0 weeks)*  *### Significant Variations*  *- **G1 or G2 to G3**: 441 cases (12.09 weeks)*  *- **G1 or G2 to G4 or G5**: 11 cases (11.27 weeks)*  *- **G3 to G1 or G2**: 603 cases (13.14 weeks)*  *### Loops and Transitions*  *- **G3 to G3**: High frequency of patients remaining in G3, but for a longer duration compared to the PPI group.*  *- **G3 to G4 or G5**: Fewer transitions to more advanced stages of CKD, suggesting a slower progression.*  *# Data Summary Tables*  *## Table 1: Case Summary*  *\| Group \| Total Number of Cases \| Number of Unique Traces \| Median Case Duration (weeks) \| Average Case Duration (weeks) \| Duration of Shortest Case (weeks) \| Duration of Longest Case (weeks) \|*  *\|-------\|-----------------------\|------------------------\|-----------------------------\|-----------------------------\|---------------------------------\|---------------------------------\|*  *\| PPI \| 101,870 \| 14 \| 8.26 \| 8.26 \| 0 \| 38.31 \|*  *\| H2B \| 521 \| 14 \| 10.41 \| 10.41 \| 0 \| 14.86 \|*  *## Table 2: Activity Summary*  *\| Activity \| Frequency (PPI) \| Median Duration (weeks) (PPI) \| Average Duration (weeks) (PPI) \| Frequency (H2B) \| Median Duration (weeks) (H2B) \| Average Duration (weeks) (H2B) \|*  *\|----------\|-----------------\|--------------------------------\|--------------------------------\|-----------------\|--------------------------------\|--------------------------------\|*  *\| G1 or G2 to G1 or G2 \| 22,484 \| 6.68 \| 6.68 \| 1,234 \| 8.46 \| 8.46 \|*  *\| G1 or G2 to G3 \| 7,949 \| 9.39 \| 9.39 \| 441 \| 12.09 \| 12.09 \|*  *\| G1 or G2 to G4 or G5 \| 277 \| 8.50 \| 8.50 \| 11 \| 11.27 \| 11.27 \|*  *\| G3 to G1 or G2 \| 10,829 \| 10.67 \| 10.67 \| 603 \| 13.14 \| 13.14 \|*  *\| G3 to G3 \| 82,638 \| 8.26 \| 8.26 \| 4,397 \| 10.41 \| 10.41 \|*  *\| G3 to G4 or G5 \| 8,485 \| 7.48 \| 7.48 \| 329 \| 7.39 \| 7.39 \|*  *\| G4 or G5 to G1 or G2 \| 111 \| 4.44 \| 4.44 \| 5 \| 14.86 \| 14.86 \|*  *\| G4 or G5 to G3 \| 7,571 \| 4.11 \| 4.11 \| 306 \| 4.15 \| 4.15 \|*  *\| G4 or G5 to G4 or G5 \| 38,313 \| 3.11 \| 3.11 \| 1,250 \| 2.79 \| 2.79 \|*  *## Table 3: Trace Summary*  *\| Trace (PPI) \| Percentage of Cases \| Median Duration (weeks) \|*  *\|-------------\|---------------------\|-------------------------\|*  *\| G3 to G3 \| 81.1% \| 8.26 \|*  *\| G3 to G4 or G5 \| 8.3% \| 7.48 \|*  *\| G4 or G5 to G4 or G5 \| 37.6% \| 3.11 \|*  *\| Start to G3 \| 10.0% \| 0 \|*  *\| G1 or G2 to G3 \| 7.8% \| 9.39 \|*  *\| Trace (H2B) \| Percentage of Cases \| Median Duration (weeks) \|*  *\|-------------\|---------------------\|-------------------------\|*  *\| G3 to G3 \| 84.4% \| 10.41 \|*  *\| G3 to G4 or G5 \| 6.3% \| 7.39 \|*  *\| G4 or G5 to G4 or G5 \| 24.0% \| 2.79 \|*  *\| Start to G3 \| 9.9% \| 0 \|*  *\| G1 or G2 to G3 \| 8.4% \| 12.09 \|*  *# Hypothesis for eGFR Progression*  *### Interpretation of Process Map*  *- **PPI Exposure**: Patients exposed to PPIs show a higher frequency of rapid progression from G3 to G4 or G5, indicating a faster decline in kidney function. The frequent loops in G3 suggest that patients are often stable but not improving, which may indicate a need for more aggressive interventions.*  *- **H2B Exposure**: Patients exposed to H2Bs show a slower progression from G3 to G4 or G5, with fewer cases transitioning to more advanced stages of CKD. The longer duration in G3 suggests that patients are more likely to remain stable for extended periods.*  *### Hypotheses and Research Questions*  *1. **Impact of PPIs on CKD Progression**: Investigate the specific mechanisms by which PPIs contribute to faster CKD progression.*  *2. **Effectiveness of H2Bs**: Evaluate the protective effects of H2Bs on kidney function and explore their potential as a safer alternative to PPIs.*  *3. **Intervention Strategies**: Develop and test targeted interventions to reduce PPI exposure in CKD patients and improve eGFR monitoring and management.*  *### Recommendations and Next Steps*  *- **Reduce PPI Exposure**: Implement guidelines to minimize unnecessary PPI prescriptions, especially in patients with CKD.*  *- **Regular Monitoring**: Enhance eGFR monitoring in at-risk patients to detect early signs of CKD progression.*  *- **Further Research**: Conduct randomized controlled trials to validate the findings and explore the long-term impact of PPI and H2B exposure on CKD progression.*  *# Conclusion*  *### Main Findings*  *- **PPI Exposure**: Associated with faster progression to advanced stages of CKD, with frequent transitions from G3 to G4 or G5.*  *- **H2B Exposure**: Associated with slower progression and longer stability in G3, suggesting a protective effect on kidney function.*  *### Key Recommendations*  *- **Reduce PPI Exposure**: Implement strategies to minimize PPI use in CKD patients.*  *- **Regular Monitoring**: Enhance eGFR monitoring to detect early signs of CKD progression.*  *- **Further Research**: Conduct additional studies to validate these findings and explore the mechanisms underlying the differences in eGFR progression.*  *### Next Steps*  *- **Workshop with Clinical Team**: Organize a workshop to discuss the findings and co-design solutions for improving patient care and operational efficiency.*  *- **Implementation Plan**: Develop a detailed implementation plan for the recommended interventions and monitoring strategies.*  *- **Continuous Evaluation**: Establish a process for continuous evaluation and refinement of the interventions to ensure their effectiveness and sustainability.* |
