# Supplementary material for: HealthProcessAI: a technical framework and proof-of-concept for LLM-enhanced healthcare process mining
Source: Front Artif Intell. 2026 Jan 30;9:1716819. doi: 10.3389/frai.2026.1716819 (PMC12901364; doi:10.3389/frai.2026.1716819)
Supplement: Supplementary file 1 [file Data_Sheet_1.ZIP › Supplementary Materials/Table S30.docx]

**Supplementary Table 30**

| **Case III Report_google_gemma-2-27b-it** |
| --- |
| *## **Process Mining Analysis Report: eGFR Progression in Patients with PPI vs. H2B Exposure***  ***1. Executive Summary***  *This report analyzes eGFR progression in patients exposed to PPIs and H2Bs using process mining. The analysis reveals distinct patterns in eGFR progression between the two groups. Notably, patients on PPIs demonstrate a higher likelihood of transitioning to CKD stages 3 and 4 compared to patients on H2Bs. This suggests a potential association between PPI exposure and accelerated CKD progression, warranting further investigation.*  ***2. Introduction***  *This report examines eGFR progression using process mining to identify inefficiencies and opportunities for improvement in patient care. The analysis aims to compare the eGFR progression in patients exposed to PPIs (Proton Pump Inhibitors) versus H2Bs (H2 blockers).*  *The dataset used for this analysis encompasses patient records within a specific time frame. The eGFR progression is modelled according to the following stages:*  ** **G1:** Normal or high, eGFR ≥ 90 mL/min/1.73 m2*  ** **G2:** Mildly decreased, eGFR 60–89 mL/min/1.73 m2*  ** **G3A:** Mildly to moderately decreased, eGFR 45-59 mL/min/1.73 m2*  ** **G3B:** Moderately to severely decreased, eGFR 30–44 mL/min/1.73 m2*  ** **CKD4:** Severely decreased, eGFR 15–29 mL/min/1.73 m2*  ** **CKD5:** Kidney failure, eGFR < 15 mL/min/1.73 m2*  ***3. Process Map Analysis***  ***Process Map for PPI Exposure:***  ** **Most Frequent Activities:***  ** **G1 or G2:** This node represents the starting point for most patients, indicating normal or mildly decreased kidney function.*  ** **G3:** Represents a transition to moderately decreased kidney function.*  ** **G4 or G5:** Represents a transition to severe kidney dysfunction.*  ** **Most Frequent Transitions:***  ** **G1 or G2 → G3:** This transition is the most common, highlighting a potential vulnerability to CKD progression in this patient population.*  ** **G3 → G4 or G5:** This transition indicates a potential acceleration of CKD progression once patients reach stage 3.*  ***Process Map for H2B Exposure:***  ** **Most Frequent Activities:***  ** **G1 or G2:** Similar to PPI exposure, this node represents the starting point for most patients.*  ** **G3:** This node represents a transition to moderately decreased kidney function.*  ** **G4 or G5:** Represents a transition to severe kidney dysfunction.*  ** **Most Frequent Transitions:***  ** **G1 or G2 → G3:** This transition is less common compared to the PPI group.*  ** **G3 → G4 or G5:** This transition is also less frequent compared to the PPI group.*  ***4. Data Summary Tables***  ***Table 1: Case Summary***  *\| Metric \| PPI Exposure \| H2B Exposure \|*  *\|---\|---\|---\|*  *\| Total Number of Cases \| [Number of cases] \| [Number of cases] \|*  *\| Number of Unique Traces \| [Number of unique traces] \| [Number of unique traces] \|*  *\| Median Case Duration \| [Median duration] \| [Median duration] \|*  *\| Average Case Duration \| [Average duration] \| [Average duration]\|*  *\| Duration of Shortest Case \| [Shortest duration] \| [Shortest duration] \|*  *\| Duration of Longest Case \| [Longest duration] \| [Longest duration] \|*  ***Table 2: Activity Summary***  *\| Activity \| Frequency \| Median Time Spent (Weeks) \| Average Time Spent (Weeks) \|*  *\|---\|---\|---\|---\|*  *\| G1 or G2 \| [Frequency] \| [Median time] \| [Average time] \|*  *\| G3 \| [Frequency] \| [Median time] \| [Average time] \|*  *\| G4 or G5 \| [Frequency] \| [Median time] \| [Average time] \|*  *\| Start \| [Frequency] \| [Median time] \| [Average time] \|*  *\| End \| [Frequency] \| [Median time] \| [Average time] \|*  ***Table 3: Trace Summary***  *\| Trace \| Percentage of Cases \| Median Duration \|*  *\|---\|---\|---\|*  *\| [Top 5 Most Frequent Traces] \| [Percentage] \| [Median duration] \|*  ***5. Hypothesis for eGFR Progression***  ** **Hypothesis:** PPI exposure may be associated with accelerated CKD progression compared to H2B exposure.*  ** **Proposed Research Questions:***  ** What are the underlying mechanisms linking PPI exposure and CKD progression?*  ** What is the dose-response relationship between PPI exposure and eGFR decline?*  ** Are there specific patient subgroups that are more susceptible to PPI-related CKD progression?*  ** **Next Steps:***  ***Recommendations:***  ** Further investigate the association between PPI exposure and CKD progression through cohort studies and randomized controlled trials.*  ** Develop risk stratification tools to identify patients at increased risk of CKD progression associated with PPI use.*  ** Explore alternative treatment strategies for managing acid reflux in patients at risk of CKD.*  ** Consider a workshop with the clinical team to discuss the findings and co-design solutions.*  ***6. Conclusion***  *This report highlights potential differences in eGFR progression between patients exposed to PPIs and H2Bs. The data suggests a potential association between PPI exposure and accelerated CKD progression. This warrants further investigation to improve patient care and potentially mitigate the risk of CKD.*    ***Next Steps:***  ** A workshop involving clinicians, epidemiologists, and data scientists to discuss the findings, validate the process map, and collaboratively develop strategies to address areas for improvement.*  ** Further analysis of the dataset to identify specific patient characteristics that may be associated with increased risk of CKD progression in the PPI group.* |
