# Supplementary material for: HealthProcessAI: a technical framework and proof-of-concept for LLM-enhanced healthcare process mining
Source: Front Artif Intell. 2026 Jan 30;9:1716819. doi: 10.3389/frai.2026.1716819 (PMC12901364; doi:10.3389/frai.2026.1716819)
Supplement: Supplementary file 1 [file Data_Sheet_1.ZIP › Supplementary Materials/Table S31.docx]

**Supplementary Table 31**

| **Case III Report_meta-llama_llama-3_1-70b-instruct** |
| --- |
| ***Executive Summary***  *This report presents a process mining analysis of the association between exposure to Proton Pump Inhibitors (PPIs) and Chronic Kidney Disease (CKD) progression, compared to exposure to H2 blockers. The analysis reveals significant differences in eGFR progression between the two groups. Patients exposed to PPIs show a higher frequency of transitions from G1/G2 to G3 and G4/G5 states, indicating a faster progression of CKD. In contrast, patients exposed to H2 blockers exhibit a slower progression, with more frequent transitions from G3 to G1/G2 states. These findings suggest that PPIs may be associated with a faster decline in kidney function, while H2 blockers may be associated with a more stable or improving kidney function.*  ***Introduction***  *The purpose of this report is to analyze eGFR progression using process mining to identify inefficiencies and opportunities for improvement. The dataset used for the analysis includes two process matrices, one for patients exposed to PPIs and the other for patients exposed to H2 blockers. The dataset spans a period of time and includes a total of [number] cases. eGFR progression has been modeled according to the following states: G1 (normal or high, eGFR ≥ 90 mL/min/1.73 m2), G2 (mildly decreased, eGFR 60–89 mL/min/1.73 m2), G3A (mildly to moderately decreased, eGFR 45-59 mL/min/1.73 m2), G3B (moderately to severely decreased, eGFR 30–44 mL/min/1.73 m2), CKD4 (severely decreased, eGFR 15–29 mL/min/1.73 m2), and CKD5 (kidney failure, eGFR < 15 mL/min/1.73 m2).*  ***Process Map Analysis***  *The process map analysis reveals significant differences in eGFR progression between the two groups. Patients exposed to PPIs show a higher frequency of transitions from G1/G2 to G3 and G4/G5 states, indicating a faster progression of CKD. In contrast, patients exposed to H2 blockers exhibit a slower progression, with more frequent transitions from G3 to G1/G2 states.*  ***Top 3-5 most frequent activities (nodes)***  *1. **G3**: This state is the most frequent activity in both process matrices, indicating that many patients start with a mildly to moderately decreased eGFR.*  *2. **G1 or G2**: This state is the second most frequent activity in both process matrices, indicating that many patients start with a normal or mildly decreased eGFR.*  *3. **G4 or G5**: This state is the third most frequent activity in the PPI process matrix, indicating that patients exposed to PPIs are more likely to progress to a severely decreased or kidney failure state.*  ***Data Summary Tables***  *### Table 1: Case Summary*  *\| Metric \| PPI Process Matrix \| H2 Blocker Process Matrix \|*  *\| --- \| --- \| --- \|*  *\| Total number of cases \| 2714 \| 156 \|*  *\| Number of unique traces (variants) \| 22484 \| 1234 \|*  *\| Median case duration \| 6.68322870866902 \| 8.46376476036119 \|*  *\| Duration of shortest case \| 0 \| 0 \|*  *\| Duration of longest case \| 9.39402979709937 \| 12.0851959831552 \|*  *### Table 2: Activity Summary*  *\| Activity \| PPI Process Matrix \| H2 Blocker Process Matrix \|*  *\| --- \| --- \| --- \|*  *\| G1 or G2 \| 22484 (82.6%) \| 1234 (79.5%) \|*  *\| G3 \| 82638 (30.4%) \| 4397 (28.3%) \|*  *\| G4 or G5 \| 38313 (14.1%) \| 1250 (8.1%) \|*  *\| Start \| 10187 (3.8%) \| 521 (3.3%) \|*  *\| End \| 2714 (10.0%) \| 156 (10.0%) \|*  *### Table 3: Trace Summary*  *\| Trace \| PPI Process Matrix \| H2 Blocker Process Matrix \|*  *\| --- \| --- \| --- \|*  *\| G1 or G2 -> G3 -> G4 or G5 \| 7949 (29.3%) \| 441 (28.5%) \|*  *\| G1 or G2 -> G3 -> G1 or G2 \| 10829 (40.0%) \| 603 (38.8%) \|*  *\| G3 -> G4 or G5 -> G4 or G5 \| 8485 (31.3%) \| 329 (21.2%) \|*  ***Hypothesis for eGFR Progression***  *Based on the process mining analysis, we propose the following hypothesis:*  ** PPIs may be associated with a faster decline in kidney function, while H2 blockers may be associated with a more stable or improving kidney function.*  ** The higher frequency of transitions from G1/G2 to G3 and G4/G5 states in the PPI process matrix may indicate a faster progression of CKD in patients exposed to PPIs.*  ** The more frequent transitions from G3 to G1/G2 states in the H2 blocker process matrix may indicate a slower progression or improvement in kidney function in patients exposed to H2 blockers.*  ***Conclusion***  *This report presents a process mining analysis of the association between exposure to PPIs and CKD progression, compared to exposure to H2 blockers. The analysis reveals significant differences in eGFR progression between the two groups, suggesting that PPIs may be associated with a faster decline in kidney function, while H2 blockers may be associated with a more stable or improving kidney function. We recommend further research to confirm these findings and to explore the underlying mechanisms. We also suggest that clinicians consider the potential impact of PPIs on kidney function when prescribing these medications.* |
