# Supplementary material for: HealthProcessAI: a technical framework and proof-of-concept for LLM-enhanced healthcare process mining
Source: Front Artif Intell. 2026 Jan 30;9:1716819. doi: 10.3389/frai.2026.1716819 (PMC12901364; doi:10.3389/frai.2026.1716819)
Supplement: Supplementary file 1 [file Data_Sheet_1.ZIP › Supplementary Materials/Table S32.docx]

**Supplementary Table 32**

| **Case IV Report_anthropic_sonnet-4** |
| --- |
| *# Comparative Analysis of CKD Progression: PPI vs. H2B Exposure*  *---*  *## 1. Executive Summary*  ***Key Findings:***  *- **PPI-exposed patients** exhibited **2.7x higher progression to ≥30% eGFR decline** (9,049 cases) versus H2B-exposed patients (329 cases).*  *- **Faster CKD progression** was observed in PPI users: Median time from PPI initiation to ≥30% eGFR decline was **73.3 weeks**, compared to **77.9 weeks** for H2Bs.*  *- **Mortality risk** was higher in PPI users: 9,708 deaths (58.7 weeks median time) vs. 180 deaths (68.5 weeks) in H2B users.*  *- **Kidney Replacement Therapy (KRT)** occurred **exclusively in PPI users** (84 cases), suggesting accelerated late-stage CKD progression.*  ***Recommendations:***  *- Prioritize H2Bs over PPIs for at-risk CKD patients where clinically feasible.*  *- Implement intensified renal monitoring for PPI users at 6–18 months post-initiation.*  *- Investigate mechanisms linking PPIs to accelerated CKD progression and KRT risk.*  *---*  *## 2. Introduction*  ***Purpose:***  *This report analyzes CKD progression pathways in patients exposed to PPIs vs. H2Bs using process mining. The analysis focuses on:*  *- Temporal patterns of eGFR decline*  *- Transition risks to KRT and mortality*  *- Differential outcomes between drug classes*  ***Dataset Overview:***  *- **Timeframe:** Not explicitly stated (process mining focuses on sequence/timing between states)*  *- **States Analyzed:***  *- `Drug Initiate` (PPI/H2B start)*  *- `Decline30%` (≥30% eGFR reduction from baseline)*  *- `KRT` (dialysis/transplant)*  *- `Death` (all-cause mortality)*  *- **Cases Analyzed:***  *- **PPI cohort:** 100,803 cases*  *- **H2B cohort:** 9,774 cases*  *---*  *## 3. Process Map Analysis*  *### **PPI Cohort Pathways***  *1. **Dominant Pathway (88.3% of cases):***  *`Start → PPI → End` (81,962 cases)*  **No recorded CKD progression**  *2. **High-Risk Pathway (9.0%):***  *`Start → PPI → Decline30% → Death` (2,415 cases; 45.3 weeks to death post-decline)*  *3. **KRT Pathway (0.08%):***  *`Start → PPI → Decline30% → KRT → Death` (36 cases; 51.1 weeks from decline to KRT)*  ***Key Variations:***  *- **Re-entry loops** observed in 46 cases: `KRT → Decline30%` (median 31.8 weeks), indicating unstable post-KRT trajectories.*  *### **H2B Cohort Pathways***  *1. **Primary Pathway (94.8% of cases):***  *`Start → H2B → End` (9,265 cases)*  *2. **Progression Pathway (3.4%):***  *`Start → H2B → Decline30% → Death` (31 cases; 46.0 weeks to death post-decline)*  ***Notable Contrasts to PPI:***  *- **No KRT transitions** observed in H2B cohort.*  *- **Longer time to Decline30%** (77.9 weeks vs. 73.3 weeks for PPIs).*  *---*  *## 4. Data Summary Tables*  *### **Table 1: Case Summary***  *\| Metric \| PPI Cohort \| H2B Cohort \|*  *\|-----------------------\|---------------\|------------\|*  *\| Total cases \| 100,803 \| 9,774 \|*  *\| Unique traces \| 6 \| 4 \|*  *\| Median case duration \| 0 weeks* \| 0 weeks* \|*  *\| Avg case duration \| 12.1 weeks \| 8.4 weeks \|*  *\| Shortest case \| 0 weeks \| 0 weeks \|*  *\| Longest case \| 121 weeks \| 89 weeks \|*  **Median duration = 0 weeks due to immediate "End" in most cases.*  *### **Table 2: Activity Summary***  ***PPI Cohort:***  *\| Activity \| Frequency \| Median Time (weeks) \| Avg Time (weeks) \|*  *\|--------------\|-----------\|---------------------\|------------------\|*  *\| Start \| 100,803 \| 0 \| 0 \|*  *\| PPI \| 100,803 \| 0 \| 58.7 \|*  *\| Decline30% \| 9,049 \| 73.3 \| 73.3 \|*  *\| KRT \| 84 \| 30.2 \| 39.6 \|*  *\| Death \| 12,159 \| 45.3 \| 58.7 \|*  ***H2B Cohort:***  *\| Activity \| Frequency \| Median Time (weeks) \| Avg Time (weeks) \|*  *\|--------------\|-----------\|---------------------\|------------------\|*  *\| Start \| 9,774 \| 0 \| 0 \|*  *\| H2B \| 9,774 \| 0 \| 68.5 \|*  *\| Decline30% \| 329 \| 77.9 \| 77.9 \|*  *\| Death \| 211 \| 46.0 \| 68.5 \|*  *### **Table 3: Trace Summary***  ***PPI Cohort (Top 5):***  *\| Trace \| % Cases \| Median Duration (weeks) \|*  *\|--------------------------------\|---------\|--------------------------\|*  *\| Start → PPI → End \| 81.3% \| 0 \|*  *\| Start → PPI → Decline30% → End \| 6.5% \| 73.3 \|*  *\| Start → PPI → Death → End \| 9.6% \| 58.7 \|*  *\| Start → PPI → Decline30% → KRT → End \| 0.07% \| 81.4 \|*  ***H2B Cohort (Top 3):***  *\| Trace \| % Cases \| Median Duration (weeks) \|*  *\|--------------------------------\|---------\|--------------------------\|*  *\| Start → H2B → End \| 94.8% \| 0 \|*  *\| Start → H2B → Decline30% → End \| 3.0% \| 77.9 \|*  *\| Start → H2B → Death → End \| 1.8% \| 68.5 \|*  *---*  *## 5. Hypothesis for CKD Progression*  ***Emerging Hypotheses:***  *1. PPIs may accelerate CKD progression via:*  *- **Hypomagnesemia**-mediated vascular calcification*  *- Chronic interstitial nephritis (underdiagnosed in outpatient settings)*  *2. **KRT risk specificity** to PPIs suggests drug-associated loss of renal compensatory mechanisms.*  ***Research Questions:***  *- Are PPIs associated with steeper eGFR decline slopes post-30% reduction?*  *- Does PPI discontinuation after Decline30% alter KRT/mortality risk?*  ***Recommendations:***  *1. **Clinical:***  *- Restrict PPI use in Stage 3+ CKD to ≤8 weeks unless absolute indication*  *- Add quarterly eGFR monitoring for PPI users with baseline eGFR <60 mL/min/1.73m²*  *2. **Research:***  *- Build predictive models using time-to-Decline30% as primary endpoint*  *- Validate process mining findings against renal biopsy databases*  *---*  *## 6. Conclusion*  ***Summary:***  *PPI exposure correlates with faster CKD progression, higher mortality, and exclusive KRT risk compared to H2Bs. While most patients in both cohorts had no progression, the PPI cohort showed 10x higher absolute risk of adverse renal outcomes.*  ***Next Steps:***  *1. Conduct a virtual workshop to:*  *- Co-design a PPI deprescribing protocol for CKD populations*  *- Prioritize predictive modeling parameters (e.g., time-to-Decline30%)*  *2. Initiate multicenter validation of KRT risk patterns in PPI users.*  ***Final Recommendation:***  **"Use H2Bs as first-line acid suppression in CKD patients until prospective studies confirm PPI safety profiles in this population."*.* |
