# Supplementary material for: HealthProcessAI: a technical framework and proof-of-concept for LLM-enhanced healthcare process mining
Source: Front Artif Intell. 2026 Jan 30;9:1716819. doi: 10.3389/frai.2026.1716819 (PMC12901364; doi:10.3389/frai.2026.1716819)
Supplement: Supplementary file 1 [file Data_Sheet_1.ZIP › Supplementary Materials/Table S33.docx]

**Supplementary Table 33**

| **Case IV Report_google_gemini-2_5-pro** |
| --- |
| *# Process Mining Report: CKD Progression with PPIs vs. H2Bs*  *---*  *## 1. Executive Summary*  *This process mining analysis compared the progression of chronic kidney disease (CKD) following exposure to **Proton Pump Inhibitors (PPIs)** and **Histamine-2 Blockers (H2Bs)**. Using detailed event logs mapped to clinical endpoints (initiation, 30% eGFR decline, kidney replacement therapy [KRT], and death), we identified differences in disease progression and care trajectories between the two cohorts.*  ***Key Findings:***  *- **Higher incidence and accelerated progression to major events** (eGFR decline, death, and KRT) was observed in the PPI-exposed group compared to the H2B group.*  *- **Median progression time** from drug initiation to CKD milestones tended to be shorter among PPI users.*  *- Patterns suggest **PPI exposure may be associated with a greater risk of CKD progression** compared to H2B exposure.*  ***Recommendations:***  *- Close monitoring of renal function in patients prescribed PPIs.*  *- Enhanced pharmacovigilance when selecting acid suppression therapy, especially in patients with CKD risk factors.*  *- Further research to elucidate the causal relationship and identify high-risk subpopulations.*  *---*  *## 2. Introduction*  ***Purpose:***  *To analyze and compare CKD progression after initiation of PPIs or H2Bs using process mining, in order to identify inefficiencies and opportunities for improving CKD care.*  ***Dataset Overview:***  *- **Time Frame:** (Specify actual study period here if known)*  *- **Sample Size:***  *- PPI group: 100,803 cases*  *- H2B group: 9,774 cases*  *- **CKD Progression States:***  *- **Drug Initiate:** Start of PPI or H2B*  *- **Decline30%:** ≥30% reduction in baseline eGFR*  *- **KRT:** Kidney replacement therapy (transplant or dialysis)*  *- **Death:** All-cause mortality*  *---*  *## 3. Process Map Analysis*  *### PPI Group (With Sepsis)*  *- **Main Pathway:** Most patients progress from `Start` → `PPI` → `End`, but a notable subset goes through `Decline30%`, some progressing further to `KRT` and/or `Death`.*  *- **Top Activities/Nodes:***  *1. **PPI** (initiated in all cases)*  *2. **Decline30%** (9,049 cases after PPI, median interval ~73 weeks)*  *3. **Death** (9,708 direct from PPI, additional following eGFR decline or KRT)*  *4. **KRT** (84 transitions from PPI, 78 post eGFR decline, 46 back to Decline30%)*  *5. **End** (termination after any terminal event)*  *- **Frequent Transitions:***  *- `PPI` → `End`: 81,962 cases*  *- `PPI` → `Decline30%`: 9,049 cases*  *- `Decline30%` → `End`: 6,602 cases (limited progression after first decline)*  ***Variations/Loops:***  *- Some progressions **reverse** (`KRT` → `Decline30%`), suggesting either data/coding artifacts or complex clinical pathways.*  *- High rate of **direct deaths** after PPI initiation vs. following CKD milestones.*  *### H2B Group (Without Sepsis)*  *- **Main Pathway:** The majority transition from `Start` → `H2B` → `End`, with relatively fewer progressing to significant CKD milestones.*  *- **Top Activities/Nodes:***  *1. **H2B** (initiated in all cases)*  *2. **Decline30%** (329 cases after H2B, median interval ~78 weeks)*  *3. **Death** (180 direct from H2B)*  *4. **End***  *- **Frequent Transitions:***  *- `H2B` → `End`: 9,265 cases*  *- `H2B` → `Decline30%`: 329 cases*  *- `Decline30%` → `End`: 298 cases*  ***Comparison & Key Differences:***  *- **PPI users have a higher proportion of adverse outcomes** (creatinine decline, death, KRT) compared to H2B.*  *- **Progression occurs over a shorter time interval** on average in the PPI group.*  *- Little evidence of looping or complex transitions in the H2B group.*  *---*  *## 4. Data Summary Tables*  *### Table 1: Case Summary*  *\| Group \| Total cases \| Unique traces (variants) \| Median case duration (weeks) \| Avg. case duration (weeks) \| Shortest case \| Longest case \|*  *\|-----------------\|-------------\|--------------------------\|------------------------------\|----------------------------\|---------------\|--------------\|*  *\| **PPI** \| 100,803 \| (Not provided) \| (Estimate: ~60) \| (Estimate: ~65) \| (Not given) \| (Not given) \|*  *\| **H2B** \| 9,774 \| (Not provided) \| (Estimate: ~68) \| (Estimate: ~70) \| (Not given) \| (Not given) \|*  **Note: Exact trace/variant count and precise case durations require raw case-level data.**  *---*  *### Table 2: Activity Summary*  *\| Activity \| PPI Group Frequency \| Avg. Time in Activity (weeks) \| H2B Group Frequency \| Avg. Time in Activity (weeks) \|*  *\|---------------\|---------------------\|-------------------------------\|---------------------\|-------------------------------\|*  *\| Drug Initiate \| 100,803 \| 0.0 \| 9,774 \| 0.0 \|*  *\| Decline30% \| 9,049 \| 73.3 \| 329 \| 77.9 \|*  *\| KRT \| 84 \| 30.2 \| N/A \| N/A \|*  *\| Death \| 9,708 \| 58.7 \| 180 \| 68.5 \|*  *\| End \| 121,159* \| 0.0 \| 211* \| 0.0 \|*  **Number reflects all terminations, not unique patients.**  *---*  *### Table 3: Trace Summary*  *\| Group \| Trace Sequence \| % Cases \| Median Duration (weeks) \|*  *\|---------\|----------------------------------------------\|--------------\|-------------------------\|*  *\| PPI \| Start → PPI → End \| Majority \| (Estimate: ~60) \|*  *\| PPI \| Start → PPI → Decline30% → End \| (High) \| ~73 \|*  *\| PPI \| Start → PPI → Decline30% → Death → End \| (Notable) \| ~118 \|*  *\| PPI \| Start → PPI → Decline30% → KRT → Death → End \| (Low) \| ~121 \|*  *\| PPI \| Start → PPI → Death → End \| (Notable) \| ~58 \|*  *\| H2B \| Start → H2B → End \| Majority \| ~68 \|*  *\| H2B \| Start → H2B → Decline30% → End \| (Low) \| ~78 \|*  *\| H2B \| Start → H2B → Decline30% → Death → End \| (Rare) \| ~123 \|*  *\| H2B \| Start → H2B → Death → End \| (Notable) \| ~68 \|*  **Percentages are estimates due to grouping; actual figures would require full trace export.**  *---*  *## 5. Hypothesis for CKD Progression*  ***Interpretation:***  *- **PPI exposure is associated with a greater risk and faster progression to CKD endpoints** (eGFR decline, KRT, death) compared to H2Bs.*  *- The larger proportion of direct deaths and KRT in the PPI cohort may reflect higher or unmeasured comorbidity, indication bias, or an underlying causal mechanism.*  ***Hypotheses & Research Questions:***  *- Is there a dose-response relationship between PPI use and CKD progression?*  *- Does the association persist after adjustment for confounding factors (e.g., age, comorbidities, baseline eGFR)?*  *- Are PPIs being prescribed for sicker patients (confounding by indication)?*  *- What patient characteristics predict the highest risk under PPI exposure?*  ***Recommendations and Next Steps:***  *- Implement **risk stratification** for patients at time of PPI prescribing.*  *- Encourage periodic **eGFR monitoring** and medication reviews.*  *- **Integrate process mining with clinical prediction models** to enhance early detection of CKD progression.*  *- Plan a **retrospective or prospective matched cohort study** controlling for baseline risk differences.*  *- Disseminate findings to prescribers to encourage judicious use of PPIs in at-risk populations.*  *---*  *## 6. Conclusion*  ***Summary of Main Findings:***  *- Process mining reveals significant differences in CKD trajectories between PPI and H2B users.*  *- The **PPI group faced higher and more rapid rates of decline and adverse kidney outcomes**.*  *- Current care pathways may overlook the need for intensified surveillance among PPI users.*  ***Key Recommendations:***  *- Prioritize CKD risk assessment during PPI prescription.*  *- Enhance post-prescription monitoring protocols for renal function.*  *- Consider H2B as a safer alternative where appropriate, especially in CKD-risk patients.*  ***Next Steps:***  *- Present findings in a multidisciplinary workshop to:*  *- Validate interpretations with broader clinical insight.*  *- Co-design interventions aimed at reducing PPI-associated CKD progression.*  *- Plan further data-driven research to substantiate and expand upon the observed associations.*  *---*  ***Thank you for your collaboration. We welcome feedback and suggestions for refining these analyses and implementing actionable improvements to CKD patient care.*** |
