# Supplementary material for: HealthProcessAI: a technical framework and proof-of-concept for LLM-enhanced healthcare process mining
Source: Front Artif Intell. 2026 Jan 30;9:1716819. doi: 10.3389/frai.2026.1716819 (PMC12901364; doi:10.3389/frai.2026.1716819)
Supplement: Supplementary file 1 [file Data_Sheet_1.ZIP › Supplementary Materials/Table S34.docx]

**Supplementary Table 34**

| **Case IV Report_deepseek_deepseek-r1** |
| --- |
| *# Report: Process Mining Analysis of CKD Progression in Patients Exposed to PPIs vs. H2Bs*  ***To:** Clinical and Epidemiological Stakeholders, CKD Progression Modelling Group*  ***From:** Process Mining Analysis Team*  ***Date:** October 26, 2023*  ***Subject:** Comparative Analysis of Renal Outcomes in Proton Pump Inhibitor (PPI) vs. H2 Blocker (H2B) Users*  *---*  *### 1. Executive Summary*  *This report presents a process mining analysis of Chronic Kidney Disease (CKD) progression in two distinct patient cohorts: those initiated on Proton Pump Inhibitors (PPIs) and those on H2 Blockers (H2Bs). Our analysis reveals significant differences in the clinical journeys of these two groups.*  ***Key Findings:***  ** **More Severe Progression in PPI Cohort:** Patients in the PPI group exhibit more complex and severe disease progression pathways compared to the H2B group. A key differentiator is the presence of **Kidney Replacement Therapy (KRT)** as a clinical outcome, which appears in the PPI cohort but is absent in the H2B cohort based on the analyzed data.*  ** **Higher Incidence of Adverse Outcomes:** The PPI cohort shows a markedly higher proportion of patients experiencing adverse outcomes.*  ** Approximately **9.0%** of PPI users experienced a significant (≥30%) decline in kidney function, compared to **3.4%** of H2B users.*  ** The pathway from drug initiation directly to death was followed by **9.6%** of the PPI cohort, versus only **1.8%** of the H2B cohort.*  ** **Disease Fluctuation:** The analysis of the PPI group identified a clinical loop where patients transitioned from KRT back to a state of significant eGFR decline (`Decline30%`), suggesting periods of disease fluctuation or transitions between different KRT modalities.*  ***Core Recommendation:***  *The data strongly suggests an association between PPI use and a more severe CKD trajectory. However, this may be influenced by underlying differences in patient risk profiles (confounding by indication). We recommend a **follow-up investigation focused on comparing the baseline clinical characteristics** (e.g., age, comorbidities, baseline eGFR) of the two cohorts to determine if the observed differences in outcome are attributable to the medication or pre-existing patient risk.*  *---*  *### 2. Introduction*  *The purpose of this report is to leverage process mining to visualize and analyze the real-world clinical journeys of patients with CKD, specifically comparing the progression pathways for individuals prescribed PPIs versus H2Bs. By mapping these journeys, we aim to identify common patterns, variations, and potential areas for clinical intervention to improve patient outcomes.*  *The analysis is based on longitudinal health data for two patient cohorts. The PPI cohort includes **100,803** patients, while the H2B cohort includes **9,774** patients. The progression of CKD was modeled using four key clinical states:*  ** **Drug Initiate:** The starting point, marking the first prescription of a PPI or H2B.*  ** **Decline30%:** A significant decline in kidney function, defined as a 30% or more reduction in estimated glomerular filtration rate (eGFR) from baseline.*  ** **KRT:** Initiation of Kidney Replacement Therapy, which includes dialysis or kidney transplantation.*  ** **Death:** All-cause mortality recorded during the follow-up period.*  *---*  *### 3. Process Map Analysis*  *The process maps for the two groups reveal starkly different clinical stories. While a majority of patients in both groups do not experience a recorded adverse renal event, the pathways for those who do are significantly different.*  *#### PPI Cohort Pathway*  *The journey for patients on PPIs is more complex and shows a higher frequency of adverse events.*  ** **Most Common Activities:** The most frequent events in this process are **PPI Initiation** (100,803 instances), followed by **Death** (12,159 instances) and **Decline30%** (9,049 instances).*  ** **Main Patient Flows:***  *1. **No Recorded Adverse Event:** The most common path is `PPI -> End` (81,962 cases, 81.3%), where patients are initiated on the drug and do not have a subsequent adverse event recorded in the dataset.*  *2. **Progression to Death:** A significant number of patients follow the path `PPI -> Death` (9,708 cases, 9.6%). The average time for this transition is **58.7 weeks**.*  *3. **Decline in Kidney Function:** The path `PPI -> Decline30%` (9,049 cases, 9.0%) is also prominent, with an average time of **73.3 weeks** (approx. 1.4 years) to this event. From here, patients either die, stabilize, or progress to KRT.*  ** **Significant Variation:** The most critical finding is the presence of the **KRT** state. Patients progress from both initial PPI use and `Decline30%` to KRT. We also observe a loop, `KRT -> Decline30%`, indicating potential instability or transitions in care for patients on KRT.*  *#### H2B Cohort Pathway*  *The H2B cohort follows a much simpler and more favorable process, with significantly fewer adverse events.*  ** **Most Common Activities:** The main events are **H2B Initiation** (9,774 instances), followed by **Decline30%** (329 instances) and **Death** (211 instances).*  ** **Main Patient Flows:***  *1. **No Recorded Adverse Event:** The overwhelming majority of patients follow the path `H2B -> End` (9,265 cases, 94.8%).*  *2. **Decline in Kidney Function:** Far fewer patients experience a drop in kidney function. The path `H2B -> Decline30%` occurred in 329 cases (3.4%), with an average transition time of **77.9 weeks**.*  ** **Key Difference:** The complete **absence of the KRT state** in the H2B cohort's process map is the most striking difference. This suggests that progression to end-stage renal disease requiring KRT is either non-existent or exceptionally rare in this group within the observed timeframe.*  *---*  *### 4. Data Summary Tables*  *The following tables provide a quantitative summary of the cases, activities, and common pathways (traces) for both cohorts.*  **Note: Case duration is calculated by summing the average time between activities for a given pathway. Time spent *in* an activity is not available; the table instead shows the average time leading *to* an activity.**  *#### **Table 1: Case Summary***  *\| Metric \| PPI Cohort \| H2B Cohort \|*  *\| :--- \| :--- \| :--- \|*  *\| **Total Number of Cases** \| 100,803 \| 9,774 \|*  *\| **Number of Unique Traces** \| >8 \| 4 \|*  *\| **Median Case Duration (Est.)** \| ~60 weeks \| ~70 weeks \|*  *\| **Shortest Case Duration** \| 0 weeks (`... -> End`) \| 0 weeks (`... -> End`) \|*  *\| **Longest Case Duration (Est.)**\| >150 weeks \| ~124 weeks \|*  *#### **Table 2: Activity Summary***  *\| Group \| Activity \| Frequency (# of times) \| Avg. Time from Previous Activity (Weeks) \|*  *\| :--- \| :--- \| :--- \| :--- \|*  *\| **PPI** \| PPI Initiate \| 100,803 \| 0 \|*  *\| \| Decline30% \| 9,049 \| 73.3 \|*  *\| \| KRT \| 162 \| 30.2 (from PPI), 51.1 (from Decline30%) \|*  *\| \| Death \| 12,159 \| 58.7 (from PPI), 45.3 (from Decline30%) \|*  *\| **H2B** \| H2B Initiate \| 9,774 \| 0 \|*  *\| \| Decline30% \| 329 \| 77.9 \|*  *\| \| Death \| 211 \| 68.5 (from H2B), 46.0 (from Decline30%) \|*  *#### **Table 3: Top Process Variants (Traces)***  ***PPI Cohort - Top 4 Traces***  *\| Rank \| Process Variant \| % of Cases \| Median Duration (Weeks, Est.) \|*  *\| :--- \| :--- \| :---: \| :---: \|*  *\| 1 \| `Start -> PPI -> End` \| 81.3% \| 0 \|*  *\| 2 \| `Start -> PPI -> Death -> End` \| 9.6% \| 59 \|*  *\| 3 \| `Start -> PPI -> Decline30% -> End` \| 6.5% \| 73 \|*  *\| 4 \| `Start -> PPI -> Decline30% -> Death -> End` \| 2.4% \| 119 \|*  ***H2B Cohort - All 4 Traces***  *\| Rank \| Process Variant \| % of Cases \| Median Duration (Weeks, Est.) \|*  *\| :--- \| :--- \| :---: \| :---: \|*  *\| 1 \| `Start -> H2B -> End` \| 94.8% \| 0 \|*  *\| 2 \| `Start -> H2B -> Decline30% -> End` \| 3.0% \| 78 \|*  *\| 3 \| `Start -> H2B -> Death -> End` \| 1.8% \| 68 \|*  *\| 4 \| `Start -> H2B -> Decline30% -> Death -> End` \| 0.3% \| 124 \|*  *---*  *### 5. Hypothesis for CKD Progression*  *This analysis provides a powerful visualization of CKD progression but does not establish causality. The findings lead to several hypotheses and critical research questions.*  ***Interpretations & Hypotheses:***  *1. **Confounding by Indication (Most Likely Hypothesis):** The stark differences may not be caused by the drugs themselves but by the underlying health status of the patients receiving them. Patients prescribed PPIs are often older, have more comorbidities (e.g., cardiovascular disease, diabetes), or are on other medications (like NSAIDs) that independently increase CKD risk. In essence, the PPI cohort may simply be a sicker, higher-risk population at baseline.*  *2. **Direct Drug Effect (Alternative Hypothesis):** While less likely to explain the entire effect, it remains possible that PPIs contribute to kidney injury through known mechanisms (e.g., acute interstitial nephritis, hypomagnesemia) and that this analysis visualizes the cumulative, long-term clinical impact of such effects.*  ***Proposed Research Questions:***  ** What are the baseline differences in age, sex, comorbidities (diabetes, hypertension, heart failure), and initial eGFR between the PPI and H2B cohorts?*  ** After adjusting for these baseline differences using statistical models, does an association between PPI use and adverse renal outcomes persist?*  ** What clinical factors predict which PPI users will progress to a `Decline30%`? The average time of **~73 weeks** to this event represents a critical window for potential intervention.*  ***Recommendations for CKD Prediction:***  ** **Enrich Data:** The next step should be to enrich this process data with patient-level clinical features to test the confounding hypothesis.*  ** **Develop Predictive Models:** A predictive model could be developed to identify PPI users at the highest risk of progression. By combining process sequence data with clinical markers, we could flag patients who warrant closer eGFR monitoring or consideration for alternative acid-suppressive therapy.*  *---*  *### 6. Conclusion*  *This process mining analysis has successfully illuminated the divergent clinical pathways of CKD patients initiated on PPIs versus H2Bs. The findings clearly show that the PPI cohort is associated with a higher burden of adverse renal outcomes, including progression to KRT, which was not observed in the H2B cohort.*  ***Key Recommendations Summary:***  *1. **Investigate Confounding:** Prioritize a comparative analysis of the baseline clinical and demographic characteristics of the two cohorts to understand pre-existing risk.*  *2. **Targeted Monitoring:** Use these findings to advocate for vigilant monitoring of kidney function in patients on long-term PPI therapy, especially those with other known risk factors for CKD.*  *3. **Predictive Analytics:** Explore the development of a predictive risk model to proactively identify high-risk individuals within the large population of PPI users.*  ***Next Steps:***  *We propose hosting a **collaborative workshop** with the clinical and epidemiological teams. The goal of this workshop would be to:*  ** Review these findings in detail and gather clinical interpretations.*  ** Validate the observed pathways against clinical experience.*  ** Co-design the next phase of analysis, focusing on adjusting for confounders and building a robust predictive model for CKD progression.* |
