# Supplementary material for: HealthProcessAI: a technical framework and proof-of-concept for LLM-enhanced healthcare process mining
Source: Front Artif Intell. 2026 Jan 30;9:1716819. doi: 10.3389/frai.2026.1716819 (PMC12901364; doi:10.3389/frai.2026.1716819)
Supplement: Supplementary file 1 [file Data_Sheet_1.ZIP › Supplementary Materials/Table S35.docx]

**Supplementary Table 35**

| **Case IV Report_x-ai_grok-4** |
| --- |
| *# Process Mining Analysis Report: Association Between PPI and H2B Exposure and CKD Progression*  *## Executive Summary*  *This report analyzes CKD progression pathways using process mining techniques, comparing patients exposed to Proton Pump Inhibitors (PPIs) versus Histamine-2 Blockers (H2Bs). Key findings reveal that the PPI group exhibits higher frequencies of adverse outcomes, including a 9% rate of 30% eGFR decline (Decline30%) and 12% all-cause mortality (Death), compared to 3.4% and 2.2% in the H2B group, respectively. Additionally, average times to these events are slightly shorter in the PPI group (e.g., 73 weeks to Decline30% vs. 78 weeks in H2B). Kidney Replacement Therapy (KRT) occurs only in the PPI group, albeit rarely (0.16% of cases), with evidence of loops between KRT and Decline30%, indicating complex progression patterns not seen in H2B. These differences suggest a potential association between PPI exposure and accelerated CKD progression.*  *Recommendations include prioritizing closer monitoring for PPI-exposed patients, exploring alternative therapies like H2Bs for at-risk individuals, and integrating predictive modeling to forecast progression risks. Next steps involve a collaborative workshop to validate findings and refine care protocols, aiming to improve patient outcomes and efficiency in CKD management.*  *## Introduction*  *The purpose of this report is to analyze CKD progression using process mining to identify inefficiencies and opportunities for improvement in patient care pathways. By comparing exposure to Proton Pump Inhibitors (PPIs) versus Histamine-2 Blockers (H2Bs), we assess associations with CKD progression, highlighting differences in event frequencies, timings, and pathways to inform clinical decision-making and epidemiological modeling.*  *The analysis is based on two process matrices derived from event log data. The PPI cohort includes 100,803 cases, and the H2B cohort includes 9,774 cases. The data spans an unspecified time frame but captures longitudinal progression in weeks, focusing on key events in CKD. CKD progression is modeled according to the following states:*  *- **Drug Initiate**: Start date for Proton Pump Inhibitors (PPI) or Histamine-2 Blockers (H2B).*  *- **Decline30%**: 30% or more reduction in baseline kidney function (eGFR).*  *- **KRT**: Kidney Replacement Therapy (includes transplant and dialysis, as per Swedish Renal Registry).*  *- **Death**: All-cause mortality.*  *Process mining reveals the sequences, frequencies, and durations of these events, enabling a comparison of progression dynamics between groups.*  *## Process Map Analysis*  *The process maps for both cohorts illustrate CKD progression as sequences starting from drug initiation, followed by potential adverse events (Decline30%, KRT, Death) or censoring (End). The main pathway in both groups is drug initiation followed by no further events (censoring), reflecting the majority of cases without progression. However, significant differences emerge in event frequencies, timings, and complexities.*  *In the **PPI cohort**, the most common pathway is PPI initiation directly to End (81.3% of cases), indicating censoring without progression. Key variations include paths to Death (9.6%) and Decline30% (9%), with rare paths to KRT (0.08% directly from PPI). A notable complexity is the presence of loops between KRT and Decline30% (e.g., KRT → Decline30% in 46 instances, Decline30% → KRT in 78 instances), suggesting repeated declines or therapy adjustments in a small subset, deviating from expected linear CKD progression. This loop is absent in the H2B group, potentially indicating more unstable kidney function in PPI-exposed patients.*  *In the **H2B cohort**, the main pathway is similarly H2B initiation to End (94.8%), but with lower progression rates: Decline30% in 3.4% and Death in 1.8%. No KRT events or loops are observed, resulting in simpler, more linear paths.*  ***Top 3-5 Most Frequent Activities (Nodes) and Their Roles**:*  *- **Drug Initiate (PPI or H2B)**: Appears in all cases as the starting point, marking exposure onset. Role: Entry into the observation period.*  *- **Decline30%**: Frequent marker of CKD worsening (9,095 occurrences in PPI vs. 329 in H2B). Role: Indicates significant eGFR drop, often leading to further events.*  *- **Death**: Common terminal event (12,159 in PPI vs. 211 in H2B). Role: All-cause mortality endpoint.*  *- **KRT** (PPI only): Rare (162 occurrences). Role: Advanced intervention for severe CKD.*  *- **End**: Artificial node signaling case conclusion (censoring or after terminal event).*  ***Most Common Transitions and Frequencies** (highlighting differences):*  *- PPI/H2B → End: 81,962 (81.3%) in PPI vs. 9,265 (94.8%) in H2B – dominant censoring path, lower in PPI due to higher events.*  *- PPI/H2B → Death: 9,708 (9.6%) in PPI vs. 180 (1.8%) in H2B – higher mortality transition in PPI (average 59 weeks vs. 68 weeks).*  *- PPI/H2B → Decline30%: 9,049 (9%) in PPI vs. 329 (3.4%) in H2B – more frequent and faster progression in PPI (73 weeks vs. 78 weeks).*  *- Decline30% → Death: 2,415 (2.4% of total) in PPI vs. 31 (0.3%) in H2B – similar timings (45 weeks).*  *- PPI-specific: Decline30% → KRT (78) and KRT → Decline30% (46) – loops absent in H2B, suggesting PPI-linked instability.*  *These differences highlight potential inefficiencies in the PPI group, such as higher progression rates and complex loops, which could inform targeted interventions to streamline care and reduce adverse outcomes.*  *## Data Summary Tables*  *### Table 1: Case Summary*  ***PPI Cohort***  *\| Metric \| Value \|*  *\|--------\|-------\|*  *\| Total number of cases \| 100,803 \|*  *\| Number of unique traces (variants) \| 12 \|*  *\| Median case duration \| 66 weeks \|*  *\| Average case duration \| 70 weeks \|*  *\| Duration of the shortest case \| 0 weeks \|*  *\| Duration of the longest case \| 250 weeks \|*  ***H2B Cohort***  *\| Metric \| Value \|*  *\|--------\|-------\|*  *\| Total number of cases \| 9,774 \|*  *\| Number of unique traces (variants) \| 4 \|*  *\| Median case duration \| 74 weeks \|*  *\| Average case duration \| 76 weeks \|*  *\| Duration of the shortest case \| 0 weeks \|*  *\| Duration of the longest case \| 250 weeks \|*  *### Table 2: Activity Summary*  ***PPI Cohort***  *\| Activity \| Frequency \| Median Time Spent (weeks) \| Average Time Spent (weeks) \|*  *\|----------\|-----------\|---------------------------\|----------------------------\|*  *\| PPI \| 100,803 \| 66 \| 65.55 \|*  *\| Decline30% \| 9,095 \| 45 \| 45.46 \|*  *\| KRT \| 162 \| 35 \| 35.25 \|*  *\| Death \| 12,159 \| 0 \| 0 \|*  ***H2B Cohort***  *\| Activity \| Frequency \| Median Time Spent (weeks) \| Average Time Spent (weeks) \|*  *\|----------\|-----------\|---------------------------\|----------------------------\|*  *\| H2B \| 9,774 \| 75 \| 74.55 \|*  *\| Decline30% \| 329 \| 46 \| 45.98 \|*  *\| Death \| 211 \| 0 \| 0 \|*  *### Table 3: Trace Summary*  ***PPI Cohort***  *\| Rank \| Trace (Variant) \| Percentage of Cases \| Median Duration (weeks) \|*  *\|------\|-----------------\|---------------------\|-------------------------\|*  *\| 1 \| PPI → End \| 81.3% \| 66 \|*  *\| 2 \| PPI → Death → End \| 9.6% \| 59 \|*  *\| 3 \| PPI → Decline30% → End \| 6.5% \| 119 \|*  *\| 4 \| PPI → Decline30% → Death → End \| 2.4% \| 119 \|*  *\| 5 \| PPI → KRT → End \| 0.04% \| 65 \|*  ***H2B Cohort** (Note: Only 4 variants identified; no 5th variant)*  *\| Rank \| Trace (Variant) \| Percentage of Cases \| Median Duration (weeks) \|*  *\|------\|-----------------\|---------------------\|-------------------------\|*  *\| 1 \| H2B → End \| 94.8% \| 75 \|*  *\| 2 \| H2B → Decline30% → End \| 3.05% \| 124 \|*  *\| 3 \| H2B → Death → End \| 1.84% \| 68 \|*  *\| 4 \| H2B → Decline30% → Death → End \| 0.32% \| 124 \|*  *\| 5 \| N/A \| 0% \| N/A \|*  *## Hypothesis for CKD Progression*  *The process maps indicate that PPI exposure is associated with more frequent and potentially faster CKD progression compared to H2B, as evidenced by higher rates of Decline30% (9% vs. 3.4%), Death (12% vs. 2.2%), and exclusive occurrence of KRT and related loops in the PPI group. These patterns suggest PPIs may exacerbate renal stress, leading to accelerated eGFR decline and increased mortality risk, while H2B pathways appear more stable with fewer adverse events.*  ***Proposed Hypotheses and Research Questions**:*  *- **Hypothesis 1**: PPI exposure increases the hazard ratio for eGFR decline by at least 2-fold compared to H2B, mediated by gastrointestinal-renal axis mechanisms.*  *- **Hypothesis 2**: The KRT-Decline30% loops in PPI patients reflect recurrent acute kidney injuries, absent in H2B due to differing pharmacodynamics.*  *- **Research Question 1**: What patient subgroups (e.g., by age, comorbidities) show the strongest PPI-associated progression risks?*  *- **Research Question 2**: Can machine learning models incorporating process mining traces predict time-to-Decline30% within 12 months accuracy?*  ***Recommendations and Next Steps for CKD Prediction**:*  *- Implement risk-stratified monitoring for PPI patients, such as quarterly eGFR checks for those with baseline risks.*  *- Consider H2B as a preferred alternative for acid suppression in CKD-prone populations to reduce progression events.*  *- Develop predictive tools integrating process mining with survival analysis to forecast individualized CKD trajectories within 6-12 months, using features like time-to-first-event.*  *- Next steps: Conduct Cox proportional hazards modeling on the full dataset to quantify risks, followed by validation with external cohorts.*  *## Conclusion*  *This analysis demonstrates clear differences in CKD progression between PPI and H2B-exposed patients, with PPIs linked to higher frequencies of eGFR decline, mortality, and KRT, alongside complex loops indicating potential inefficiencies in care pathways. In contrast, H2B pathways are simpler and associated with lower event rates, suggesting a protective or less harmful profile.*  *Key recommendations emphasize enhanced monitoring for PPI patients, therapy alternatives, and predictive modeling to optimize outcomes. As next steps, we suggest organizing a workshop with the clinical and epidemiological team to discuss these findings, validate interpretations, and co-design targeted interventions for improved patient care and operational efficiency.* |
