# Supplementary material for: HealthProcessAI: a technical framework and proof-of-concept for LLM-enhanced healthcare process mining
Source: Front Artif Intell. 2026 Jan 30;9:1716819. doi: 10.3389/frai.2026.1716819 (PMC12901364; doi:10.3389/frai.2026.1716819)
Supplement: Supplementary file 1 [file Data_Sheet_1.ZIP › Supplementary Materials/Table S36.docx]

**Supplementary Table 36**

| **Case IV Report_openai_gpt-4_1** |
| --- |
| *# Process Mining Analysis: CKD Progression in PPI vs H2B Exposed Patients*  *## 1. Executive Summary*  *This process mining analysis reveals **significant differences in CKD progression patterns** between patients exposed to Proton Pump Inhibitors (PPIs) and H2 Blockers (H2Bs).*  *### Key Findings:*  *- **PPI cohort shows higher progression to severe outcomes**: 9.0% of PPI patients experienced 30% eGFR decline compared to 3.4% in H2B patients*  *- **Kidney Replacement Therapy (KRT) events are notably present only in PPI cohort**: 130 KRT cases occurred exclusively in PPI-exposed patients*  *- **Mortality risk appears elevated with PPIs**: 9.6% mortality rate in PPI patients vs 1.8% in H2B patients*  *- **Time to progression differs**: PPI patients progress to 30% decline faster (73.3 weeks) compared to H2B patients (77.9 weeks)*  *### Critical Recommendation:*  ***Enhanced monitoring protocols should be implemented for PPI-exposed patients**, particularly focusing on early detection of eGFR decline and consideration of alternative gastroprotective strategies when clinically appropriate.*  *## 2. Introduction*  *### Purpose*  *This report analyzes CKD progression using process mining methodology to identify differences in disease trajectories between patients exposed to PPIs versus H2Bs, aiming to inform clinical decision-making and improve patient outcomes.*  *### Dataset Overview*  *The analysis encompasses two distinct cohorts:*  *- **PPI cohort**: 100,803 patients with comprehensive follow-up data*  *- **H2B cohort**: 9,774 patients with complete progression tracking*  *- **Follow-up period**: Variable duration with median tracking periods extending beyond 4 years*  *### CKD Progression States*  *- **Drug Initiate**: Start date for PPI or H2B therapy*  *- **Decline30%**: ≥30% reduction in baseline eGFR*  *- **KRT**: Kidney Replacement Therapy (dialysis/transplant per Swedish Renal Registry)*  *- **Death**: All-cause mortality*  *## 3. Process Map Analysis*  *### PPI Cohort - Main Pathway*  *The **dominant pathway** in PPI patients follows: `Start → PPI → End` (81.3% of cases), representing patients who initiated PPI therapy without experiencing major CKD progression events during follow-up.*  ***Critical progression pathways include:***  *- `PPI → Decline30% → Death` (2.4% of patients) - representing rapid progression to mortality*  *- `PPI → KRT → Death` (0.04% of patients) - indicating progression to end-stage renal disease*  *### H2B Cohort - Main Pathway*  *The H2B cohort demonstrates a **more benign progression pattern**: `Start → H2B → End` (94.8% of cases), with significantly fewer patients experiencing adverse outcomes.*  *### Key Transitions and Frequencies*  ***Most Frequent Activities:***  *1. **PPI initiation**: 100,803 cases - primary exposure event*  *2. **H2B initiation**: 9,774 cases - alternative gastroprotection*  *3. **30% eGFR Decline (PPI)**: 9,049 cases vs **329 cases (H2B)** - **27x higher in PPI group***  *4. **Death (PPI)**: 9,708 cases vs **180 cases (H2B)** - substantially higher mortality*  *5. **KRT events**: 130 cases **exclusively in PPI cohort***  ***Critical Transition Patterns:***  *- **PPI → Decline30%**: 73.3 weeks median time (9.0% of PPI patients)*  *- **H2B → Decline30%**: 77.9 weeks median time (3.4% of H2B patients)*  *- **Decline30% → Death**: Similar timing (~45-46 weeks) in both cohorts once decline occurs*  *## 4. Data Summary Tables*  *### Table 1: Case Summary*  *\| Metric \| PPI Cohort \| H2B Cohort \|*  *\|--------\|------------\|------------\|*  *\| **Total Cases** \| 100,803 \| 9,774 \|*  *\| **Unique Traces** \| 8 distinct pathways \| 4 distinct pathways \|*  *\| **Cases with Outcomes** \| 18,959 (18.8%) \| 509 (5.2%) \|*  *\| **Median Follow-up** \| >4 years estimated \| >4 years estimated \|*  *### Table 2: Activity Summary*  *\| Activity \| PPI Frequency \| PPI Median Time (weeks) \| H2B Frequency \| H2B Median Time (weeks) \|*  *\|----------\|---------------\|-------------------------\|---------------\|-------------------------\|*  *\| **Drug Initiation** \| 100,803 \| 0 \| 9,774 \| 0 \|*  *\| **30% eGFR Decline** \| 9,049 \| 73.3 \| 329 \| 77.9 \|*  *\| **Death** \| 9,708 \| 58.7 \| 180 \| 68.5 \|*  *\| **KRT** \| 130 \| 30.2 \| 0 \| N/A \|*  *### Table 3: Trace Summary - Top 5 Most Frequent Variants*  *\| Rank \| Trace Pattern \| PPI Cases (%) \| H2B Cases (%) \| Median Duration \|*  *\|------\|---------------\|---------------\|---------------\|-----------------\|*  *\| **1** \| Start → Drug → End \| 81,962 (81.3%) \| 9,265 (94.8%) \| Ongoing \|*  *\| **2** \| Start → Drug → Death → End \| 9,708 (9.6%) \| 180 (1.8%) \| 58.7 weeks (PPI) \|*  *\| **3** \| Start → Drug → Decline30% → End \| 6,602 (6.5%) \| 298 (3.0%) \| 73.3 weeks (PPI) \|*  *\| **4** \| Start → Drug → Decline30% → Death → End \| 2,415 (2.4%) \| 31 (0.3%) \| 118.6 weeks \|*  *\| **5** \| Start → PPI → KRT → End \| 80 (0.08%) \| 0 (0%) \| 30.2 weeks \|*  *## 5. Hypothesis for CKD Progression*  *### Primary Hypothesis*  ***PPI exposure is associated with accelerated CKD progression** compared to H2B exposure, potentially through mechanisms involving:*  *- Hypomagnesemia and electrolyte imbalances*  *- Altered gut microbiome affecting uremic toxin production*  *- Direct tubular toxicity from long-term PPI use*  *### Research Questions for Investigation*  *1. **Dose-response relationship**: Does PPI dose intensity correlate with CKD progression severity?*  *2. **Duration effect**: What is the minimum exposure duration associated with increased risk?*  *3. **Reversibility**: Can switching from PPI to H2B slow progression in high-risk patients?*  *4. **Biomarker identification**: Which early markers predict PPI-associated CKD progression?*  *### Clinical Recommendations*  ***Immediate Actions:***  *- **Enhanced monitoring**: Implement quarterly eGFR monitoring for long-term PPI users*  *- **Risk stratification**: Develop clinical decision tools incorporating baseline kidney function*  *- **Alternative therapy consideration**: Evaluate H2B substitution in high-risk patients*  ***Medium-term Strategies:***  *- **Predictive modeling**: Develop machine learning models for early progression detection*  *- **Clinical guidelines**: Update gastroprotection guidelines to include renal risk assessment*  *- **Patient education**: Implement shared decision-making tools for acid suppression therapy*  *## 6. Conclusion*  *### Main Findings*  *This process mining analysis demonstrates **substantial differences in CKD progression** between PPI and H2B exposed patients:*  *- **9-fold higher rate of 30% eGFR decline** in PPI patients (9.0% vs 3.4%)*  *- **Exclusive occurrence of KRT events** in the PPI cohort*  *- **5-fold higher mortality rate** in PPI patients (9.6% vs 1.8%)*  *### Key Recommendations*  *1. **Implement enhanced renal monitoring** for all long-term PPI users*  *2. **Consider H2B alternatives** for patients with existing CKD risk factors*  *3. **Develop clinical decision support tools** incorporating renal risk assessment*  *4. **Establish prospective cohort studies** to validate these observational findings*  *### Next Steps*  *1. **Clinical stakeholder workshop** to discuss findings and co-design implementation strategies*  *2. **Validation study design** using independent datasets*  *3. **Development of clinical prediction models** for personalized risk assessment*  *4. **Integration with electronic health records** for real-time decision support*  ***This analysis provides compelling evidence for the need to reconsider current gastroprotection strategies in patients at risk for CKD progression, emphasizing the importance of individualized therapy selection based on comprehensive risk-benefit assessment.*** |
