# Supplementary material for: HealthProcessAI: a technical framework and proof-of-concept for LLM-enhanced healthcare process mining
Source: Front Artif Intell. 2026 Jan 30;9:1716819. doi: 10.3389/frai.2026.1716819 (PMC12901364; doi:10.3389/frai.2026.1716819)
Supplement: Supplementary file 1 [file Data_Sheet_1.ZIP › Supplementary Materials/Table S37.docx]

**Supplementary Table 37**

| **Case IV Report_qwen_qwen-2_5-72b-instruct** |
| --- |
| *# Process Mining Analysis Report: CKD Progression and Proton Pump Inhibitors (PPIs) vs. Histamine-2 Blockers (H2Bs)*  *## 1. Executive Summary*  *This report provides a comprehensive analysis of the association between exposure to Proton Pump Inhibitors (PPIs) and Histamine-2 Blockers (H2Bs) and the progression of Chronic Kidney Disease (CKD). Using process mining, we identified key differences in CKD progression pathways between the two drug groups. The main findings include:*  *- **PPI Exposure Group:***  *- Higher incidence of CKD progression to a 30% decline in eGFR and KRT.*  *- Longer average case duration and more frequent transitions to adverse outcomes.*  *- **H2B Exposure Group:***  *- Lower incidence of CKD progression to a 30% decline in eGFR and KRT.*  *- Shorter average case duration and fewer transitions to adverse outcomes.*  ***Key Recommendations:***  *- Conduct further research to validate the observed differences.*  *- Implement targeted monitoring and intervention strategies for patients on PPIs.*  *- Explore alternative treatment options for patients at high risk of CKD progression.*  *## 2. Introduction*  *The purpose of this report is to analyze the progression of CKD using process mining to identify inefficiencies and opportunities for improvement. The dataset used for this analysis includes patients exposed to either PPIs or H2Bs. The time frame of the data spans several years, and the number of cases analyzed is substantial.*  *CKD progression has been modeled according to the following states:*  *- **Drug Initiate** - Start date for Proton Pump Inhibitors (PPI) or Histamine-2 Blockers (H2B).*  *- **Decline30%** - 30% or more reduction in baseline kidney function (eGFR).*  *- **KRT** - Kidney Replacement Therapy (includes transplant and dialysis, as per Swedish Renal Registry).*  *- **Death** - All-cause mortality.*  *## 3. Process Map Analysis*  *### PPI Exposure Group*  *- **Main Pathway:***  *- The most common pathway starts with PPI initiation, followed by a decline in eGFR, and often ends with KRT or death.*  *- **Most Frequent Activities:***  *- **PPI Initiate**: 100,803 occurrences*  *- **End**: 81,962 occurrences*  *- **Decline30%**: 9,049 occurrences*  *- **Death**: 9,708 occurrences*  *- **KRT**: 84 occurrences*  *- **Common Transitions:***  *- **PPI → Decline30%**: 9,049 occurrences, average time 73.27 weeks*  *- **PPI → Death**: 9,708 occurrences, average time 58.66 weeks*  *- **PPI → End**: 81,962 occurrences, average time 0 weeks*  *- **Decline30% → Death**: 2,415 occurrences, average time 45.28 weeks*  *- **Decline30% → KRT**: 78 occurrences, average time 51.14 weeks*  *### H2B Exposure Group*  *- **Main Pathway:***  *- The most common pathway starts with H2B initiation, followed by a decline in eGFR, and often ends with death or a non-event (End).*  *- **Most Frequent Activities:***  *- **H2B Initiate**: 9,774 occurrences*  *- **End**: 9,265 occurrences*  *- **Decline30%**: 329 occurrences*  *- **Death**: 180 occurrences*  *- **Common Transitions:***  *- **H2B → Decline30%**: 329 occurrences, average time 77.87 weeks*  *- **H2B → Death**: 180 occurrences, average time 68.49 weeks*  *- **H2B → End**: 9,265 occurrences, average time 0 weeks*  *- **Decline30% → Death**: 31 occurrences, average time 45.98 weeks*  *## 4. Data Summary Tables*  *### Table 1: Case Summary*  *\| **Metric** \| **PPI Exposure Group** \| **H2B Exposure Group** \|*  *\|--------------------------------\|------------------------\|------------------------\|*  *\| **Total Number of Cases** \| 100,803 \| 9,774 \|*  *\| **Number of Unique Traces** \| 4 \| 3 \|*  *\| **Median Case Duration (Weeks)** \| 0 \| 0 \|*  *\| **Average Case Duration (Weeks)** \| 58.66 \| 68.49 \|*  *\| **Shortest Case Duration (Weeks)** \| 0 \| 0 \|*  *\| **Longest Case Duration (Weeks)** \| 73.27 \| 77.87 \|*  *### Table 2: Activity Summary*  *\| **Activity** \| **PPI Exposure Group** \| **H2B Exposure Group** \|*  *\|--------------\|------------------------\|------------------------\|*  *\| **Frequency** \| **Median Time (Weeks)** \| **Average Time (Weeks)** \| **Frequency** \| **Median Time (Weeks)** \| **Average Time (Weeks)** \|*  *\| **PPI Initiate** \| 100,803 \| 0 \| 0 \| 9,774 \| 0 \| 0 \|*  *\| **End** \| 81,962 \| 0 \| 0 \| 9,265 \| 0 \| 0 \|*  *\| **Decline30%** \| 9,049 \| 0 \| 45.28 \| 329 \| 0 \| 45.98 \|*  *\| **Death** \| 9,708 \| 0 \| 58.66 \| 180 \| 0 \| 68.49 \|*  *\| **KRT** \| 84 \| 0 \| 30.21 \| 0 \| 0 \| 0 \|*  *### Table 3: Trace Summary*  *\| **Trace** \| **PPI Exposure Group** \| **H2B Exposure Group** \|*  *\|-----------------------------------\|------------------------\|------------------------\|*  *\| **Trace** \| **Percentage of Cases** \| **Median Duration (Weeks)** \| **Percentage of Cases** \| **Median Duration (Weeks)** \|*  *\| **PPI → End** \| 81.3% \| 0 \| **H2B → End** \| 94.8% \| 0 \|*  *\| **PPI → Decline30% → Death** \| 2.4% \| 45.28 \| **H2B → Decline30% → Death** \| 0.3% \| 45.98 \|*  *\| **PPI → Decline30% → KRT** \| 0.1% \| 51.14 \| **H2B → Decline30%** \| 3.4% \| 77.87 \|*  *\| **PPI → Death** \| 9.6% \| 58.66 \| **H2B → Death** \| 1.8% \| 68.49 \|*  *\| **PPI → Decline30% → End** \| 6.6% \| 0 \| **H2B → End** \| 94.8% \| 0 \|*  *## 5. Hypothesis for CKD Progression*  *### Interpretation of Findings*  *- **PPI Exposure Group:***  *- Patients on PPIs have a higher likelihood of experiencing a 30% decline in eGFR and progressing to KRT or death.*  *- The longer average case duration and more frequent transitions to adverse outcomes suggest a more rapid and severe CKD progression.*  *- **H2B Exposure Group:***  *- Patients on H2Bs have a lower likelihood of experiencing a 30% decline in eGFR and progressing to KRT or death.*  *- The shorter average case duration and fewer transitions to adverse outcomes suggest a slower and less severe CKD progression.*  *### New Hypotheses and Research Questions*  *1. **Mechanistic Hypothesis:** Investigate the underlying mechanisms by which PPIs may contribute to more rapid CKD progression compared to H2Bs.*  *2. **Risk Stratification:** Develop a risk stratification model to identify patients at high risk of CKD progression based on PPI exposure.*  *3. **Intervention Strategies:** Explore targeted monitoring and intervention strategies for patients on PPIs to mitigate the risk of CKD progression.*  *### Recommendations and Next Steps*  *1. **Further Research:** Conduct longitudinal studies to validate the observed differences and explore the underlying biological mechanisms.*  *2. **Clinical Guidelines:** Update clinical guidelines to include monitoring and intervention strategies for patients on PPIs.*  *3. **Patient Education:** Educate patients on the potential risks of PPIs and the importance of regular kidney function monitoring.*  *4. **Alternative Treatments:** Investigate and promote the use of alternative treatments for patients at high risk of CKD progression.*  *## 6. Conclusion*  *### Main Findings*  *- Patients on PPIs have a higher incidence of CKD progression to a 30% decline in eGFR and KRT, with longer average case durations and more frequent transitions to adverse outcomes.*  *- Patients on H2Bs have a lower incidence of CKD progression, with shorter average case durations and fewer transitions to adverse outcomes.*  *### Key Recommendations*  *- Conduct further research to validate the observed differences and explore underlying mechanisms.*  *- Implement targeted monitoring and intervention strategies for patients on PPIs.*  *- Update clinical guidelines and educate patients on the risks and benefits of PPIs.*  *### Next Steps*  *- Organize a workshop with the clinical team to discuss the findings and co-design solutions.*  *- Initiate a pilot study to test the effectiveness of targeted monitoring and intervention strategies.*  *- Collaborate with researchers to conduct longitudinal studies and explore alternative treatments.* |
