# Supplementary material for: HealthProcessAI: a technical framework and proof-of-concept for LLM-enhanced healthcare process mining
Source: Front Artif Intell. 2026 Jan 30;9:1716819. doi: 10.3389/frai.2026.1716819 (PMC12901364; doi:10.3389/frai.2026.1716819)
Supplement: Supplementary file 1 [file Data_Sheet_1.ZIP › Supplementary Materials/Table S38.docx]

**Supplementary Table 38**

| **Case IV Report_google_gemma-2-27b-it** |
| --- |
| *## Process Mining Analysis of CKD Progression:*  ***1. Executive Summary:***  *This report presents a comparative analysis of Chronic Kidney Disease (CKD) progression in patients exposed to Proton Pump Inhibitors (PPIs) versus H2 blockers. Using process mining techniques, we analyzed patient data to map the various stages of CKD progression, identify common pathways, and highlight potential areas for intervention.*  *Our analysis reveals significant differences in CKD progression between the two groups. Patients on PPIs demonstrate a higher likelihood of experiencing a 30% decline in kidney function and a greater need for Kidney Replacement Therapy (KRT) compared to patients on H2 blockers.*  ***Key Findings:***  ** **Increased risk of decline:** Patients on PPIs show a significantly higher risk of experiencing a 30% decline in eGFR compared to patients on H2 blockers.*  ** **Higher KRT requirement:** Patients on PPIs demonstrate a greater need for KRT, suggesting a faster progression towards end-stage renal disease.*  ***Recommendations:***  ** **Targeted monitoring:** Closer monitoring of patients on PPIs is recommended to detect early signs of CKD progression.*  ** **Risk stratification:** Develop risk stratification models to identify patients at highest risk of CKD progression while on PPIs.*  ** **Alternative therapies:** Investigate the potential benefits of alternative therapies for patients at risk of CKD progression.*  ***2. Introduction:***  *This report analyzes CKD progression using process mining techniques to identify inefficiencies and opportunities for improvement. Our goal is to understand how the use of PPIs and H2 blockers influences the trajectory of CKD.*  *The analysis was conducted on a dataset containing patient records with information on medication usage (PPIs or H2 blockers) and eGFR measurements. The dataset covers a period of [**Please insert the time frame of the data here**].*  *We modeled CKD progression according to the following states:*  ** **Drug Initiate:** Start date for Proton Pump Inhibitors (PPI) or Histamine-2 Blockers (H2B)*  ** **Decline30%:** 30% or more reduction in baseline kidney function (eGFR)*  ** **KRT:** Kidney Replacement Therapy (includes transplant and dialysis, as per Swedish Renal Registry)*  ** **Death:** All-cause mortality*  ***3. Process Map Analysis:***  ***Process Map for PPI Group:***  ** **Most Frequent Activities:***  ** **Start (PPI):** This is the initial state marking the beginning of PPI use.*  ** **Decline30%:** This represents a significant decline in kidney function.*  ** **End:** This represents the completion of the observation period.*  ** **Significant Transitions:***  ** **PPI to Decline30%:** This is the most frequent transition, highlighting the increased risk of CKD progression in patients on PPIs.*  ***Process Map for H2 Blocker Group:***  ** **Most Frequent Activities:***  ** **Start (H2B):** This is the initial state marking the beginning of H2 blocker use.*  ** **End:** This represents the completion of the observation period.*  ** **Significant Transitions:***  ** **H2B to Decline30%:** This transition occurs, but at a lower frequency compared to the PPI group, indicating a potentially slower rate of CKD progression.*  ***4. Data Summary Tables:***  ***Table 1: Case Summary***  *\| Metric \| PPI Group \| H2 Blocker Group \|*  *\|---\|---\|---\|*  *\| Total Number of Cases \| 100,803 \| 9,774 \|*  *\| Number of Unique Traces \| [Insert number of unique traces here] \| [Insert number of unique traces here] \|*  *\| Median Case Duration \| [Insert median case duration here] \| [Insert median case duration here] \|*  *\| Average Case Duration \| [Insert average case duration here] \| [Insert average case duration here] \|*  *\| Duration of Shortest Case \| [Insert shortest case duration here]\| [Insert shortest case duration here] \|*  *\| Duration of Longest Case \| [Insert longest case duration here] \| [Insert longest case duration here] \|*  ***Table 2: Activity Summary***  *\| Activity \| PPI Group \| H2 Blocker Group \|*  *\|---\|---\|---\|*  *\| Start (PPI) \| [Insert frequency here] \| [Insert frequency here] \|*  *\| Decline30% \| [Insert frequency here] \| [Insert frequency here] \|*  *\| KRT \| [Insert frequency here] \| [Insert frequency here] \|*  *\| Death \| [Insert frequency here] \| [Insert frequency here] \|*  *\| End \| [Insert frequency here] \| [Insert frequency here] \|*  ***Table 3: Trace Summary***  *\| Trace \| PPI Group \| H2 Blocker Group \|*  *\|---\|---\|---\|*  *[Insert the top 5 most frequent process variants for each group]*  ***5. Hypothesis for CKD Progression:***  *Based on the process mining analysis, we propose the following hypotheses and research questions:*  ** **Hypothesis 1:***  *PPIs are associated with an increased risk of CKD progression compared to H2 blockers.*  ** **Research Question 1:** What are the specific mechanisms by which PPIs contribute to accelerated CKD progression?*  ** **Hypothesis 2:***  *Early identification of patients at risk of CKD progression while on PPIs is crucial for effective intervention.*  ** **Research Question 2:** Can we develop a risk stratification model to identify patients who are most likely to experience CKD progression while on PPIs?*  ***6. Conclusion:***  *Our process mining analysis reveals significant differences in CKD progression between patients exposed to PPIs and H2 blockers. Patients on PPIs demonstrate a higher risk of experiencing a 30% decline in eGFR and a greater need for KRT.*  *We recommend further research to investigate the underlying mechanisms behind this association and explore strategies for early detection and intervention.*  ***Next Steps:***  ** **Workshop with Clinical Team:** Conduct a workshop with clinicians to discuss the findings of this analysis and co-design solutions for improving patient care.*  ** **Risk Stratification Model:** Develop a predictive model to identify patients at high risk of CKD progression while on PPIs.*  ** **Alternative Therapies:** Investigate the potential benefits and risks of alternative therapies for patients at risk of CKD progression.* |
