# Supplementary material for: HealthProcessAI: a technical framework and proof-of-concept for LLM-enhanced healthcare process mining
Source: Front Artif Intell. 2026 Jan 30;9:1716819. doi: 10.3389/frai.2026.1716819 (PMC12901364; doi:10.3389/frai.2026.1716819)
Supplement: Supplementary file 1 [file Data_Sheet_1.ZIP › Supplementary Materials/Table S39.docx]

**Supplementary Table 39**

| **Case IV Report_meta-llama_llama-3_1-70b-instruct** |
| --- |
| *Here is the report:*  ***Executive Summary***  *This report presents the findings of a process mining analysis on the association between exposure to Proton Pump Inhibitors (PPIs) and Chronic Kidney Disease (CKD) progression in comparison to exposure to Histamine-2 Blockers (H2Bs). The analysis reveals significant differences in the progression of CKD between patients exposed to PPIs and H2Bs. The report highlights areas for improvement and provides recommendations for enhancing patient care and operational efficiency.*  ***Introduction***  *The purpose of this report is to analyze CKD progression using process mining to identify inefficiencies and opportunities for improvement. The dataset used for the analysis consists of two process matrices: one for patients exposed to PPIs and another for patients exposed to H2Bs. The dataset includes 100,803 cases for PPIs and 9,774 cases for H2Bs. The time frame of the data is not specified. CKD progression has been modeled according to the following states: "Drug Initiate" - start date for Proton Pump Inhibitors (PPI) or Histamine-2 Blockers (H2B); "Decline30%" - 30% or more reduction in baseline kidney function (eGFR); "KRT" - Kidney Replacement Therapy (includes transplant and dialysis, as per Swedish Renal Registry); "Death" - all-cause mortality.*  ***Process Map Analysis***  *The process map for PPIs reveals a complex pathway with multiple transitions between activities. The most frequent activities are:*  ** "PPI" (100,803 occurrences)*  ** "Decline30%" (9,049 occurrences)*  ** "End" (81,962 occurrences)*  ** "KRT" (84 occurrences)*  *The most common transitions are:*  ** "PPI" to "Decline30%" (73.27 weeks)*  ** "PPI" to "End" (0 weeks)*  ** "Decline30%" to "KRT" (30.21 weeks)*  *In contrast, the process map for H2Bs shows a simpler pathway with fewer transitions. The most frequent activities are:*  ** "H2B" (9,774 occurrences)*  ** "Decline30%" (329 occurrences)*  ** "End" (9,265 occurrences)*  *The most common transitions are:*  ** "H2B" to "Decline30%" (77.87 weeks)*  ** "H2B" to "End" (0 weeks)*  ***Data Summary Tables***  ***Table 1: Case Summary***  *\| Metric \| PPIs \| H2Bs \|*  *\| --- \| --- \| --- \|*  *\| Total number of cases \| 100,803 \| 9,774 \|*  *\| Number of unique traces (variants) \| \| \|*  *\| Median case duration \| \| \|*  *\| Average case duration \| \| \|*  *\| Duration of the shortest case \| \| \|*  *\| Duration of the longest case \| \| \|*  ***Table 2: Activity Summary***  *\| Activity \| PPIs \| H2Bs \|*  *\| --- \| --- \| --- \|*  *\| PPI \| 100,803 (73.27 weeks) \| \|*  *\| H2B \| \| 9,774 (77.87 weeks) \|*  *\| Decline30% \| 9,049 (73.27 weeks) \| 329 (77.87 weeks) \|*  *\| KRT \| 84 (30.21 weeks) \| \|*  *\| End \| 81,962 (0 weeks) \| 9,265 (0 weeks) \|*  *\| DEATH \| 9708 (58.66 weeks) \| 180 (68.49 weeks) \|*  ***Table 3: Trace Summary***  *\| Trace \| PPIs \| H2Bs \|*  *\| --- \| --- \| --- \|*  *\| Top 5 most frequent process variants \| \| \|*  *\| Percentage of cases that follow each trace \| \| \|*  *\| Median duration of each trace \| \| \|*  ***Hypothesis for CKD Progression***  *Based on the process mining analysis, we propose the following hypothesis:*  ** Patients exposed to PPIs have a higher risk of CKD progression compared to patients exposed to H2Bs.*  ** The pathway for PPIs is more complex, with multiple transitions between activities, indicating a higher risk of adverse outcomes.*  *We recommend further research to validate these findings and to explore the underlying mechanisms driving CKD progression in patients exposed to PPIs and H2Bs.*  ***Conclusion***  *In conclusion, this report highlights significant differences in the progression of CKD between patients exposed to PPIs and H2Bs. The process mining analysis reveals a more complex pathway for PPIs, with a higher risk of adverse outcomes. We recommend further research to validate these findings and to explore the underlying mechanisms driving CKD progression. We also suggest next steps, such as a workshop with the clinical team to discuss the findings and co-design solutions to enhance patient care and operational efficiency.*  *Note: The tables are not fully populated as the data is not provided.* |
