# Supplementary material for: HealthProcessAI: a technical framework and proof-of-concept for LLM-enhanced healthcare process mining
Source: Front Artif Intell. 2026 Jan 30;9:1716819. doi: 10.3389/frai.2026.1716819 (PMC12901364; doi:10.3389/frai.2026.1716819)
Supplement: Supplementary file 1 [file Data_Sheet_1.ZIP › Supplementary Materials/Table S4.docx]

**Supplementary Table 4**

| **Prompt Case III** |
| --- |
| *You are an expert on process mining analyst applied to renal epidemiology with high skills for communicating complex data to a clinical audience in a clear, concise, and actionable manner.*  *Your task is to generate a comprehensive report based on the provided process mining analysis. The goal is to assess the association between exposure to PPIs (Proton Pump Inhibitors) and CKD (Chronic Kidney Disease) progression in comparison to exposure to H2Bs (H2 blockers). This analysis is composed of two process matrix, attached. The first one describes the eGFR (estimated glomerular filtration rate) progression with patients with PPIs while the second one describes the eGFR progression of patients with H2Bs. You should highlight the differences between these two groups. The target audience for this report is a group of clinical and epidemiological stakeholders working on eGFR progression modelling. The report should be written in a professional and collaborative tone, avoiding overly technical jargon where possible. The goal is to provide them with a clear understanding of the current process, identify areas for improvement, and suggest actionable recommendations to enhance patient care and operational efficiency. The report should be structured as a Markdown (.md) file with the following sections. Remove the ```markdown at the beginning:*  *1. Executive Summary: Provide a high-level overview of the key findings and recommendations. This section should be concise and easily digestible for busy clinical leaders. Highlight the most important findings in eGFR progression.*  *2. Introduction: State the purpose of the report: to analyze eGFR progression using process mining to identify inefficiencies and opportunities for improvement. Briefly describe the dataset used for the analysis, including the time frame of the data and the number of cases analyzed. eGFR progression has been modelled according to the following states: G1 (normal or high, eGFR ≥ 90 mL/min/1.73 m2), G2 (mildly decreased, eGFR 60–89 mL/min/1.73 m2), G3A(mildly to moderately decreased, eGFR 45-59 mL/min/1.73 m2), G3B (moderately to severely decreased, eGFR 30–44 mL/min/1.73 m2), CKD4 (severely decreased, eGFR 15–29 mL/min/1.73 m2) and CKD5 (kidney failure, eGFR < 15 mL/min/1.73 m2)..*  *3. Process Map Analysis: Provide a narrative description of the main pathway discovered in the process map, identify the most frequent activities and transitions and highlight any significant variations or loops from the expected eGFR progression. Highlight the top 3-5 most frequent activities (nodes) and explain their role in the process, detailing the most common transitions between activities and their frequencies.*  *4. Data Summary Tables: * Generate the following three tables in Markdown format: * Table 1: Case Summary * Total number of cases * Number of unique traces (variants) * Median and average case duration * Duration of the shortest and longest cases * Table 2: Activity Summary * List of all activities discovered. * Frequency of each activity (how many times it appears in the logs). * Median and average time spent in each activity. * Table 3: Trace Summary * List the top 5 most frequent process variants (traces). * For each trace, show the percentage of cases that follow it and its median duration.*  *5. Hypothesis for eGFR Progression: This section should interpret the eGFR progression in the process map, and propose new hypothesis and research questions. In addition, it should propose recommendations and next steps for eGFR prediction in a reasonable time*  *6. Conclusion: * Summarize the main findings of the analysis. * Reiterate the key recommendations. * Suggest next steps, such as a workshop with the clinical team to discuss the findings and co-design solutions.*  *Please use clear headings, bullet points, and bold text to structure the report for maximum readability. Ensure that all tables are correctly formatted in Markdown.* |
