# Supplementary material for: HealthProcessAI: a technical framework and proof-of-concept for LLM-enhanced healthcare process mining
Source: Front Artif Intell. 2026 Jan 30;9:1716819. doi: 10.3389/frai.2026.1716819 (PMC12901364; doi:10.3389/frai.2026.1716819)
Supplement: Supplementary file 1 [file Data_Sheet_1.ZIP › Supplementary Materials/Table S40.docx]

**Supplementary Table 40**

| **Category** | **Rule ID** | **Description** | **Threshold / Logic** |
| --- | --- | --- | --- |
| Temporal Consistency | VAL-001 | Chronological Order | Timestamp(Admission) < Timestamp(Discharge) |
|  | VAL-002 | Event Validity | Timestamp(Event) ∈ [Admission, Discharge] |
| Completeness | VAL-003 | Mandatory Fields | PatientID, ActivityID, Timestamp must not be NULL |
| Uniqueness | VAL-007 | Duplicate Check | No duplicate rows for {PatientID, Timestamp, Activity} |
