# Supplementary material for: HealthProcessAI: a technical framework and proof-of-concept for LLM-enhanced healthcare process mining
Source: Front Artif Intell. 2026 Jan 30;9:1716819. doi: 10.3389/frai.2026.1716819 (PMC12901364; doi:10.3389/frai.2026.1716819)
Supplement: Supplementary file 1 [file Data_Sheet_1.ZIP › Supplementary Materials/Table S41.docx]

**Supplementary Table 41**

| **Infection Progression** |
| --- |
| *Initialize empty list: Activity_List*  *# Iterate through each unique patient/subject in the dataset*  *FOR EACH unique Subject in data:*  *# Reset state flags for the new subject*  *# Note: 'Has_Sepsis' acts as a latch; once True, it stays True for the subject*  *SET Has_Sepsis = FALSE*    *# Extract all rows belonging to the current Subject*  *SET Subject_Data = rows in data where case == Subject*  *# Iterate through each time-step/record for this subject*  *FOR EACH Record in Subject_Data:*  *# -------------------------------------------------------*  *# 1. CHECK SEPSIS STATUS (Highest Priority)*  *# -------------------------------------------------------*  *IF (Record.SepsisLabel IS 1) OR (Has_Sepsis IS TRUE):*  *SET Has_Sepsis = TRUE*  *APPEND "Sepsis" to Activity_List*    *# -------------------------------------------------------*  *# 2. IF NO SEPSIS, DETERMINE INFECTION & TEMPERATURE*  *# -------------------------------------------------------*  *ELSE:*  *# A. Check for Infection based on White Blood Cell (WBC) count*  *# Infection is defined as WBC > 11 or WBC < 4.5*  *IF (Record.WBC > 11) OR (Record.WBC < 4.5):*  *SET Is_Infected = TRUE*  *ELSE:*  *SET Is_Infected = FALSE*  *# B. Check Temperature Category*  *IF Record.Temp < 36:*  *SET Temp_State = "Low Temperature"*  *ELSE IF 36 <= Record.Temp < 37.5:*  *SET Temp_State = "Normal Temperature"*  *ELSE: # Record.Temp >= 37.5*  *SET Temp_State = "High Temperature"*  *# C. Define Final Activity String*  *IF Is_Infected IS TRUE:*  *# Combine Infection status with Temperature status*  *APPEND "Infection + " + Temp_State to Activity_List*  *ELSE:*  *# Only record Temperature status*  *APPEND Temp_State to Activity_List*  *END FOR (Record)*  *END FOR (Subject)*  *RETURN Activity_List* |
