# Supplementary material for: HealthProcessAI: a technical framework and proof-of-concept for LLM-enhanced healthcare process mining
Source: Front Artif Intell. 2026 Jan 30;9:1716819. doi: 10.3389/frai.2026.1716819 (PMC12901364; doi:10.3389/frai.2026.1716819)
Supplement: Supplementary file 1 [file Data_Sheet_1.ZIP › Supplementary Materials/Table S42.docx]

**Supplementary Table 41**

| **Infection Progression** |
| --- |
| *Initialize empty list: Activity_List*  *# Iterate through each unique patient/subject in the dataset*  *FOR EACH unique Subject in data:*  *# Reset latch flags for the new subject*  *# These flags persist (latch) once set to TRUE*  *SET Is_Cardiac = FALSE*  *SET Is_Renal = FALSE*  *SET Is_Liver = FALSE*  *SET Is_Multidamage = FALSE*  *SET Has_Sepsis = FALSE*    *# Extract all rows belonging to the current Subject*  *SET Subject_Data = rows in data where case == Subject*  *# Iterate through each time-step/record for this subject*  *FOR EACH Record in Subject_Data:*  *# -------------------------------------------------------*  *# 1. CHECK SEPSIS STATUS (Highest Priority)*  *# -------------------------------------------------------*  *IF (Record.SepsisLabel IS 1) OR (Has_Sepsis IS TRUE):*  *SET Has_Sepsis = TRUE*  *APPEND "Sepsis" to Activity_List*    *# -------------------------------------------------------*  *# 2. IF NO SEPSIS, DETERMINE ORGAN DAMAGE*  *# -------------------------------------------------------*  *ELSE:*    *# A. Check Cardiac Damage (TroponinI) [Latch]*  *IF (Record.TroponinI > 0.04) OR (Is_Cardiac IS TRUE):*  *SET Is_Cardiac = TRUE*  *ELSE:*  *SET Is_Cardiac = FALSE*    *# B. Check Renal Damage (Creatinine) [Latch]*  *IF (Record.Creatinine > 1.3) OR (Is_Renal IS TRUE):*  *SET Is_Renal = TRUE*  *ELSE:*  *SET Is_Renal = FALSE*    *# C. Check Liver Damage (AST) [Latch]*  *IF (Record.AST > 40) OR (Is_Liver IS TRUE):*  *SET Is_Liver = TRUE*  *ELSE:*  *SET Is_Liver = FALSE*  *# D. Check Multiorgan Damage [Latch]*  *# Defined as strictly more than 2 organs damaged (implies all 3)*  *SET Damaged_Count = (Is_Cardiac as INT) + (Is_Renal as INT) + (Is_Liver as INT)*    *IF (Damaged_Count > 2) OR (Is_Multidamage IS TRUE):*  *SET Is_Multidamage = TRUE*  *ELSE:*  *SET Is_Multidamage = FALSE*  *# E. Define Activity String based on Priority*  *IF Is_Multidamage IS TRUE:*  *APPEND "Multiorgan Damage" to Activity_List*    *ELSE IF (Is_Liver IS TRUE) AND (Is_Cardiac IS TRUE):*  *APPEND "Liver + Cardiac Damage" to Activity_List*    *ELSE IF (Is_Liver IS TRUE) AND (Is_Renal IS TRUE):*  *APPEND "Liver + Renal Damage" to Activity_List*    *ELSE IF (Is_Renal IS TRUE) AND (Is_Cardiac IS TRUE):*  *APPEND "Renal + Cardiac Damage" to Activity_List*    *ELSE IF Is_Liver IS TRUE:*  *APPEND "Liver Damage" to Activity_List*    *ELSE IF Is_Cardiac IS TRUE:*  *APPEND "Cardiac Damage" to Activity_List*    *ELSE IF Is_Renal IS TRUE:*  *APPEND "Renal Damage" to Activity_List*    *ELSE:*  *APPEND "Low Risk" to Activity_List*  *END FOR (Record)* |
