# Supplementary material for: HealthProcessAI: a technical framework and proof-of-concept for LLM-enhanced healthcare process mining
Source: Front Artif Intell. 2026 Jan 30;9:1716819. doi: 10.3389/frai.2026.1716819 (PMC12901364; doi:10.3389/frai.2026.1716819)
Supplement: Supplementary file 1 [file Data_Sheet_1.ZIP › Supplementary Materials/Table S6.docx]

**Supplementary Table 6**

| **Prompt for Evaluation** |
| --- |
| *"# LLM Process Mining Report Evaluation Prompt ## Task You are an expert evaluator for healthcare process mining reports. You will evaluate reports generated by different LLM models analyzing sepsis patient pathways. Score each report based on the provided rubric criteria. ## Evaluation Rubric (Score 1-4 for each criterion)*  *### 1. RELEVANCE - 4 (Exemplary): Fully addresses key clinical and contextual issues. Strong alignment with process map and report purpose. - 3 (Proficient): Addresses most relevant issues; minor gaps in alignment or scope. - 2 (Needs Improvement): Covers topic broadly but misses core clinical/contextual focus or the process framework. - 1 (Insufficient): Misaligned with clinical context; key issues not addressed.*  *### 2. STRUCTURE & PRESENTATION - 4 (Exemplary): Clear, logical organization with defined sections. Effective use of tables/figures to support interpretation. - 3 (Proficient): Generally well-structured; visuals used but may lack consistency or clarity. - 2 (Needs Improvement): Structure exists but is disjointed or difficult to follow. Visual aids are underused or unclear. - 1 (Insufficient): No discernible structure. Unformatted text and no visual supports.*  *### 3. UNDERSTANDABILITY - 4 (Exemplary): Clear, concise, jargon-free language. Accessible to a broad range of stakeholders. - 3 (Proficient): Mostly clear with minor technical or dense sections. - 2 (Needs Improvement): Some sections are unclear or inconsistent in tone and terminology. - 1 (Insufficient): Poorly written throughout; impedes understanding.*  *### 4. COMPLETENESS - 4 (Exemplary): Comprehensive coverage of components: interpretation steps, clinical pathways, and KPIs. - 3 (Proficient): Most components included but may lack depth in some areas. - 2 (Needs Improvement): Overview is present, but omits critical interpretive elements or performance metrics. - 1 (Insufficient): Lacks essential content. Missing interpretation or KPI references.*  *### 5. INNOVATION - 4 (Exemplary): Demonstrates creative approaches or novel clinical insights beyond standard practice. - 3 (Proficient): Shows elements of creativity or innovation; may lack full development. - 2 (Needs Improvement): Limited originality; relies on conventional methods without new perspectives. - 1 (Insufficient): No evidence of innovation; basic, derivative, or rote output.*  *### 6. ACCURACY - 4 (Exemplary): Clinically and contextually accurate. Terminology and figures aligned with process map and domain standards. - 3 (Proficient): Mostly accurate with minor issues that don't affect the core message, or just fail in calculating some figures that are not feasible for a LLM. - 2 (Needs Improvement): Noticeable errors in clinical interpretation, terms, or figure use. - 1 (Insufficient): Major inaccuracies or misinterpretations compromising validity.*  *## Evaluation Process For each model's report, provide: 1. Individual Criterion Scores (1-4 for each of the 6 criteria) 2. Brief Justification for each score (1-2 sentences) 3. Key Strengths (2-3 bullet points) 4. Key Weaknesses (2-3 bullet points) 5. Specific Examples of excellence or issues from the report 6. Overall Assessment (2-3 sentences) 7. Average Score (calculate mean of 6 criteria) ## Important Evaluation Guidelines ### What to Look For: - Clinical Accuracy: Check medical terminology, disease progression logic, treatment pathways - Process Mining Understanding: Verify correct interpretation of transitions, states, frequencies - Statistical Claims: Note any specific numbers that seem unrealistic or unverifiable - Consistency: Check if numbers and claims are consistent throughout the report - Actionability: Assess if recommendations are practical and evidence-based - Target Audience: Consider if the report is appropriate for clinical and epidemiological stakeholders*  *### Red Flags to Identify: - Fabricated statistics or percentages without source - Contradictory statements within the same report - Misinterpretation of process mining concepts - Unrealistic clinical claims or treatment suggestions - Poor understanding of sepsis progression - Missing critical components (KPIs, recommendations, etc.)*  *## Output Format Please provide your evaluation in this structured format: MODEL: [Model Name] CASE: [Case Number and Description] SCORES: - Relevance: [1-4] - [Brief justification] - Structure & Presentation: [1-4] - [Brief justification] - Understandability: [1-4] - [Brief justification] - Completeness: [1-4] - [Brief justification] - Innovation: [1-4] - [Brief justification] - Accuracy: [1-4] - [Brief justification] AVERAGE SCORE: [X.X/4.0] KEY STRENGTHS: • [Strength 1] • [Strength 2] • [Strength 3] KEY WEAKNESSES: • [Weakness 1] • [Weakness 2] • [Weakness 3] SPECIFIC EXAMPLES: • Excellence: [Quote or description with location] • Issue: [Quote or description with location] OVERALL ASSESSMENT: [2-3 sentence summary of the report's quality and suitability for healthcare decision-making] ---*  *## Models to Evaluate Please evaluate the following models' reports for each case: 1. Anthropic Claude (anthropic_sonnet-4) 2. DeepSeek R1 (deepseek_deepseek-r1) 3. Google Gemini (google_gemini-2_5-pro) 4. OpenAI GPT-4 (openai_gpt-4_1) 5. Grok 4 (x-ai_grok-4) 6. Qwen 2.5 72 b (qwen/qwen-2.5-72b-instruct) 7. Google Gemma 2 (google/gemma-2-27b-it) 8. Meta Llama (meta-llama/llama-3.1-70b-instruct)*  *## Cases to Evaluate - Case I: Infection Progression - Case II: Organ Damage - Case III: Glomerular Filtration Rate - Case IV: Kidney Disease Progression ## Additional Context These reports were generated from process mining analysis of sepsis patient data. The process maps show patient state transitions (e.g., temperature changes, infection status, organ dysfunction) and their frequencies. Reports should: - Interpret the process maps accurately - Provide clinical insights - Suggest actionable improvements - Be suitable for healthcare professionals and administrators*  *## Validation Questions to Consider 1. Are the stated numbers (transitions, frequencies, durations) plausible? 2. Do the clinical interpretations align with known sepsis pathophysiology? 3. Are the recommendations evidence-based and implementable? 4. Does the report demonstrate understanding of process mining concepts? 5. Is the language appropriate for the target audience? 6. Are there any obvious hallucinations or fabricated claims? --- Please begin your evaluation with the reports provided. Be objective, thorough, and provide specific examples to support your scores."* |
