# Supplementary material for: HealthProcessAI: a technical framework and proof-of-concept for LLM-enhanced healthcare process mining
Source: Front Artif Intell. 2026 Jan 30;9:1716819. doi: 10.3389/frai.2026.1716819 (PMC12901364; doi:10.3389/frai.2026.1716819)
Supplement: Supplementary file 1 [file Data_Sheet_1.ZIP › Supplementary Materials/Table S7.docx]

**Supplementary Table 8**

| **Case I Report_google_gemini-2_5-pro** |
| --- |
| *# Sepsis Progression Analysis: A Process Mining Perspective*  *### **To:** Sepsis Progression Modelling Stakeholders*  *### **From:** Process Mining Analysis Team*  *### **Date:** October 26, 2023*  *### **Subject:** Analysis of Sepsis Patient Pathways and Opportunities for Early Intervention*  *---*  *## 1. Executive Summary*  *This report presents a process mining analysis of 1,206 patient cases culminating in a sepsis diagnosis. Our analysis reveals the critical pathways and bottlenecks in sepsis progression, providing data-driven insights to support the development of predictive models.*  ***Key Findings:***  ** **Dominant Process Feature:** The most frequent behavior observed is a rapid and recurring fluctuation between **`Normal Temperature`** and **`High Temperature`**. This temperature volatility appears to be a central feature in the lead-up to sepsis.*  ** **Primary Pathway to Sepsis:** The most common direct precursor to a sepsis diagnosis is the **`High Temperature`** state. This transition (`High Temperature` -> `Sepsis`) is notable for its relatively long average duration of **7.1 hours**, suggesting a potential window for intervention that may be currently missed.*  ** **Key Infection Gateway:** The transition from **`High Temperature`** to **`Infection + High Temperature`** is the most common entry point into a state of confirmed infection. This progression takes an average of **6.1 hours**.*  ** **Actionable Insight:** The intense cycling between normal and high temperatures represents a critical, early warning signal. By focusing on patients exhibiting this pattern, we can potentially identify at-risk individuals before they progress to more severe states.*  ***Key Recommendations:***  *1. **Develop a Temperature Volatility Alert:** Create a monitoring protocol or automated alert for patients who cycle between `Normal Temperature` and `High Temperature` multiple times within a short period.*  *2. **Investigate the 7-Hour `High Temperature` to `Sepsis` Delay:** Conduct a clinical review of cases following this pathway to understand if the 7.1-hour duration represents a physiological process or a delay in diagnosis and treatment.*  *3. **Prioritize the `High Temperature` State:** Treat the `High Temperature` state as a critical decision point where patients either recover to normal, develop a confirmed infection, or progress directly to sepsis.*  *---*  *## 2. Introduction*  *The purpose of this report is to leverage process mining to analyze the progression of sepsis in patients, with the goal of identifying common pathways, process inefficiencies, and opportunities for earlier intervention. By visualizing and quantifying patient journeys, we aim to provide actionable insights for the development of more accurate sepsis prediction models.*  *The analysis is based on event data from **1,206 patient cases** that concluded with a sepsis diagnosis. The data models sepsis progression through five core, reversible states:*  ** i) `Low Temperature`*  ** ii) `Normal Temperature`*  ** iii) `High Temperature`*  ** iv) `Infection` (which is always combined with a temperature state, e.g., `Infection + High Temperature`)*  ** v) `Sepsis`*  *This analysis illuminates the real-world sequences of these states, their frequencies, and the time elapsed between them.*  *---*  *## 3. Process Map Analysis*  *The process map, derived from the provided data, reveals a highly dynamic and interconnected process. Instead of a single, linear path to sepsis, we observe a core process loop with several key "exit ramps" toward severe outcomes.*  *The dominant feature of the process is the **strong, bidirectional flow between `Normal Temperature` and `High Temperature`**.*  ** `Normal Temperature` -> `High Temperature`: Occurred **14,940 times**.*  ** `High Temperature` -> `Normal Temperature`: Occurred **14,492 times**.*  *This suggests that patients often experience significant temperature instability, cycling back and forth between these two states before their condition either resolves or deteriorates. This "temperature chattering" is the most frequent activity pattern in the entire dataset and represents the main "highway" of the patient journey.*  ***Top 5 Most Frequent Activities (States):***  *1. **`High Temperature` (19,806 observations):** This is the most central and frequently visited state in the process. It acts as a critical junction. From here, patients most commonly revert to `Normal Temperature`, but it is also the primary gateway to `Infection + High Temperature` and, most critically, to `Sepsis`.*  *2. **`Normal Temperature` (16,209 observations):** This state serves as the baseline. The most significant transition from here is the progression to `High Temperature`, which happens quickly (average 1.4 hours).*  *3. **`Infection + High Temperature` (3,003 observations):** This is the most common state indicating a confirmed infection. It is most often entered from a `High Temperature` state and most frequently transitions back to `High Temperature` (without the infection flag), suggesting that treatment may be addressing the infection but temperature instability persists.*  *4. **`Low Temperature` (2,175 observations):** A less frequent but important state. Patients in this state most commonly transition to `High Temperature`, indicating a potential hypothermic precursor to a febrile response.*  *5. **`Sepsis` (1,206 observations):** This is the final absorbing state for all cases in this dataset. It is most frequently preceded by `High Temperature`.*  ***Significant Variations and Loops:***  ** **The Main Loop:** `Normal Temperature` <-> `High Temperature`. The sheer volume of transitions indicates this is where patients spend a significant amount of time before a definitive diagnosis.*  ** **The Infection Loop:** `High Temperature` -> `Infection + High Temperature` -> `High Temperature`. This secondary loop suggests that once an infection is present, it is tightly coupled with a high temperature state.*  *---*  *## 4. Data Summary Tables*  ***A Note on Data Availability:** The provided process matrix contains aggregated transition data. This allows for powerful analysis of flows between states but does not contain the full, end-to-end journey for each individual case. Therefore, metrics like case duration and the time spent *within* an activity cannot be calculated. The tables below reflect the available data.*  *### **Table 1: Case Summary***  *\| Metric \| Value \|*  *\| :--- \| :--- \|*  *\| **Total Number of Cases** \| **1,206** \|*  *\| Number of Unique Traces (Variants) \| Not Available from Provided Data \|*  *\| Median Case Duration \| Not Available from Provided Data \|*  *\| Average Case Duration \| Not Available from Provided Data \|*  *\| Duration of Shortest/Longest Case \| Not Available from Provided Data \|*  *### **Table 2: Activity Summary***  **Note: Time spent *in* each activity is not available. Frequencies are based on the number of times a state was entered.**  *\| Activity (State) \| Frequency (Times Entered) \| Median/Average Time in Activity \|*  *\| :--- \| :--- \| :--- \|*  *\| **High Temperature** \| **19,806** \| Not Available \|*  *\| **Normal Temperature** \| **16,209** \| Not Available \|*  *\| **Infection + High Temperature** \| 3,003 \| Not Available \|*  *\| **Low Temperature** \| 2,175 \| Not Available \|*  *\| **Sepsis** \| 1,206 \| Not Available \|*  *\| **Infection + Normal Temperature** \| 1,103 \| Not Available \|*  *\| **Infection + Low Temperature** \| 250 \| Not Available \|*  *### **Table 3: Top 5 Most Frequent Transitions***  **This table replaces a "Trace Summary" to highlight the most common steps in the patient journey based on the available data.**  *\| Rank \| Transition (From -> To) \| Frequency \| Average Duration (Hours) \|*  *\| :--- \| :--- \| :--- \| :--- \|*  *\| 1 \| `Normal Temperature` -> `High Temperature` \| 14,940 \| 1.4 \|*  *\| 2 \| `High Temperature` -> `Normal Temperature` \| 14,492 \| 3.9 \|*  *\| 3 \| `Infection + High Temperature` -> `High Temperature` \| 2,206 \| 1.3 \|*  *\| 4 \| `High Temperature` -> `Infection + High Temperature` \| 2,167 \| 6.1 \|*  *\| 5 \| `Low Temperature` -> `High Temperature` \| 1,672 \| 1.1 \|*  *---*  *## 5. Hypothesis for Sepsis Progression*  *Based on the process analysis, we can formulate several hypotheses and research questions to guide the development of predictive models.*  ***Hypotheses:***  *1. **Temperature volatility is a primary predictor of risk.** The high-frequency cycling between `Normal` and `High` temperature is not random noise but a key physiological signal of a body fighting and failing to achieve homeostasis, indicating a higher risk of progression to sepsis.*  *2. **The transition from `High Temperature` to `Sepsis` represents a critical, time-sensitive intervention window.** The average duration of 7.1 hours for this transition is one of the longest in the process. This may represent a "last chance" period where the patient's condition is visibly deteriorating but has not yet been formally coded as sepsis. Understanding the clinical events within this window is paramount.*  *3. **There are two distinct major pathways to sepsis:***  ** **Path A (Direct):** `Temperature Instability` -> `High Temperature` -> `Sepsis`. This appears to be the most common path.*  ** **Path B (Infection-Mediated):** `Temperature Instability` -> `High Temperature` -> `Infection + High Temperature` -> `Sepsis`. This path involves a state of confirmed infection before the final sepsis diagnosis.*  ***Proposed Research Questions:***  ** What clinical factors (e.g., lab values, comorbidities, medications) differentiate patients who remain in the `Normal <-> High` temperature loop from those who exit towards an `Infection` state or `Sepsis`?*  ** Why does the `Normal Temperature` -> `Infection + Normal Temperature` transition take a relatively long time (6.2 hours)? Does this represent a delay in recognizing afebrile infections?*  ** Can we build a predictive model that uses the frequency and amplitude of temperature shifts, rather than a single temperature threshold, as a primary input feature?*  ***Recommendations for Sepsis Prediction:***  ** **Focus on Dynamic Features:** Predictive models should prioritize dynamic features like the *rate of change* and *volatility* of temperature over static, single-point-in-time measurements.*  ** **Time-Sensitive Alerts:** Develop alerts for patients who enter high-risk transitions, particularly:*  ** After a second or third cycle between `Normal` and `High` temperature.*  ** Upon entering the `High Temperature` -> `Sepsis` pathway, flagging them for urgent clinical review.*  ** **Data Enrichment:** For future analysis, we recommend enriching the dataset with lab results (e.g., lactate, CRP, WBC) and medication timings (e.g., antibiotics) to understand the clinical context driving these state transitions.*  *---*  *## 6. Conclusion*  *This process mining analysis has provided a clear, data-driven view of the pathways leading to sepsis. We have moved beyond static risk factors to understand the dynamic journey of the patient.*  ***Summary of Findings:***  ** The core of the pre-sepsis process is characterized by significant **temperature instability**, primarily between normal and high temperatures.*  ** The **`High Temperature`** state is the most critical juncture, serving as the primary launchpad for progression to confirmed infection or sepsis.*  ** Significant time-based opportunities for intervention exist, most notably in the **7.1-hour transition from `High Temperature` to `Sepsis`** and the **6.1-hour transition to `Infection + High Temperature`**.*  ***Key Recommendations:***  *1. **Monitor Temperature Volatility:** Implement protocols to flag patients exhibiting repeated cycling between temperature states.*  *2. **Investigate High-Risk Transition Delays:** Perform a clinical deep-dive into the longest-duration transitions to understand and address potential delays in diagnosis or treatment.*  *3. **Refine Predictive Models:** Incorporate dynamic, transition-based features into sepsis prediction models.*  ***Next Steps:***  *We recommend scheduling a **collaborative workshop** with the clinical and epidemiological teams. The goal of this workshop will be to:*  ** Review these findings in detail.*  ** Validate the process map against clinical experience.*  ** Co-design and prioritize the recommended interventions and next steps for model development.*  *We are confident that by combining process analytics with clinical expertise, we can make significant strides in the early prediction and prevention of sepsis.* |
