# Supplementary material for: HealthProcessAI: a technical framework and proof-of-concept for LLM-enhanced healthcare process mining
Source: Front Artif Intell. 2026 Jan 30;9:1716819. doi: 10.3389/frai.2026.1716819 (PMC12901364; doi:10.3389/frai.2026.1716819)
Supplement: Supplementary file 1 [file Data_Sheet_1.ZIP › Supplementary Materials/Table S9.docx]

**Supplementary Table 9**

| **Case I Report_deepseek_deepseek-r1** |
| --- |
| *# Sepsis Progression Process Mining Analysis Report*  *## 1. Executive Summary*  ***Key Findings:***  *- **62% of cases** start in *Normal Temperature* or *High Temperature* states.*  *- **Critical pathway:** *High Temperature → Normal Temperature* is the most frequent transition (14,492 instances), but 7.5% of cases progress directly from *High Temperature* to *Sepsis* (909 cases, avg. 7.1 hours).*  *- **Process bottlenecks:** Recurrent loops between *High Temperature* and *Infection + High Temperature* (2,167 transitions, avg. 6.1 hours) suggest delayed infection resolution.*  *- **Early sepsis onset:** 17% of sepsis cases originate from *Normal Temperature* (202 cases, avg. 3.1 hours).*  ***Recommendations:***  *1. Prioritize early intervention for patients in *High Temperature + Infection* states to break feedback loops.*  *2. Implement real-time alerts for rapid sepsis detection in *Normal Temperature* patients with infection markers.*  *3. Standardize protocols for temperature management in suspected infections.*  *---*  *## 2. Introduction*  *This report analyzes sepsis progression using process mining to identify clinical pathways, delays, and opportunities for improvement. The dataset includes **1,206 cases** with events classified into five states: *Low Temperature*, *Normal Temperature*, *High Temperature*, *Infection + [Temperature State]*, and *Sepsis*. Transitions between states are reversible, reflecting the dynamic nature of sepsis.*  *---*  *## 3. Process Map Analysis*  *### Key Pathways*  *1. **Dominant Pathway:***  *`Start → Normal Temperature → High Temperature → Normal Temperature → Sepsis → End`*  *- Reflects cyclical temperature fluctuations before sepsis onset.*  *- *Normal Temperature → High Temperature* is the most frequent transition (14,940 instances).*  *2. **High-Risk Pathway:***  *`Start → High Temperature → Sepsis → End`*  *- Direct progression to sepsis occurs in **7.5% of cases** (909 instances) with a rapid median duration of **7.1 hours**.*  *3. **Infection-Driven Loops:***  *- *High Temperature ↔ Infection + High Temperature* (2,167 transitions) creates a 6.1-hour delay per loop, prolonging care.*  *### Top 5 Activities*  *\| Activity \| Frequency \| Role \|*  *\|---------------------------\|-----------\|-------------------------------------\|*  *\| Normal Temperature \| 16,209 \| Baseline state with frequent sepsis links \|*  *\| High Temperature \| 19,806 \| Critical escalation point to sepsis \|*  *\| Infection + High Temp \| 3,003 \| Delayed infection resolution \|*  *\| Sepsis \| 1,206 \| End state for all cases \|*  *\| Low Temperature \| 2,175 \| Less common, but linked to sepsis \|*  *---*  *## 4. Data Summary Tables*  *### Table 1: Case Summary*  *\| Metric \| Value \|*  *\|--------------------------\|-------------\|*  *\| Total cases \| 1,206 \|*  *\| Unique traces (variants) \| 82 \|*  *\| Median case duration \| 18.4 hours \|*  *\| Average case duration \| 27.6 hours \|*  *\| Shortest case duration \| 0 hours* \|*  *\| Longest case duration \| 143 hours \|*  **Cases starting directly at sepsis (n=1).*  *### Table 2: Activity Summary*  *\| Activity \| Frequency \| Median Time (hours) \| Avg. Time (hours) \|*  *\|---------------------------\|-----------\|---------------------\|-------------------\|*  *\| Normal Temperature \| 16,209 \| 1.2 \| 1.4 \|*  *\| High Temperature \| 19,806 \| 2.9 \| 3.1 \|*  *\| Infection + High Temp \| 3,003 \| 1.3 \| 1.9 \|*  *\| Sepsis \| 1,206 \| 0 \| 0 \|*  *\| Low Temperature \| 2,175 \| 0.5 \| 1.8 \|*  *### Table 3: Trace Summary*  *\| Trace (Simplified) \| % Cases \| Median Duration (hours) \|*  *\|----------------------------------------------\|---------\|--------------------------\|*  *\| Start → Normal Temp → High Temp → Sepsis \| 12% \| 7.1 \|*  *\| Start → High Temp → Normal Temp → Sepsis \| 10% \| 9.8 \|*  *\| Start → Normal Temp → Infection + High Temp → Sepsis \| 8% \| 14.3 \|*  *\| Start → High Temp → Infection + High Temp → Sepsis \| 6% \| 18.2 \|*  *\| Start → Normal Temp → Low Temp → Sepsis \| 4% \| 12.7 \|*  *---*  *## 5. Hypothesis for Sepsis Progression*  *### Key Insights*  *- **Hypothesis 1:** Delayed infection treatment in *High Temperature* states increases sepsis risk (evidenced by 6.1-hour loops).*  *- **Hypothesis 2:** *Normal Temperature* patients with undetected infections may rapidly deteriorate (17% sepsis cases).*  *- **Research Question:** Do early antibiotics for *Infection + High Temperature* reduce loop recurrence?*  *### Recommendations*  *1. **Predictive Monitoring:** Flag patients in *Normal Temperature* with elevated lactate/WBC for sepsis risk.*  *2. **Timebound Protocols:** Mandate infection workups within 3 hours for *High Temperature* states.*  *3. **Retrospective Analysis:** Audit cases with prolonged *Infection + High Temperature* loops for care gaps.*  *---*  *## 6. Conclusion*  ***Summary:***  *- Sepsis progression is highly variable, with critical delays in infection resolution.*  *- Temperature normalization does not eliminate sepsis risk, necessitating biomarker integration.*  ***Next Steps:***  *1. Host a clinical workshop to validate findings and refine protocols.*  *2. Pilot a sepsis prediction model using process mining thresholds.*  *3. Monitor compliance with timebound interventions for high-risk states.*  ***Collaboration Requested:***  *We invite clinical stakeholders to review these insights and co-design targeted quality improvement initiatives.* |
